# Surface Fermi Level Modulation of Photoanode by Optimized Conducting Nanoparticle Heterointerfaces for Enhanced Photoelectrochemical Water Splitting

**DOI:** 10.1002/advs.75734

**Published:** 2026-05-19

**Authors:** Phuong Thi Pham, Seulgi Ji, Unbeom Baeck, Yuankai Li, Duong Nguyen Nguyen, Won Tae Hong, Yang You, John Moraros, Kyoungsuk Jin, Jun Young Lee, Chan‐Hwa Chung, Tae‐Hoon Kim, Jongwook Park, Heechae Choi, Jung Kyu Kim

**Affiliations:** ^1^ School of Chemical Engineering Sungkyunkwan University (SKKU) Suwon Republic of Korea; ^2^ Institute of Inorganic and Materials Chemistry University of Cologne Cologne Germany; ^3^ Department of Chemistry and Research Institute of Natural Science Korea University Seoul Republic of Korea; ^4^ Advanced Materials Research Center (AMRC) & Department of Chemistry and Materials Science, School of Science Xi'an Jiaotong‐Liverpool University Suzhou Jiangsu China; ^5^ Department of Materials Science and Engineering, Engineering Research Center Chonnam National University Gwangju Republic of Korea; ^6^ Integrated Engineering, Department of Chemical Engineering Kyung Hee University Yongin South Korea; ^7^ SKKU Advanced Institute of Nanotechnology Sungkyunkwan University (SKKU) Suwon Republic of Korea

**Keywords:** facilitator, fermi level modulation, metal oxide photoanode, metal phosphide, photoelectrochemical water splitting

## Abstract

Deposition of conducting nanoparticles on metal oxide semiconductor electrodes has been harnessed as an effective strategy to enhance photoelectrochemical (PEC) water splitting performances. However, the PEC water splitting activities of semiconductor electrodes covered with conducting nanoparticles show significantly irregular behaviors with defect concentrations, choice of nanoparticle material, and coverages. Herein, we retrieve the rational positioning of metallic nickel phosphide (Ni_2_P) nanosphere on a BiVO_4_ (BVO) with an optimal coverage of Ni_2_P and report drastically improved PEC oxygen evolution reaction (OER). Our multi‐scale (MS) simulation method theoretically predicts that the range of heterointerface contact coverage is 6%–11% on the BVO, which can be the optimal positioning condition for the high PEC OER, nevertheless Ni_2_P possesses the poor OER kinetic. In our experimental results, the Ni_2_P/BVO with an optimal Ni_2_P coverage of 9.4% presents significantly enhanced photocurrent density at 1.23 V_RHE_ under 1 sun illumination, which is in good agreement with the theoretical prediction. Density functional theory calculations and our analytic model reveal that the contact coverage of Ni_2_P nanospheres on the surface of BVO manipulates the Fermi level of BVO, which tailors the surface reaction kinetics of OER. This work provides a novel design strategy of nanoparticle‐deposited photoelectrodes for PEC responses.

## Graphical abstract

Optimizing the heterointerface coverage of the conducting Ni_2_P nanoparticles on the surface of BVO photoanode manipulates the surface Fermi‐level modulation, which significantly enhances photoelectrochemical oxygen evolution reaction. Our multiscale simulations and experimental results reveal that an optimal Ni_2_P coverage of 9.4% pins the surface Fermi level higher, which is beneficial to PEC water splitting on BVO surface by lowering the surface reaction energy barrier.

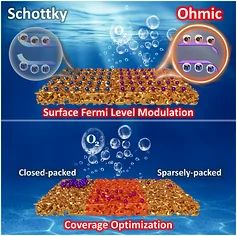

## 1 Introduction

Photoelectrochemical (PEC) water splitting has drawn great research interest as a promising technology for efficiently producing highly sustainable hydrogen (H_2_), an important alternative candidate owing to its high energy density and zero carbon emissions [1, 2, 3, 4, 5]. In the PEC system, the use of n‐type metal oxide (ex. BiVO_4_, WO_3_, α‐Fe_2_O_3_) as a photoanode is preferred due to their favorable absorption coefficient, charge separation properties, environment‐friendliness, and earth‐abundance [6, 7, 8]. However, it suffers from their sluggish surface catalytic kinetics for oxygen evolution reaction (OER) and undesired rapid charge recombination due to the short charge migration length, which impedes their PEC performance for practical application [9, 10, 11, 12, 13, 14, 15]. To address these drawbacks, the introduction of a transition metal‐based cocatalyst overlayer on the surface of the photoelectrode has been widely used with intentions to provide abundant active sites for PEC OER [16, 17, 18]. Specifically, it has been extensively adopted that the extraction of photoinduced holes from the photoanode to the overlaid co‐catalysts — providing the active sites for OER — promotes the thermodynamic charge migration, resulting in the efficient charge separation and PEC performances [17, 19].

However, in the case of introducing metallic nanoparticles (NPs) such as transition metal phosphides on the surface of the photoanode, it was observed that the optimal coverage level of these NPs significantly contributes to enhancing the PEC water oxidation activity, unlike the widely used cocatalysts [20, 21, 22]. Surprisingly, even without intrinsic OER catalytic activity themselves, these decorated nanoparticles successfully improve the PEC performance of the photoanode [23, 24, 25, 26]. Nevertheless, the understanding of that mechanism is lacking, along with fundamental questions regarding the enhanced PEC activity of metallic nanoparticle‐decorated photoanodes remain: i) If the active sites for PEC water splitting are on the surface of photoanodes, why do the decoration of metallic nanoparticles enhance the catalytic activity of photoanodes? ii) Why do the optimum coverage of metallic nanoparticles exist if the active sites are on the metallic NPs, rather than full coverage?

In this study, we demonstrate that surface Fermi level control via the Fermi level pinning effect can serve as a dominant factor governing the PEC water oxidation activity of BiVO_4_ (BVO) photoanodes, by forming an Ohmic junction with Ni_2_P nanoparticles (Ni_2_P/BVO). Although the Fermi level pinning effect is generally considered detrimental due to limited band bending and suppressed charge separation, we show that this effect can be strategically utilized to achieve enhanced catalytic performance [27, 28, 29]. Unlike cocatalysts, the spherical Ni_2_P NPs act as a facilitator, promoting the surface reaction kinetics of the BVO photoanode, a widely studied n‐type semiconducting metal oxide in PEC OER [30, 31, 32]. With density functional theory (DFT) calculations and our new analytic model, we revealed that the contact coverage of Ni_2_P NPs on the surface of BVO manipulates the Fermi level of BVO, which tailors the surface reaction kinetics of OER of BVO. For our theoretical prediction, the range of the heterointerface contact coverage was 6%–11% on the BVO photoanode, which can be the optimal positioning condition for facilitating the high PEC OER. This calculation was in good agreement with our experimental results of PEC performance, where we achieved outstandingly high PER OER performance by incorporating the Ni_2_P with the optimal coverage of 9.4%. Furthermore, to promote the OER activity, NiFeO_x_ overlayer had been introduced on the surface of the Ni_2_P/BVO, inducing the efficient extraction of photo‐generated holes and provides the BVO with the abundant active sites for OER [33]. Consequently, the resulting NiFeO_x_/Ni_2_P/BVO hybrid photoanode at the optimal coverage of Ni_2_P achieved a photocurrent density of 5.49 mA cm^−2^ at 1.23 V versus reversible hydrogen electrode (RHE) under 1 sun illumination, which was 3.4‐fold higher than that of the pristine BVO. An apparent cathodic shift of the onset potential (180 mV) from 2.26 V_RHE_ for BVO to 2.08 V_RHE_ for Ni_2_P/BVO in dark condition directly demonstrates that the Ni_2_P accelerated the water oxidation kinetic and lowered the required overpotential. This work suggests the principles for the rational regulation of metallic NPs on the surface reactivity of photoanode, which provides a strategy for designing high‐efficiency photoanodes in solar water‐splitting systems.

## 2 Results and Discussion

### 2.1 Theoretical Predictions

The hole carrier transport from the VBM of BVO to the BVO surface is an important factor of PEC water splitting activity [8, 34]. However, when Fermi level pinning is properly made, the BVO photoanode can have high PEC water oxidation activity even with flattened surface band or even with ohmic junctions, which cannot induce the improved rate of photoexcited hole carrier transport [35, 36]. Therefore, we have a key hypothesis that the water oxidation reaction on a BVO photoanode can be dominated by the surface Fermi level control. Accordingly, in this modeling, we assumed that PEC water oxidation reactions on a BVO photoanode can occur with and without hole carrier delivered from the VBM to the surface of BVO (with and without illumination).

**FIGURE 1:**
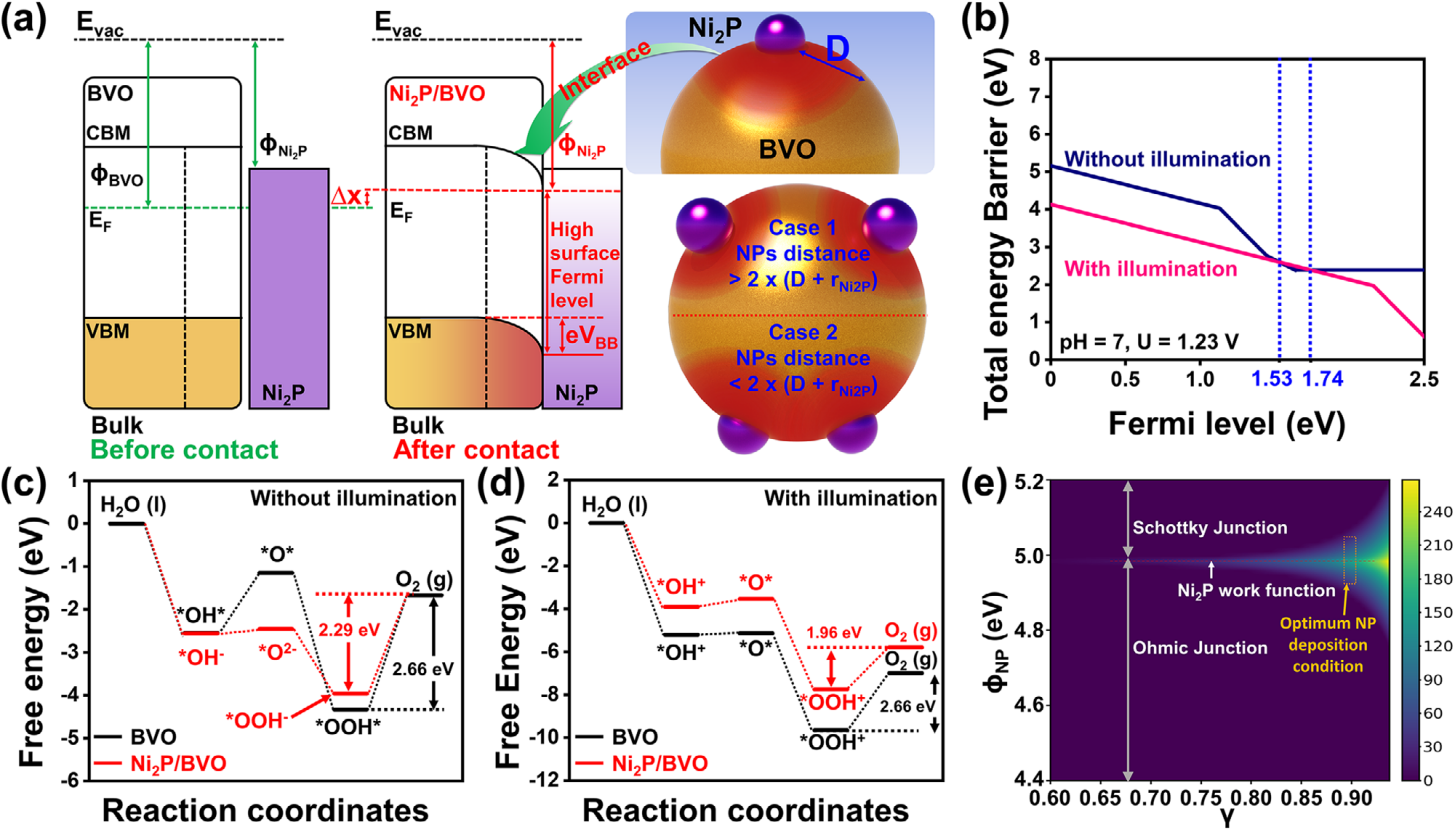
Fermi level dependency of water oxidation activity of BVO photoanode. (a) schematic of the BVO surface Fermi level control with Ni_2_P deposition and explanation of two cases of Ni_2_P NPs loading where the distance between Ni_2_P NPs is shorter/longer than twice the sum of the charge depletion region width (*D*) and the radius of the Ni_2_P NPs contact area ($r_{Ni_{2}P}$). (b) The calculated total energy barrier for water splitting without and with illumination at pH = 7 and U = 1.23 V. The calculated OER energy diagram on bare BVO and Ni_2_P/BVO (c) without and (d) with illumination, which are consistent with the surface Fermi level of BVO at 1.1 eV (black lines) and 1.8 eV (red lines), respectively. Analytical predictions of the relation between Ni_2_P NPs dispersion and the PEC OER reaction rate. (e) The calculated surface reaction rate ratio in the PEC water oxidation on BVO as a function of BVO active site ratio (𝛾) and work function of the NP species (Φ_NP_) in a color contour.

To demonstrate the hypothesis, Fermi level dependency of water oxidation activity of the BVO photoanode, theoretical predictions of the energy barriers and reaction energy diagrams for the BVO photoanode with and without illumination (with and without hole delivery from VBM to surface) were made in advance (Figure 1). Recently, the surface Fermi level pinning method was actively used for BVO photoanode to boost PEC water oxidation reaction even with metallic NPs, which reduces the surface band bending of BVO [24, 37, 38]. In addition, photoanode designs for metal‐semiconductor junctions (both for Ohmic and Schottky types) have been made with assumptions that the Fermi level equilibration in the heterojunctions are made to the Fermi energy of the metal particle. However, it is obvious that the equilibrium Fermi level in metal‐semiconductor junctions can deviate from the conventional model when the metal particles are in nanometer‐scale, due to the limited number of free electrons. The atomic‐scale reaction mechanisms in the photochemical water splitting reactions with the surface band bending effects were not clearly stated in the previous works. Based on our theoretical prediction that the surface reaction energy barrier of water oxidation is reduced with a higher surface Fermi level of BVO photoanode, in this study, we chose Ni_2_P as the catalyst on BVO by adjusting the surface Fermi level of BVO as presented in Figure 1a. In addition, we considered the variation of the Fermi energy of metal NPs, which is caused by the size effect (Figure 1a). In addition, we considered the changed Fermi level position of BVO as a function of distance from a deposited Ni_2_P NP within the charge depletion region was also considered.

As shown in Figure 1b, we calculated the total energy barriers versus the Fermi level for water oxidation on BVO surfaces at pH = 7 and with a bias of 1.23 V, with and without illumination. The Fermi‐level‐dependent total energy barriers for water splitting by BVO with and without illumination were obtained from the calculated adsorption energies of water oxidation intermediates on BVO. The optimized adsorption configurations of the water splitting intermediates on BVO are shown in Figure S1. In most of the atomic‐scale modeling approaches to water oxidation reaction on BVO surface [39] under illumination, only neutral charge states of the intermediate adsorbates were considered, even though the Fermi energy in a normal DFT calculation is set to the VBM of BVO [40, 41]. To take the hole transfer into account of reaction energetics, we added the term for the potential difference between the VBM of BVO and the proton reduction potential level, as described in Equation S1. Employing our Fermi level dependent adsorption energy models, we found that the charge states of all the OER intermediates, OH*, O*, OOH* have charge transitions at Fermi levels of 1.45, 1.13, 1.64 eV, respectively, when hole carrier transfer from VBM to the water oxidation potential level is not involved in the reaction (Figure S1). Therefore, surface Fermi level change from a normal BVO (1.1 eV) to a higher level (>1.64 eV) can change the PEC water oxidation reaction even without aids of hole carrier delivered from the VBM of BVO. When a hole carrier transfer from the VBM level of BVO by a photoexcitation is involved (with illumination in Figure 1a), the total energy barrier of water oxidation reaction on BVO was found to be lower than that of the reactions without hole transfer between BVO VBM and water oxidation potential level (without illumination), except the case that BVO surface Fermi level spans over a narrow range (1.53–1.74 eV). This theoretical prediction is consistent with most of previous experimental observations, where the water oxidation activity of BVO is improved with higher hole carrier transport efficiency.

In the Fermi range between 1.53 and 1.74 eV, the total energy barrier for water oxidation on BVO without a hole transferred from the VBM of BVO (without illumination) is rather slightly lower (difference up to 0.11 eV at the BVO Fermi level of 1.64 eV) than the reaction with a hole carrier (with illumination). Here, we can reason two new material engineering directions for boosting PEC water oxidation activity of metallic NPs deposited BVO: 1) high Fermi level at BVO surface can reduce the total energy barrier of surface reactions, and 2) the activity of the PEC water oxidation on a BVO photoanode with certain surface Fermi level range (1.53–1.74 eV) will not be significantly affected by the hole carrier transport. Very importantly, the BVO‐based photoanode material design direction to reduce the surface reaction energy barrier of water oxidation with a higher surface Fermi level conflicts with the other key material design principle, hole carrier transport, because a larger band bending of a BVO photoanode induce a lower surface Fermi level.

To make the Ni_2_P metallic NPs valid for the proof of our hypothesis, it is necessary to investigate whether the surface of BVO would dominantly contribute to the water oxidation reaction as the main active site. Accordingly, we constructed a Ni_2_P slab model and calculated the electrocatalytic OER energy diagram on Ni_2_P (Figure S2). The optimized adsorption configurations are shown in Figure S2. It was predicted that water oxidation reaction on Ni_2_P surface does not contribute as an active site for the water oxidation reaction activity because of the high energy barrier (5.11 eV) in OER with a bias of 1.23 V. Thus, we can assume that the surface of BVO in Ni_2_P/BVO a heterojunction mainly contributes to the active sites of PEC water oxidation. The adsorption energies of OER intermediates on Ni_2_P‐deposited BVO surface with consideration of the surface Fermi level pinning by Ni_2_P deposition on the BVO surface were plotted (Figure S3). The surface reaction energy barriers for water oxidation are predicted to be reduced by deposition of Ni_2_P NPs on BVO both without and with illumination (Figure 1c,d). For direct comparison, we constructed an explicit Ni_2_P/BVO interfacial model to examine the adsorption configurations of OER intermediates at the interface and on the Ni_2_P site (Figure S4). Using this model, we considered two possible adsorption sites according to energetic preferences: the Bi site near the Ni_2_P/BVO interface and the P site on Ni_2_P. At both sites, the OOH* intermediates were not stabilized and dissociated, indicating that the enhanced OER activity of BVO by the formation of the Ni_2_P/BVO interface cannot be supported or predicted by conventional Ni_2_P/BVO interfacial models. The OER activity of the BVO photoanode was therefore evaluated using a bare BVO slab model and our Fermi‐level‐dependent adsorption energy model based on the BVO surface, which enables consistent and reliable thermodynamic comparisons of OER intermediates on the BVO surface with and without Ni_2_P deposition.

**FIGURE 2:**
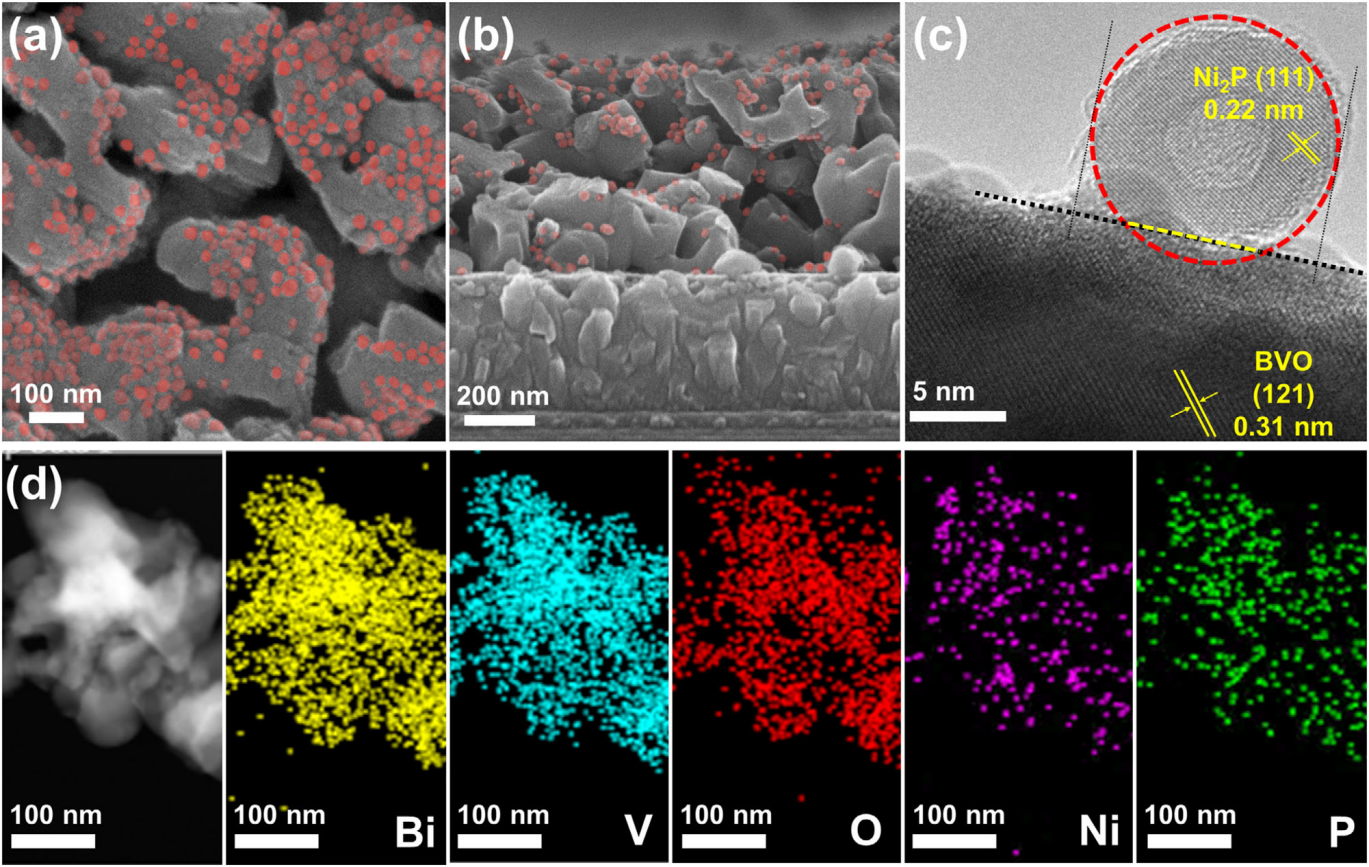
Morphology analysis. (a) SEM top‐view image, (b) SEM cross‐section image of Ni_2_P/BVO, where red implies that the Ni_2_P NPs; (c) HRTEM image of Ni_2_P/BVO photoanode; (d) EDS element mapping of Ni_2_P/BVO with the distribution of Bi, V, O, Ni, and P.

When a metallic NPs is deposited on a semiconductor, band bending is mostly induced either downward (Ohmic) or upward (Schottky). Since Ni_2_P NPs loading on BVO reduces the work function of BVO by Fermi level pinning (∆x), it is reasoned that Ni_2_P and BVO has an Ohmic contact with a downward band bending (eV_BB_) of BVO in our Ni_2_P/BVO sample (Figure 1a). We can predict that a high surface Fermi level of BVO pinned by Ni_2_P is beneficial to PEC water splitting on the BVO surface because of the lowered surface reaction energy barrier. However, the downward surface band bending of BVO by the Ohmic junction with Ni_2_P is expected to weaken the hole carrier transport [42]. Figure 1e shows a surface reaction rate ratio prediction for PEC water oxidation on BVO as a function of the work function of a deposited NP on BVO. In the analytic model, a 2.5 nm radius of the area of Ni_2_P contact with the BVO surface was used (shown in Figure 2c below). Considering the Fermi level‐dependent adsorption energy model, given that the surface Fermi level of BVO can be altered within the depletion region as the distance from Ni_2_P is changed, adsorption energy is accordingly affected. Thus, we can regard the depletion region as an active site, where the catalytic activity is influenced by surface Fermi level. The ratio of the surface reaction rate of water oxidation on Ni_2_P/BVO without/with illumination (R_1_) and on bare BVO without illumination (R_0_) can be obtained by using the following equation:

(1) $$\begin{array}{cc} \frac{R_{1}}{R_{0}} & =\gamma \left[\int_{r_{Ni_{2}P}}^{r_{Ni_{2}P}+D}\exp\left(\frac{{\Delta E}_{\mathrm{barrier}}^{0}-{\Delta E}_{\mathrm{barrier}}^{1}}{k_{B}T}\right)2\pi r\mathrm{dr}\right.\\ & \;+\left.\int_{r_{Ni_{2}P}+D}^{r}\exp\left(\frac{{\Delta E}_{\mathrm{barrier}}^{0}-{\Delta E}_{\mathrm{barrier}}^{1}}{k_{B}T}\right)2\pi r\mathrm{dr}\right] \end{array}$$

where r_Ni2P_, D, and r are the radius of Ni_2_P, depletion region width, and the average distance of two Ni_2_P NPs, respectively. $E_{barrier}^{0}$ and $\Delta E_{barrier}^{1}$ in Equation (1) indicates the total energy barrier of water oxidation on bare BVO without illumination and that of Ni_2_P/BVO without/with illumination (Figure 1b).

The term γ in Equation (1) is the ratio of the exposed BVO active site in Ni_2_P/BVO photoanode, hence, is determined by the Ni_2_P distance. According to our computation results, we can predict that a high surface Fermi level (1.8 eV) of BVO pinned by Ni_2_P is beneficial to PEC water splitting because of the reduced surface reaction energy barrier, as the drawback of reduced hole carrier delivery by the downward surface band bending of BVO in the Ohmic junction with Ni_2_P is compensated with higher surface reaction rate. In Figure 1e, the optimum deposition conditions are depicted for the cases where the choice of metallic NPs materials and dispersion distance can vary simultaneously. According to our theoretical prediction from the combinations of DFT calculation (Figure 1b) and the analytic model in Equation (1), the Ni_2_P turned out to enhance the overall water oxidation reaction of BVO when loaded with coverage less than 11% (γ > 89%) because the work function variation of Ni_2_P can is at least 0.05–0.1 eV [43]. Higher coverage (low γ) allows only narrower ranges of work function to improve the PEC water splitting by BVO, which can result in no difference in the PEC activity during the reaction. The light scattering effect [44] with high coverage of Ni_2_P on BVO and the incomplete Fermi level equilibration with low coverage Ni_2_P, which arise from the particle size difference between Ni_2_P facilitator and BVO host materials [45] were not included in the analytic model (Equation (1)). In addition, when the coverage of Ni_2_P is too small on BVO, the Fermi level equilibration and Ohmic‐junction formation is incomplete. Considering the electron density of states of Ni_2_P (Figure S5), we found that the minimum coverage of Ni_2_P on BVO is 6% to meet the completed Ohmic junction between Ni_2_P and BVO using the following equation: [46]

$$N\int_{E_{Fermi}-\Delta \emptyset }^{E_{Fermi}}D\left(E\right)FD\left(E\right)dE=N_{C}\exp\left(\frac{\Delta \emptyset }{kT}\right)$$

(2) $$N=4\cdot \left(1-\gamma \right)\cdot {\left(\frac{R_{BVO}}{R_{Ni_{2}P}}\right)}^{2}$$

where N, D(E), FD(DE), N_C_, E_Fermi_, Δ∅, Δε_*F*_ are the number of Ni_2_P particles deposited on a BVO particle, electron DOS, Fermi‐Dirac distribution, electron concentration of CBM, Fermi energy of Ni_2_P, work function difference between Ni_2_P and BVO, respectively. Therefore, the theoretical prediction in Figure 1e of the optimum Ni_2_P coverage range (6%–11%) is expected to be wider than the experimental results.

### 2.2 Morphology and Structure of Ni_2_P/BVO

**FIGURE 3:**
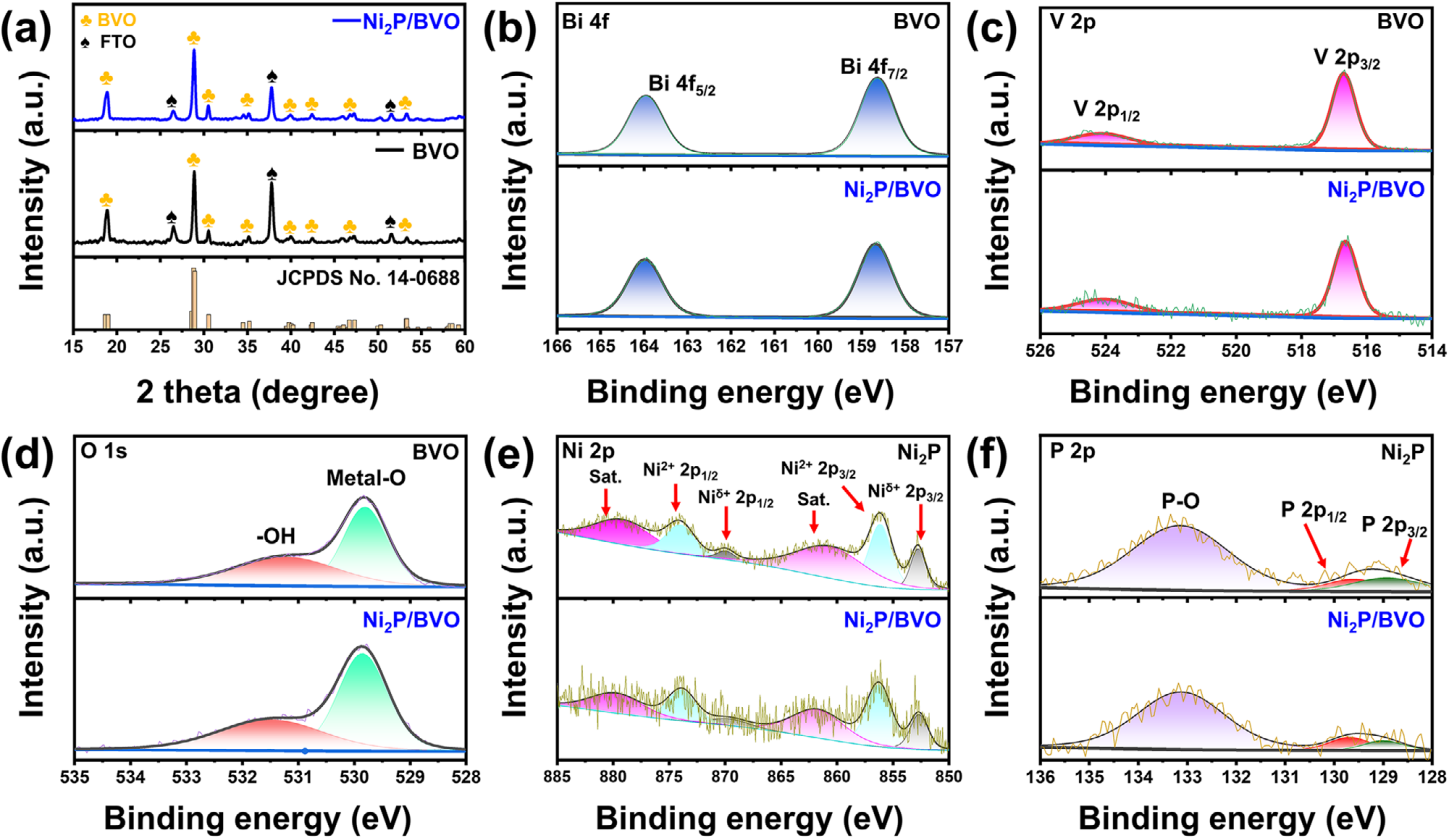
Structure characterizations (a) XRD patterns of BVO, Ni_2_P/BVO photoanodes; XPS high‐resolution (b) Bi 4f, (c) V 2p, (d) O 1s, (e) Ni 2p, and (f) P 2p spectra for BVO, Ni_2_P/BVO.

The nanoporous photoanode and Ni_2_P NPs were fabricated via facile process each other. The nanoporous BVO with thickness of 600 nm and a worm‐like shaped structure of which grain size of ca. 300 nm was synthesized on the surface of fluorine doped tin oxide (FTO) glass substrate via the solvothermal method shown in Figure S6. Meanwhile, the Ni_2_P NPs were synthesized by a hot‐injection method, which have a nearly spherical morphology and an average diameter of ca. 16 nm (Figure S7). After the incorporation of Ni_2_P NPs on the surface of the BVO photoanode, the color of photoanode changed from light yellow to an overall dark yellow (Figure S8). The scanning electron microscopy (SEM) images shown in Figure 3a,b further present that the discreet particles were introduced on the surface of photoanode conformally. High‐resolution transmission electron microscopy (HR‐TEM) was conducted to investigate the morphology and lattice of Ni_2_P and BVO based samples. Figure S9 presents that the BVO has lattice spacing of 0.28 nm indicating (004) plane and energy dispersive spectroscopy (EDS) elemental mapping, revealing the uniform distribution of Bi, V, and O elements [12]. Meanwhile, the crystalline structures of the Ni_2_P/BVO in Figure 3c reveals that the Ni_2_P NPs on the surface of BVO display clear lattice fringes with a spacing of 0.22 nm, consistent with the Ni_2_P (111) plane and EDS line profiling to determine the chemical composition of each element shown in Figure S10 indicates that the loaded NP has Ni and P with 2: 1 atomic ratio, confirming the successfully loading Ni_2_P NPs on BVO with no structure transformation [47]. Likewise, as depicted in Figure 2d, EDS elemental mapping was analyzed to determine the chemical composition of each element, which presents that Bi, V, O, Ni, P were evenly distributed on the Ni_2_P/BVO nanoparticles in overall region.

To investigate the crystal structures of BVO, Ni_2_P/BVO, and Ni_2_P, X‐ray diffraction (XRD) patterns were measured as shown in Figure 3a and Figure S11. The three peaks located at nearly 26.5°, 37.8°, 51.5° were assigned to the (110), (101), and (211) planes of the FTO substrate (JCPDS#46‐1088). Meanwhile, the diffraction peaks at 18.9°, 28.8°, 30.5°, 35.2°, 39.9°, 42.4°, 47.2° and 53.3° correspond to those of BVO (JCPDS#14‐0688), indicating the high crystallinity with the monoclinic phase of synthesized BVO. After introducing the Ni_2_P NPs on the surface of photoanode, there is no significant difference in the XRD pattern of Ni_2_P/BVO compared to the that of pristine BVO even through the Ni_2_P NPs have high crystalline structures (JCPDS#74‐1385) [47]. It reveals that the Ni_2_P catalysts are evenly distributed on the surface of BVO with no aggregation, which was consistent with the SEM images of Ni_2_P/BVO.

High‐ resolution XPS measurement was carried out to analyze the surface chemical states of Ni_2_P/BVO, and BVO for comparison. The binding energy of the C‐C bond was standardized at 284.8 eV to calibrate XPS data (Figure S12). In detail, the peaks observed at 158.6 and 163.9 eV correspond to Bi 4f_7/2_ and Bi 4f_5/2_ orbitals of Bi^3+^ of the pristine BVO (Figure 3b) [38, 48]. For the V 2p peaks of pristine BVO and Ni_2_P/BVO, the two peaks with the binding energies of 516.7 and 524.0 eV belong to V 2p_3/2_ and V 2p_1/2_ of V^5+^ (Figure 3c). In the case of the O 1s region, the peaks at 529.8 and 531.5 eV present the metal‐O bonding and ‐OH bond from the surface‐absorbed water, respectively (Figure 3d) [49, 50, 51]. In Ni 2p XPS spectra for Ni_2_P (Figure 3e), two spin orbit doublets at 852.9 eV (2p_3/2_) and 870.1 eV (2p_1/2_) belong to the binding energy of Ni species in alloyed Ni_2_P (0 < δ < 2) [52]. Likewise, the Ni^2+^ peaks at 856.2 and 874.2 eV and two satellite peaks at 861.2 and 879.7 eV can be assigned to the oxidized Ni species related to the surface oxidation of Ni_2_P. In terms of the P 2p spectrum, three peaks are located at 129.0, 129.7, and 133.3 eV; the former two doublets are attributed to the binding energy of metal‐P bonds in metal phosphides, and the other peak can be assigned to oxidized P species, as has been reported previously (Figure 3f) [53, 54]. Compared to Ni_2_P/BVO with the pristine BVO and Ni_2_P, respectively, there is no significant peak shift in Bi 4f, V 2p, O 1s, Ni 2p, and P 2p spectra after the introduction of Ni_2_P NPs on the surface of BVO, which implies that there is no chemical interaction between Ni_2_P and BVO.

### 2.3 Photoelectrochemical Water Splitting Performance

**FIGURE 4:**
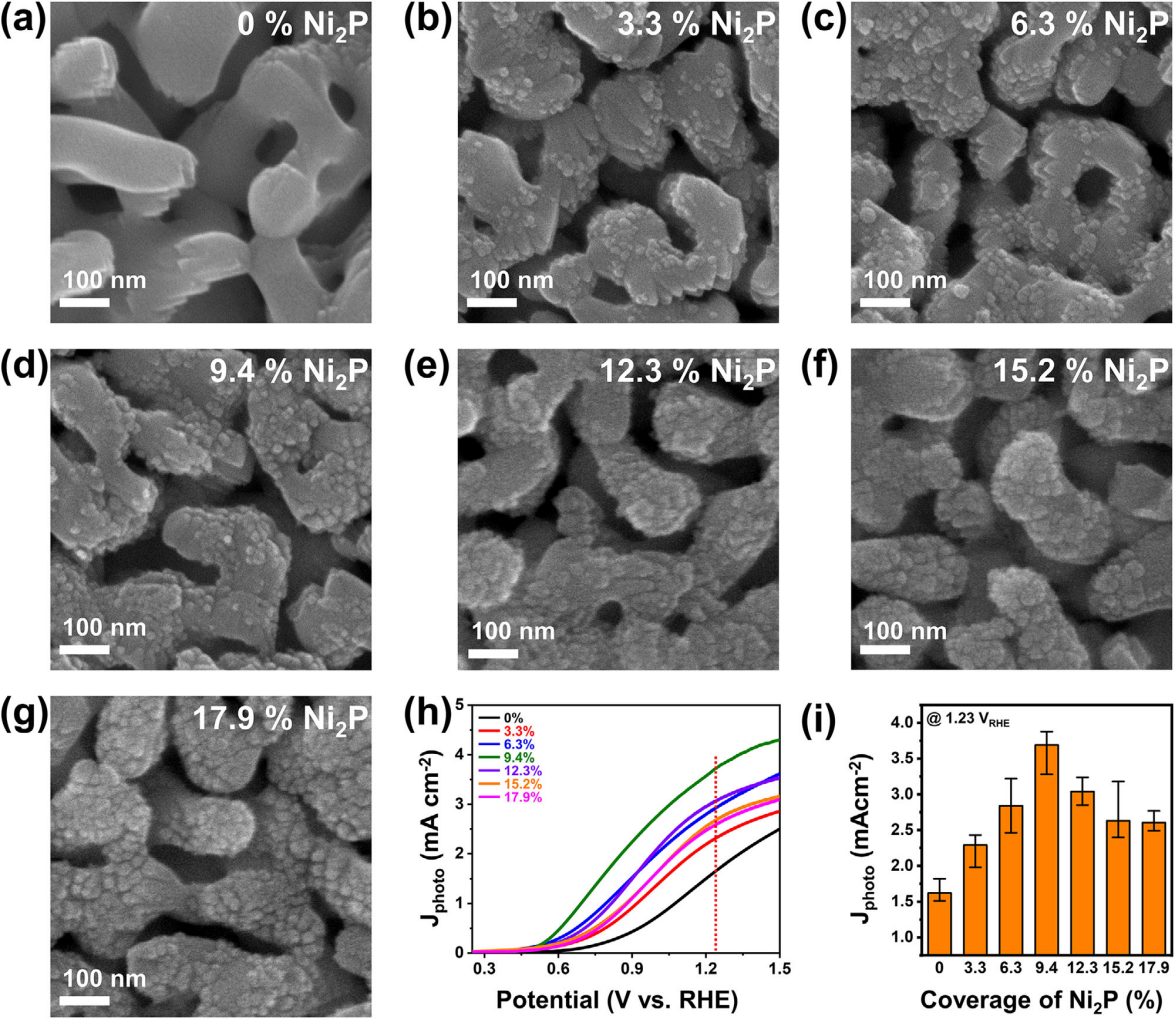
The effect of the Ni_2_P‐coverage on the BVO surface for PEC water oxidation performance. (a‐g) SEM images of BVO, Ni_2_P‐x%/BVO samples with different Ni_2_P coverages (x% represents the coverage of Ni_2_P on BVO, determined by the ratio between the area of Ni_2_P contact with the BVO surface and the total area of the BVO surface). (h) J‐V curves of Ni_2_P/BVO with different Ni_2_P coverages and (i) statistics of the current densities of BVO, Ni_2_P‐x%/BVO photoanodes at 1.23 V_RHE_.

To study the coverage effect of Ni_2_P NPs on the BVO photoanode, different loading amounts were applied on the surface of BVO. For aiming to limit the aggregation of Ni_2_P NPs on the BVO surface, we used chloroform as a solvent to effectively disperse Ni_2_P NPs. Afterward, the coverage percentage of Ni_2_P NPs on BVO was established by measuring the areas of BVO and Ni_2_P particles from the SEM images via the freeware ImageJ. The radius of the contact area of Ni_2_P with BVO was approximately 0.55 times the radius of spherical Ni_2_P NPs (Figure 2c). As shown in Figure 4a–g, Ni_2_P NPs were well disseminated and anchored on the BVO surface, forming an overlayer without significant agglomeration. The surface coverage percentage of Ni_2_P NPs with different loading amount on BVO was estimated with an increase from 3.3% to 17.9%. It should be noted that a utilization of a higher coverage percentage corresponding with that of Ni_2_P, higher than 9.4% could induce an unintended aggregation between Ni_2_P NPs. To investigate the effect of agglomerated Ni_2_P NPs induced by application of excess coverages on the BVO for photoelectrochemical water oxidation catalytic activity, the photocurrent density at 1.23 V_RHE_ of such Ni_2_P/BVO photoanodes with various coverages was evaluated using the linear sweep voltammetry (LSV) in 0.1 M potassium phosphate (KPi) electrolyte under AM 1.5 G backside illumination (100 mW cm^−2^) as shown in Figure 4h and summarized in Figure 4i. Obviously, the enhancement of PEC catalytic activity of Ni_2_P/BVO photoanodes was achieved according to increase of Ni_2_P concentration up to 9.4%. Meanwhile, the enhancement trend of that was reversed after introducing Ni_2_P NPs over 9.4% coverage on BVO, which indicates that the Ni_2_P/BVO with 9.4% coverage shows the outstandingly highest photocurrent density performance among the Ni_2_P/BVO photoanodes with various coverage of Ni_2_P from 3.3% to 17.9%. This tendency of photocurrent density of Ni_2_P/BVO is strong evidence that active site for PEC OER is the surface of BVO, and Ni_2_P serves as a facilitator for prompting surface reaction kinetics (Figure 1b and Figure S2). Moreover, the existence of the optimum Ni_2_P coverage (∼9.4%) is consistent with our analytic model prediction as mentioned above in Figure 1e, which explains that the optimal PEC performance can be obtained by introducing Ni_2_P NPs on the surface of BVO with the coverage range (6%–11%). The trend of photocurrent density enhancement of photoanode up to 9.4% of Ni_2_P‐coverage on BVO can be explained with completed Fermi level equilibration by electron injection from Ni_2_P particle to the BVO surface forming a slight Ohmic junction (Figure 1a) and reduced reaction energy barrier (Figure 1b).

**FIGURE 5:**
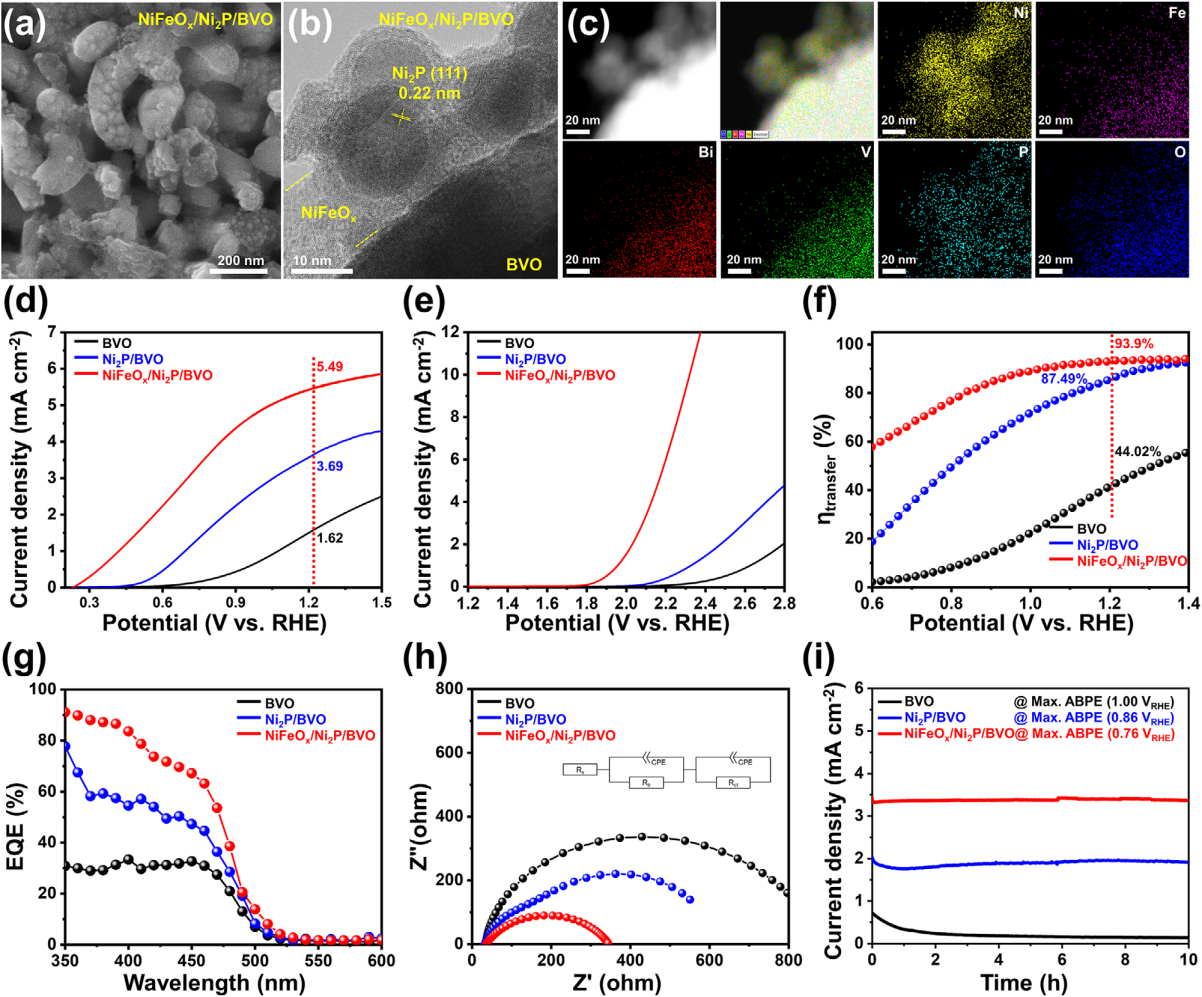
(a) SEM image, (b) TEM image, and (c) EDS elemental mapping of NiFeO_x_/Ni_2_P/BVO. (d, e) J‐V curves of BVO, Ni_2_P/BVO, and NiFeO_x_/Ni_2_P/BVO with and without illumination. (f) Charge transfer efficiency, (g) IPCE spectra, and (h) Nyquist curves with bias at 1.23 V_RHE_ under illumination and (inset) the equivalent circuit model of the BVO‐based photoanodes (R_s_, R_tr_, R_ct_, CPE corresponding to solution resistance, charge transport resistance, resistance, charge transfer resistance, and constant phase element, respectively). (i) The long‐term photocurrent density versus time curves at the potential of the maximum ABPE value.

**FIGURE 6:**
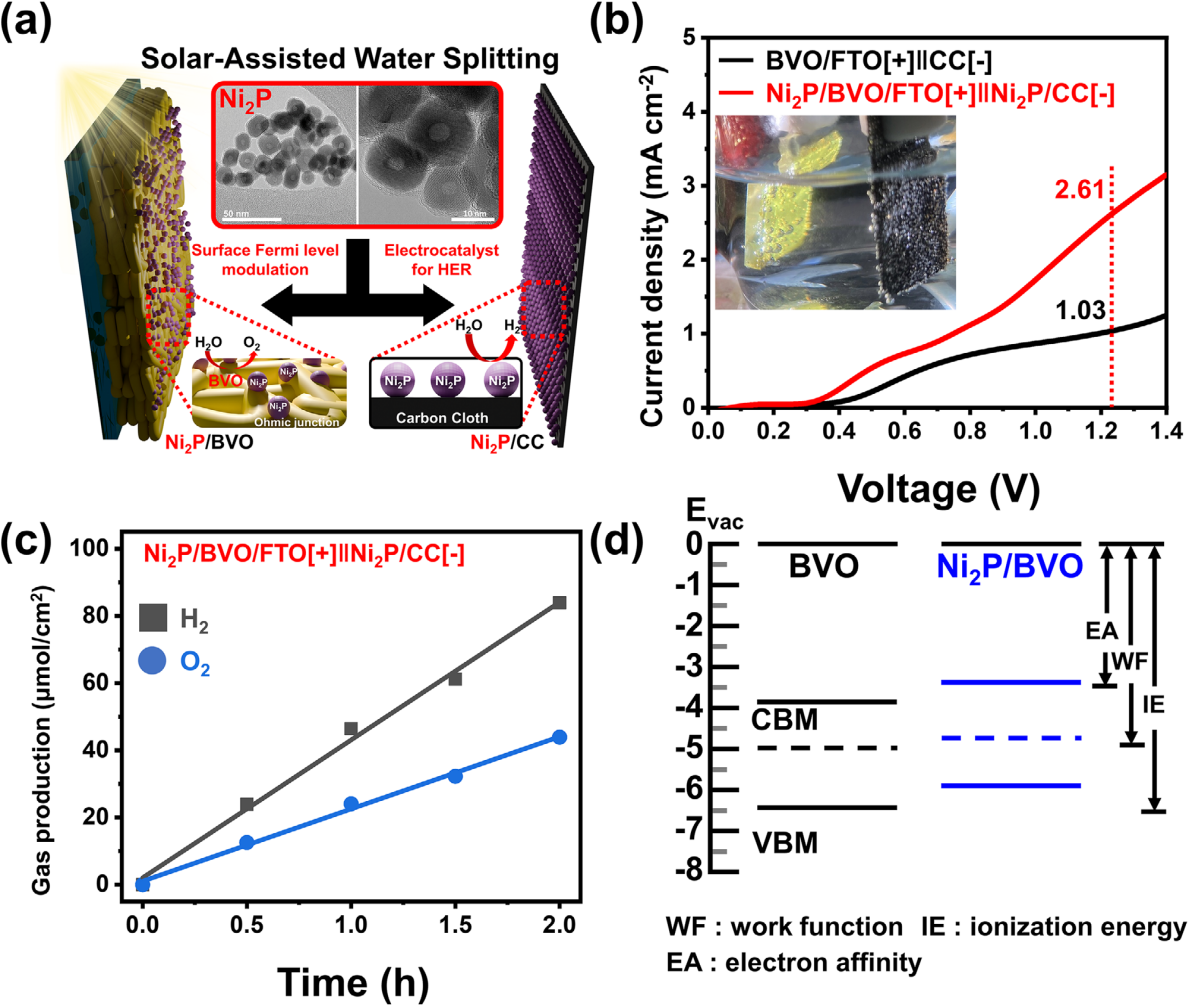
(a) Schematic illustration of solar‐assisted overall water splitting cell of Ni_2_P/BVO/FTO[+] versus Ni_2_P/CC[‐] under 1 sun illumination and (b) J‐V curves of solar‐assisted water splitting cell under AM 1.5 G 1 sun illumination of BVO/FTO[+]||CC[‐] and Ni2P/BVO/FTO[+]||Ni_2_P/CC[‐]. (c) H_2_ and O_2_ gas production rate of fabricated electrodes. (d) Band structure diagram of BVO and Ni_2_P/BVO.

To maximize the photoelectrochemical water oxidation efficiency, a NiFeO_x_ overlayer with outstanding catalytic activity for OER was introduced on the surface of Ni_2_P/BVO. The NiFeO_x_ layer with about 10 nm thickness was successfully decorated on the surface of BVO as shown in Figure S13a and EDS line profiling demonstrates that overlayer is composed of Ni, Fe, and O (Figure S13b). Likewise, as depicted in Figure 5a,b, the NiFeO_x_ overlayer was coated on the surface of Ni_2_P/BVO without structural destruction of Ni_2_P, confirmed by EDS line and mapping profiling (Figure 5c and Figure S14), which presents that Ni, P were distributed on Ni_2_P, and Bi, V, O, Fe were evenly distributed on NiFeO_x_/Ni_2_P/BVO. XRD and XPS analysis of NiFeO_x_/BVO and NiFeO_x_/Ni_2_P/BVO were conducted and summarized in Figures S15–S17. As shown in Figure 6d, the Ni_2_P/BVO photoanode yields a 2.3‐fold higher photocurrent density of 3.69 mA cm^−2^ at 1.23 V_RHE_ in 0.1 M KPi electrolyte than that of 1.62 mA cm^−2^ for the pristine BVO, which indicates that the introduction of Ni_2_P NPs on the surface of BVO dramatically boosts the PEC water oxidation performance. Notably, after decoration of NiFeO_x_ overlayer on the Ni_2_P/BVO and BVO shown in Figure 5d and Figure S18, the photocurrent density of 5.49 and 3.88 mA cm^−2^ at 1.23 V_RHE_ were achieved, respectively, which presents the 3.4‐fold enhanced performance of NiFeO_x_/Ni_2_P/BVO compared to the bare BVO, revealing the synergetic effects of NiFeO_x_ with Ni_2_P for OER. For the sake of understanding the catalytic kinetics of water oxidation reaction, we further characterized the photocurrent density in the presence of a hole scavenger with the Na_2_SO_3_, where we can assume that the transported charge carriers at the surface of photoanode are fully consumed by the sacrificing oxidation reaction of the hole scavenger thereby we can exclude the surface catalytic kinetics parameter from the PEC response and comprehend the light harvesting and charge transport performance in the photoanodes. As shown in Figure S19, the Ni_2_P/BVO photoanode achieved a higher photocurrent density of 4.21 mA cm^−^^2^ at 1.23 V_RHE_ compared to 3.67 mA cm^−^^2^ for the BVO photoanode, whereas the same onset potential value, ca. 0.38 V_RHE_ was observed for both photoanodes in the presence of the hole scavenger. While the onset potential without the scavenger (Figure 5d) was significantly reduced from 0.69 V_RHE_ for BVO to 0.49 V_RHE_ for Ni_2_P/BVO, demonstrating the boosted charge kinetics for OER by the presence of Ni_2_P. Especially, the onset potential of NiFeO_x_/Ni_2_P/BVO was dramatically shifted toward a lower region in both with and without a hole scavenger (0.22 V_RHE_ and 0.24 V_RHE_), indicating the improved charge transport efficiency via introduction of NiFeO_x_ overlayer. Meanwhile, there is no change of onset potential between NiFeO_x_/Ni_2_P/BVO and NiFeO_x_/BVO, demonstrating the maximized charge transport performance in the bulk region [33]. To investigate the catalytic activity of water oxidation reaction without illumination, dark current density versus potential curves were characterized (Figure 5e and Figure S20), in which the onset potential value and the overpotential parameter at 1 mA cm^−2^ were also significantly decreased from 2.26 V_RHE_ and 2.62 V_RHE_ for BVO to 2.08 V_RHE_ and 2.32 V_RHE_ for Ni_2_P/BVO, respectively. These decreases in both the onset potential and the overpotential indicate that the water oxidation reaction activity of BVO was significantly enhanced by introducing Ni_2_P NPs. Likewise, the same trend was observed for onset potential and overpotential values of NiFeO_x_/Ni_2_P/BVO (1.79 V_RHE_ and 1.95 V_RHE_, respectively) and NiFeO_x_/BVO (1.8 V_RHE_ and 1.99 V_RHE_, respectively), which implies that the water oxidation reaction performance was still enhanced by Ni_2_P after introduction of NiFeO_x_ overlayer. However, Ni_2_P NPs on the CC substrate had no catalytic activity for water oxidation reaction in the same condition (Figure S21). It can be inferred that the active site of Ni_2_P/BVO is not Ni_2_P. This is consistent with our theoretical prediction from DFT calculations of the electrocatalytic OER energy diagram of Ni_2_P (Figure S2), where Ni_2_P has a large energy barrier of 5.11 eV in the rate‐determining step (*OOH to O_2_(g)) with a bias U = 1.23 V. Consequently, we can assume that the active site on the photoanode is the BVO surface at the Ni_2_P/BVO heterojunction which mainly contributes to the PEC water oxidation. Based on the LSV curves of BVO and Ni_2_P/BVO in both KPi (J_water_) aqueous electrolyte and hole scavenger into the electrolyte (J_scavenger_), the efficiency of charge transfer on the surface (**η**_**transfer**_ = $\frac{J_{\mathrm{water}}}{J_{scavenger}}$ × 100 %) were calculated to investigate the surface catalytic mechanism of the Ni_2_P/BVO shown in Figure 5f and Figure S22. The η_transfer_ values of BVO and Ni_2_P/BVO at 1.23 V_RHE_ were 44.02% and 87.49% respectively, which shows 2‐times higher η_transfer_ after the decoration of Ni_2_P NPs on BVO. In the case of NiFeO_x_/Ni_2_P/BVO and NiFeO_x_/BVO, the η_transfer_ values were 81.3% and 93.9% at 1.23 V_RHE_, respectively, presenting similar aspects with Ni_2_P/BVO and BVO. This significant improved η_transfer_ of NiFeO_x_/Ni_2_P/BVO and Ni_2_P/BVO compared to NiFeO_x_/BVO and BVO demonstrate that the introduction of Ni_2_P on photoanode dramatically boosts surface catalytic activity of NiFeO_x_/Ni_2_P/BVO and Ni_2_P/BVO photoanode. On the other hand, based on the absorption photocurrent density (J_abs_) estimated using the standard solar spectrum and the measured absorbance of the photoelectrodes shown in Figure S23 and J_scavenger_, the charge transport efficiency in bulk was calculated (**η**_**transport**_ = $\frac{J_{scavenger}}{J_{\mathrm{abs}}}$ × 100 %) of Ni_2_P/BVO and pristine BVO had no significant difference at 1.23 V_RHE_ (76.07%), pointing out that the Ni_2_P nanocrystals have a negligible effect on the bulk charge transport of BVO photoanode (Figure S24). Meanwhile, after the introduction of NiFeO_x_ overlayer, η_transport_ efficiency in bulk region of NiFeO_x_/Ni_2_P/BVO and NiFeO_x_/BVO were enhanced (95.1% and 90.1%, respectively), further confirming the abovementioned trend of onset potential comparison between with and without NiFeO_x_ overlayer. The introduction of the NiFeO_x_ overlayer enhances the charge transport efficiency, which synergistically couples with the improved charge transfer efficiency induced by Ni_2_P, enabling the system to achieve a high photocurrent density of 5.49 mA cm^−2^. According to DFT calculations in Figure 1, introducing Ni_2_P NPs may negatively impact hole transport, leading to potential deterioration in performance. However, in this case, the surface total energy barrier was reduced, and the hole carrier transport factor was not dominant because the surface Fermi level of BVO was properly tuned via the introduction of Ni_2_P NPs, thereby improving the PEC performance of the photoanode. In Figure 5g and Figure S25, the wavelength dependent external quantum efficiency (EQE) spectra were obtained from the incident photo‐to‐current conversion efficiency (IPCE) measurement. It demonstrates that the outstanding PEC response of NiFeO_x_/Ni_2_P/BVO and Ni_2_P/BVO compared to NiFeO_x_/BVO and BVO over the entire wavelength range from 350 to 500 nm indicating the ameliorated PEC OER performance by decoration of Ni_2_P. The values of the charge transfer resistance and charge transport resistance were obtained by electrochemical impedance spectroscopy (EIS) to validate the variation in charge transfer/transport efficiency (Figure 5h and Figure S26). The fitting results summarized in Table S1 show that the depositing Ni_2_P nanocrystals on the BVO remarkably decreased charge transfer resistance (R_ct_) from 362.7 Ω to 108.1 Ω. In contrast, charge transport resistance (R_tr_) of photoanodes only had a slight change, with 474.0 Ω and 469.3 Ω for BVO and Ni_2_P/BVO, respectively indicating the minor effects for charge transport in bulk region, validating the similar η_transport_ of Ni_2_P/BVO with BVO as shown in Figure S24. Especially, overlaid NiFeO_x_ for Ni_2_P/BVO and BVO induced the lower R_ct_ (90.05 Ω for NiFeO_x_/Ni_2_P/BVO and 112.8 Ω for NiFeO_x_/BVO) and R_tr_ (220.6 Ω for NiFeO_x_/Ni_2_P/BVO and 397.1 Ω for NiFeO_x_/BVO), which indicates that NiFeO_x_ can boost the charge kinetics in perspective of transfer resistance and transport resistance simultaneously. Intensity‐modulated photocurrent spectroscopy (IMPS) was measured to analyze the surface kinetics in terms of charge transfer (k_ct_). IMPS plots of various photoanodes are plotted in Figure S27. At a potential of 1.23 V_RHE_, recombination semicircle (upper semicircle) disappeared from all the photoanodes, which indicates that almost generated holes reach the surface of BVO based electrodes for participating catalytic reaction due to sufficient potential. k_ct_ of Ni_2_P/BVO was 1.54‐fold higher than that of BVO due to the favoring the catalytic reaction. Furthermore, 4.82‐fold increase of k_ct_ for NiFeO_x_/Ni_2_P/BVO was presented after incorporation of NiFeO_x_ overlayer on the surface of Ni_2_P/BVO, which demonstrates that NiFeO_x_ layer serves as OER catalyst at potential of 1.23 V_RHE_ [55]. The donor density and flat band potential (V_fb_) were examined at a frequency of 1000 Hz using Mott‐Schottky analysis are shown in Figure S28 and summarized in Tables S2 and S3. In non‐illuminated conditions, the Ni_2_P/BVO photoanode exhibited a much smaller slope of the Mott–Schottky than pristine BVO (Figure S28a), suggesting a 4.38‐fold higher donor density in Ni_2_P/BVO compared to that in BVO. This demonstrates that the Ni_2_P NPs could effectively passivate surface states, leading to accelerated charge carrier transfer [9]. Likewise, in the case of NiFeO_x_/Ni_2_P/BVO and NiFeO_x_/BVO, the donor density of NiFeO_x_/Ni_2_P/BVO was presented higher than that of NiFeO_x_/BVO, which indicates that Ni_2_P still leads the enhanced charge carrier transfer after introduction of NiFeO_x_ on BVO based photoanodes. V_fb_, obtained from the x‐axis intercept of the Mott‐Schottky curves, showed a anodic shift from 0.248 V_RHE_ for the BVO photoanode to 0.263 V_RHE_ for Ni_2_P/BVO and also from 0.218 V_RHE_ for the NiFeO_x_/BVO to 0.233 V_RHE_ for NiFeO_x_/Ni_2_P/BVO, indicating flattened band bending after introduction of Ni_2_P on the surface of BVO, which is consistent with abovementioned our calculation results [56]. It demonstrates that the incorporation of Ni_2_P on BVO induced an upward modulation of the Fermi level to a higher energy level. Similarly, the same trend was observed under illumination conditions. Upon the introduction of Ni_2_P, the flat band potentials of BVO and NiFeO_x_/BVO (0.225 V_RHE_ and 0.234 V_RHE_, respectively) were upshifted to 0.240 V_RHE_ (Ni_2_P/BVO) and 0.245 V_RHE_ (NiFeO_x_/Ni_2_P/BVO). This indicates that the Ni_2_P‐induced Fermi level upshift is maintained even under illumination conditions. In addition to photocatalytic performance, stability is a vitally important criterion of a photoelectrode for practical application. Long‐term stability tests were conducted on BVO‐based photoanodes under continuous illumination at potentials corresponding to the maximum applied bias photo‐to‐current efficiency (ABPE) as shown in Figure S29. In Figure 5i and Figure S30, the photocurrent density of pristine BVO significantly decreased from 0.72 to 0.14 mA cm^−2^ after 10 h of operation. Otherwise, the current density of Ni_2_P/BVO photoanode exhibited almost no decrement for 10 h operation from 2.02 to 1.92 mA cm^−2^. Especially, in the case of NiFeO_x_/Ni_2_P/BVO, the photocurrent density of 3.43/ mA cm^−2^ was achieved after 10 h of operation, maintaining 97.9% efficiency compared with the initial photocurrent, which demonstrates the outstanding stability of the NiFeO_x_/Ni_2_P/BVO photoanode. Furthermore, Ni 2p and P 2p XPS spectra of Ni2P/BVO and BVO photoanode after 10 h long‐term stability test (Figure S31a,b) did not show a significant change, further confirming the stability of Ni_2_P/BVO photoanode. Meanwhile, Bi 4f and V 2p of Ni_2_P/BVO and BVO have slightly shifted toward higher binding energy after 10 h long‐term stability test (Figure S31c,d). This indicates both the excellent long‐term stability of the material and that the active sites are located on the BVO surface. We further compared the performance of our NiFeO_x_/Ni_2_P/BVO with that of other recently reported BVO‐based photoelectrodes (Figure S32 and Table S4). The results reveal that our NiFeOx/Ni_2_P/BVO exhibits comparable or superior performance relative to the pioneering BVO‐based photoelectrodes.

Considering Ni_2_P as an effective electrocatalyst for HER, as demonstrated in Figure S33 [57], a solar‐assisted overall water splitting with 2‐electrode configuration using Ni_2_P/CC as cathode and Ni_2_P/BVO as photoanode was employed, as shown in Figure 6a. In comparison purpose, the overall water splitting cell composed of pristine BVO/FTO||CC was also evaluated. The LSV curves in Figure 6b show that the Ni_2_P/BVO/FTO||Ni_2_P/CC cell achieved a photocurrent density of 2.61 mA cm^−2^ at the cell voltage of 1.23 V, which was much greater than that of the BVO/FTO||CC cell (1.03 mA cm^−2^). Additionally, a pronounced cathodic shift of the onset voltage from 0.43 V for BVO to 0.34 V for Ni_2_P/BVO was observed after deposition of Ni_2_P NPs on the CC electrodes, which exhibits the promoted surface catalytic kinetics on both OER and HER in the Ni_2_P‐integrated cell. Long‐term stability of Ni_2_P/BVO/FTO||Ni_2_P/CC cell in Figure S34 reveals the high durability of Ni_2_P‐based 2 electrode system for solar‐assisted overall water splitting. The quantities of H_2_ and O_2_ versus time generated from PEC overall water splitting over Ni_2_P/BVO/FTO||Ni_2_P/CC cell were measured by GC system in an airtight single cell with the bias of 1.23 V under simulated solar illumination. As shown in Figure 6c, after 2 h of operation, the amount of H_2_ and O_2_ linearly increased to 84.1 and 44.1 µmol cm^−2^, respectively, which indicates that the stoichiometric produced gas ratio of as 2:1 evidenced no side reaction except water splitting reaction.

The optical properties of photoanodes were analyzed by UV–vis spectra shown in Figure S23. All the films exhibit an absorption edge at approximately 500 nm, which is characteristic of BVO and consistent with the previous reports [35, 38]. In the case of NiFeO_x_/BVO, there is no significant difference from BVO in the overall wavelength region, which indicates that the NiFeO_x_ overlayer with about 10 nm of thickness has no effect for optical properties. In the 300–450 nm wavelength range, the NiFeO_x_/Ni_2_P/BVO and Ni_2_P/BVO photoanode show a slight decrease in light absorption compared to NiFeO_x_/BVO and BVO, attributed to the light shielding effect caused by Ni_2_P NPs loaded on the BVO surface. However, this effect was limited due to the small size and uniform dispersion with low coverage of Ni_2_P NPs [53, 58, 59]. Conversely, in the 500–700 nm range, the absorption curve of NiFeO_x_/Ni_2_P/BVO and Ni_2_P/BVO are much higher than that of pristine BVO, owing to the intrinsic absorption of metallic semiconductor Ni_2_P NPs [60‐61]. The optical bandgaps of NiFeO_x_/Ni_2_P/BVO, NiFeO_x_/BVO, Ni_2_P/BVO, and BVO films were calculated using Tauc plots to be 2.52 eV, respectively (Figure S35). Obviously, the introduction of Ni_2_P did not significantly change the bandgap between samples. XPS valence band spectra (Figure S36) and UPS (Figure S37) were also used to clarify the effect of modification Ni_2_P on surface band energy structure. The cutoff energy of BVO measured by UPS increased from 16.32 eV before Ni_2_P incorporation to 16.46 eV after incorporation, indicating that the work function was reduced from 4.90 to 4.76 eV, corresponding to an upward shift of the Fermi level. Similarly, after the introduction of NiFeO_x_, the cutoff energy increased from 16.05 to 16.13 eV, yielding work function values of 5.17 eV for NiFeO_x_/BVO and 5.09 eV for NiFeO_x_/Ni_2_P/BVO. This consistent trend confirms that the Ni_2_P‐induced Fermi level upshift is preserved even after the deposition of the NiFeO_x_ layer, indicating that the Fermi level modulation effect of Ni_2_P remains intact. Combining the UV–vis, XPS valence band spectra, and UPS spectra, the schematic illustration of a detailed band diagram is shown in Figure 6d and Figure S38, both conduction band minimum (CBM) and VBM of Ni_2_P/BVO were shifted toward the vacuum energy level compared to pristine BVO. This CBM and VBM upshifts are well consistent with our theoretical predictions: as Ni_2_P/BVO forms an Ohmic junction, interface space charge on BVO in the heterojunction becomes negative, and Ni_2_P surface is charged positive. Switching the positive space charge (typical bare n‐type semiconductor surfaces) to negative charge by heterojunction formations induce upshifted CBM and VBM due to the electrostatic potential difference by the interface dipole [62].

## 3 Conclusions

In summary, we demonstrated the possibility of rational selection and optimizations of metallic NPs deposition for PEC photoanode surface, where surface Fermi modulation plays the crucial role in the reaction activity. Utilizing the Ni_2_P/BVO heterojunction photoanode as a model system, our computational results reveal that depositing Ni_2_P NPs can pin the surface Fermi level higher, which is beneficial to PEC water splitting on the BVO surface by lowering the surface reaction energy barrier. Although the downward surface band bending of BVO in the Ohmic junction with Ni_2_P reduces hole carrier delivery, this drawback can be compensated with higher surface reaction rate when the coverage of Ni_2_P NPs is in the optimal range (6%–11%). As the facilitator, the rational nano‐positioning of spherical Ni_2_P NPs on the BVO photoanode, with the optimal coverage of Ni_2_P at 9.4%, promoted the PEC activities with enhanced OER kinetics of BVO, resulting in the significantly enhanced photocurrent density of 5.49 mA cm^−2^ at 1.23 V_RHE_ under 1 sun illumination after introduction of NiFeO_x_ overlayer, which was 3.4‐fold higher than that of the pristine BVO and outstandingly higher than Ni_2_P/BVO photoanodes with various coverage of Ni_2_P from 3.3% to 17.9%. Additionally, due to the considerable hydrogen evolution reaction (HER) kinetics of Ni_2_P, the incorporation of Ni_2_P NPs on BVO for photoanodic OER and on the CC substrate for electrocatalytic HER facilitates the solar‐assisted overall water splitting to produce H_2_ with the production rate of 42.1 µmol cm^−2 ^h^−1^ in the 2‐electrode configuration cell composed of Ni_2_P/BVO/FTO||Ni_2_P/CC. Our findings report solid evidence that Fermi level pinning effects of the BVO photoanode can boost the PEC water splitting and suggest that the rational regulation of metallic NPs on the surface of the photoanode can facilitate surface reaction kinetics, providing a new perspective for designing high‐efficiency photoanodes in solar water‐splitting systems.

## 4 Experimental Section

### 4.1 Preparation of BVO Photoanode

The nanoporous BVO photoanodes were fabricated according to the previous report [63]. Initially, fluorine doped SnO_2_‐coated (FTO) glass substrates were spin‐coated with a thin SnO_2_ layer and annealing at 550°C for 2 h before BiOI growth. The SnO_2_ precursor was prepared by using 108 mM tin (II) chloride pentahydrate (SnCl_2_·5H_2_O, Sigma Aldrich) dissolved in a mixed solvent of 2‐methoxy ethanol (Sigma Aldrich) and acetylacetone (Sigma Aldrich) with 1:0.02 volume ratio, followed by magnetic stirring at room temperature for 24 h. Then, BiOI films were grown on FTO substrates via the solvothermal method. In a typical process, a BiOI‐precursor solution consisting of 0.545 g of bismuth nitrate pentahydrate (Bi(NO_3_)_3_·5H_2_O, Sigma Aldrich) and 0.187 g of potassium iodide (KI, Sigma Aldrich) in a 20 mL mixed solvent of ethylene glycol (Sigma Aldrich) and ethanol (Sigma Aldrich) with 5:3 volume ratio was transferred to a 50 mL Teflon autoclave holding FTO glass substrates for the solvothermal reaction at 140°C for 3 h. When the reaction was over, the resultant BiOI films were washed with ethanol and then dried with an N_2_ stream. After that, 90 µL of a 0.5 m vanadyl acetylacetonate (VO(acac)_2_, Sigma Aldrich) solution in dimethyl sulfoxide (Acros) was dropped on the BiOI film surface (1.5 × 2 cm^2^). These samples were then calcined at 400°C for 2 h with a ramping rate of 2°C min^−1^ to promote the conversion of BiOI to BVO. Afterwards, the BVO film was immersed in a 1 m NaOH solution for 20 min with gentle stirring to eliminate excess V_2_O_5._ The resultant yellow BVO films were rinsed with deionized water and dried in the air.

### 4.2 Preparation of Ni_2_P Nanoparticles

Ni_2_P nanocrystals were prepared by a hot‐injection method based on a previously described procedure [64]. Briefly, 1 g of nickel acetate tetrahydrate (Ni(ac)_2_·4H_2_O, Sigma Aldrich), 18 mL of 1‐octadecene (Sigma Aldrich), 25.6 mL of oleylamine (Sigma Aldrich), and 8 mL of tri‐n‐octylphosphine (Acros) were added a three‐necked flask (100 mL) fitted with a condenser and magnetic stirring. The reaction mixture was stirred moderately and heated by a heating mantle at 120°C for 1 h in constant evacuation mode to remove moisture and other low‐boiling impurities. The solution was then degassed under N_2_, heated to 320°C at a ramp rate of 5°C min^−1,^ and then kept at this temperature for 2 h. After the reaction was completed, the solution was naturally cooled down to room temperature. Then, the resulting suspension was purified to completely remove the excess ligands and organic solvent by using 1:3 (v: v) hexanes: ethanol, the washing procedure was repeated at least three times. The collected precipitate was finally dried in vacuum oven.

### 4.3 Preparation of Ni_2_P/BVO Photoanode

The Ni_2_P/BVO photoanode was fabricated by loading Ni_2_P NPs onto the BVO surface using a simple drop‐casting technique. First, Ni_2_P NPs were dispersed in chloroform (0.5 mg mL^−1^) and sonicated for 1 h to achieve a uniform ink. Then, 20 µL of the dispersed solution was drop‐casted on the BVO photoanode surface and allowed to dry naturally. This dropping and drying process was repeated until an appropriate amount of Ni_2_P was obtained on the surface of the BVO photoanode. Finally, the as‐prepared Ni_2_P/BVO was dried in a vacuum chamber (∼10^−2^ Torr) overnight at room temperature. The different coverages of Ni_2_P on the BVO surface were adjusted according to the amount of Ni_2_P loaded on the surface of BVO, and the samples were named Ni_2_P‐x%/BVO (x% as the coverage of Ni_2_P NPs on BVO). For convenience, Ni_2_P/BVO stands for Ni_2_P‐9.4%/BVO if no specifications are given.

### 4.4 Preparation of NiFeO_x_/Ni_2_P/BVO

The NiFeO_x_ layers were deposited onto BVO and Ni_2_P/BVO electrodes via the photo‐electrodeposition based on a previous report [33] to obtain NiFeO_x_/BVO and NiFeO_x_/Ni_2_P/BVO photoanodes, respectively. Specifically, under AM 1.5G illumination, chronoamperometry (CA) was performed at 0.6 V versus Ag/AgCl for 5 min in an electrolyte containing 0.4 m FeSO_4_ and 0.04 m NiSO_4_.

### 4.5 Material Characterization

The morphology of photoanodes was measured using Field‐emission scanning electron microscopy (FESEM, JSM‐IT800). High‐resolution transmission electron microscopy (HR‐TEM), elemental mapping, and line profiling were conducted on a JEM ARM 200F transmission electron microscope. The crystalline structures were explored by X‐ray diffraction analysis (XRD, Bruker D8 Discover) at 40 kV. X‐ray photoelectron spectroscopy (XPS) analysis was carried out using an ESCALAB 250 spectrometer. Ultraviolet photoelectron spectroscopy (UPS) was conducted by using a NEXSA G2 spectrometer. The optical properties were identified by a UV–vis spectrometer (SHIMADZU UV‐2600).

### 4.6 Photoelectrochemical Characterization

PEC photoanode measurements were carried out using CHI660B and Gamry Ref 600+ potentiostats. PEC devices were evaluated in 0.1 M potassium phosphate (KPi) aqueous solution (pH 7) employing a conventional three‐electrode configuration: counter electrode (Pt sheet), working electrode (BVO‐based photoanodes), and reference electrode (Ag/AgCl, 3 M KCl). A 150 W Xenon lamp (PEC‐L01, PECCELL, Japan) simulated an AM 1.5 G sunlight (100 mW cm^−2^), and the illumination was applied from the back side of the photoanodes. The photocurrent was determined through linear sweep voltammetry (LSV) at a scan rate of 10 mV s^−1^. 0.5 M sodium sulfite (Na_2_SO_3_) was introduced into the KPi electrolyte solution as a hole scavenger for assuming that the transported charge carriers at the surface of photoanode are fully consumed by the sacrificing oxidation reaction of the hole scavenger. Incident photon to current conversion efficiency (IPCE) was investigated by using Ivium Technologies potentiostat with 150 W Xe lamp (ABET Technologies) as light source and monochromator (MonoRa150i, DONGWOOOPTRON Corp.). The electrochemical impedance spectroscopy (EIS) was conducted at 1.23 V_RHE_ with an amplitude of 5 mV, ranging from 1 MHz to 0.1 Hz, under AM 1.5 G irradiation. Intensity‐modulated photocurrent spectroscopy (IMPS) was conducted using an electrochemical workstation (Zennium, Zahner, Germany) and potentiostat (PP211, Zahner, Germany) under the sweeping frequency from 100 kHz to 0.1 Hz. Mott‐Schottky measurements were recorded at a frequency of 1000 Hz with an amplitude of 10 mV under dark conditions. All potentials were recorded versus Ag/AgCl 3 M KCl reference and then converted against an RHE using the Nernst relationship ERHE  =  EAg/AgCl  +  0.059*pH +  E^0^Ag/AgCl. The overall water splitting reaction was measured using a two‐electrode configuration, where Ni_2_P/BVO served as a photoanode, and a carbon cloth (CC) substrate coated with Ni_2_P NPs acted as a cathode with a single area of 1 x 1 cm^2^. For preparation of Ni_2_P electrode, 5 mg of Ni_2_P catalyst was dispersed in 1 mL of 1:3 (v: v) DI water: ethanol along with 15 µL of Nafion solution, and ultrasonicated for 1 h to achieve a homogeneous mixture. Then, 80 µL of the resulting mixture was dropped onto the CC and dried in a vacuum oven to evaporate the solvent completely. The experiments were conducted in a 0.1 M KPi electrolyte, and using a light source same as the one used in the above PEC measurements. A tightly sealed gas photoelectrochemical cell, integrated with a gas chromatography (YL6500 GC, Youngin Chromas), was utilized to analyze the evolution of the reaction's generated H_2_ and O_2_ gases. The potential was applied at the cell voltage of 1.23 V, and gas samples were collected every 30 min for analysis.

### 4.7 Density Functional Theory Calculations

DFT calculations were carried out using Vienna ab initio simulation package (VASP) with the projector augmented were method (PAW) to describe the electron‐ion interactions [65, 66, 67]. The general gradient approximation (GGA) with the Perdew‐Burke‐Ernzerhof (PBE) exchange correlation functional was employed [68, 69]. To improve the description of 3d electrons of vanadium, GGA+U method within Dudarev's approach was employed [70]. The Gamma‐centered k‐point sampling of Brillouin zone was obtained using a 2 x 2 x 1 supercell by Monkhorst‐Pack for the structure relaxation and a kinetic energy cut‐off of 400 eV was used [71]. The energy convergence criteria in the self‐consistent field were set to 10^−6^ eV and all geometry structures were fully relaxed until Hellman‐Feynman forces achieved a range of 0.1 eV ${Å}^{-1}$.

## Author Contributions

P.T.P., S.J., and U.B. contributed to this work equally. P.T.P. carried out the synthesis, methodology, experiment operation and writing – original draft. S.J. conducted the DFT theoretical calculations. U.B. conducted the material characterizations, data analysis, methodology and writing – review and editing. Y.L. carried out data analysis, validation, and data curation. D.N.N. assisted with the material synthesis. W.T.H. conducted the material characterizations, data analysis, and methodology. Y.Y. and J.M. assisted with visualization. K.J., J.Y.L., and C.‐H.C. designed and proposed parts of the experiments and revised the manuscript. T.‐H.K. carried out the HR‐TEM characterizations. J.P. supervised validation, conceptualization, and resources. H.C. supervised the theory model and DFT calculations, conceptualized this work, and conducted writing – review and editing. J.K.K. supervised and conceptualized this work, designed the experiments, and conducted project administration and writing – review, and editing. All authors participated in the discussion and writing of the manuscript.

## Conflicts of Interest

The authors declare no conflicts of interest.

## Supporting information




**Supporting File**: advs75734‐sup‐0001‐SuppMat.docx.

## Data Availability

The data that support the findings of this study are available from the corresponding author upon reasonable request.
