# Supplementary material for: Surface Fermi Level Modulation of Photoanode by Optimized Conducting Nanoparticle Heterointerfaces for Enhanced Photoelectrochemical Water Splitting
Source: Adv Sci (Weinh). 2026 May 19:e75734. Online ahead of print. doi: 10.1002/advs.75734 (PMC13336096; doi:10.1002/advs.75734)
Supplement: Supplementary file 1 — Supporting File: advs75734‐sup‐0001‐SuppMat.docx. [file ADVS-9999-e75734-s001.docx]

**Supporting Information**

**Surface Fermi Level Modulation of Photoanode by Optimized Conducting Nanoparticle Heterointerfaces for Enhanced Photoelectrochemical Water Splitting**

*Phuong Thi Pham^1^, Seulgi Ji^2^, Unbeom Baeck^1^, Yuankai Li^1^, Duong Nguyen Nguyen^3^, Won Tae Hong^1^, Yang You^4^, John Moraros^4^, Kyoungsuk Jin^3^, Jun Young Lee^1^, Chan-Hwa Chung^1^, Tae-Hoon Kim^5^, Jongwook Park^6*^, Heechae Choi^4*^, Jung Kyu Kim^1,7*^*

^1^ School of Chemical Engineering, Sungkyunkwan University (SKKU), 2066, Seobu-ro, Jangan-gu, Suwon 16419, Republic of Korea.

^2^ Institute of Inorganic and Materials Chemistry, University of Cologne, Greinstr. 6, 50939, Cologne, Germany

^3^ Department of Chemistry and Research Institute of Natural Science, Korea University, Seoul, 02841 Republic of Korea

^4^ Advanced Materials Research Center (AMRC) & Department of Chemistry and Materials Science, School of Science, Xi’an Jiaotong-Liverpool University, Suzhou, Jiangsu, 215123, China

^5^ Department of Materials Science and Engineering, Engineering Research Center, Chonnam National University, Gwangju, 61186, Republic of Korea

^6^ Integrated Engineering, Department of Chemical Engineering, Kyung Hee University, Yongin 17104, South Korea.

^7^ SKKU Advanced Institute of Nanotechnology, Sungkyunkwan University (SKKU), Suwon, 16419, Republic of Korea

* Corresponding authors: legkim@skku.edu (J. K. Kim) and heechae.choi@xjtlu.edu.cn (H. Choi) and jongpark@khu.ac.kr (J. Park)

**Experimental**

OER steps on Ni_2_P/BVO considering the intermediates with the thermodynamically favored charge state can be expressed as,

(i) H_2_O (l) → OH^-^ + H^+^ (1)

(ii) OH^-^ → O^2-^ + H^+^ (2)

(iii) O^2-^ + H_2_O (l) → OOH^-^ + H^+^ + 2e^-^ (3)

(iv) OOH^-^ → O_2_ (g) + H^+^ + 2e^-^ (4)

on the bare BVO without illumination,

(i) H_2_O (l) → OH^+^ + H^+^ + 2e^-^ (5)

(ii) OH^+^ → O^+^ + H^+^ + e^-^ (6)

(iii) O^+^ + H_2_O (l) → OOH^+^ + H^+^ + e^-^ (7)

(iv) OOH^+^ → O_2_ (g) + H^+^ (8)

Additionally, the reaction steps of water oxidation on the bare BVO and Ni_2_P/BVO with illumination can be considered as,

(i) H_2_O (l) + 2h^+^→ OH^+^ + H^+^ (9)

(ii) OH^+^ + h^+^→ O^+^ + H^+^ (10)

(iii) O^+^ + H_2_O (l) + h^+^ (l) → OOH^+^ + H^+^ (11)

(iv) OOH^+^ → O_2_ (g) + H^+^ (12)Theory


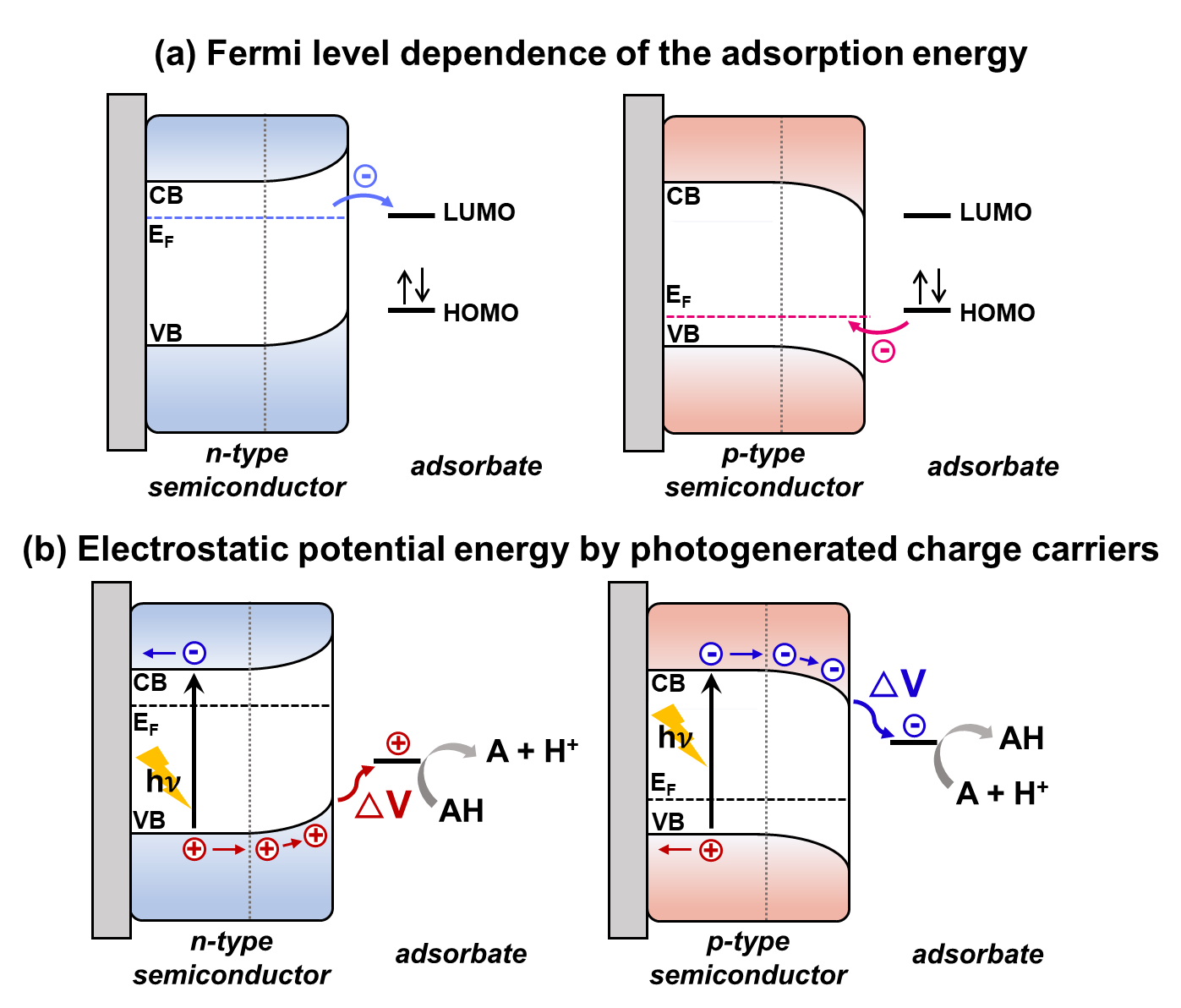


Scheme S1. Energy band diagram of BVO with the schematic explanation of (a) Fermi level dependence of the adsorption energy due to Fermi level variation and (b) how to consider the effect of potential energy change by photogenerated charge carriers on Gibbs free energy calculations.

The Gibbs free energies of reaction intermediates in PEC water oxidation on BVO surface were calculated using the following equation:

$\Delta G_{\mathrm{ads}}=\Delta E+\Delta ZPE+\int C_{p}\mathrm{dT}-T\Delta S+\Delta G_{\mathrm{pH}}+\Delta G_{U}+q\left( \varepsilon_{F}+E_{\mathrm{VBM}} \right)-ne\Delta V$ (1)

where ∆E is the calculated total energy change by adsorption of intermediates, as obtained from DFT calculation. The terms ∆ZPE and ∆S in equation (1) are the changes of zero-point energy and entropy at temperature T, ∆G_pH_ is -k_B_Tln10×pH, k_B_ is the Boltzmann constant, the ∆G_U_ term means the applied bias (*U*) and is expressed as the product of the number of electrons, charge of electron, and bias: –ne*U*. Since the potential energy change by the hole carrier transfer between adsorbates and BVO is determined by the Fermi level position of BVO, the terms to consider the Fermi level dependency of the adsorption energy is included in equation (1). The terms q, ε_F_ and E_VBM_ in equation (1) represent the charge state of the intermediates, Fermi level ranging over the band gap of BVO (0 eV–2.5 eV), and DFT-computed eigenvalue of the valence band maximum (VBM) energy level of BVO, respectively (Scheme 1a). We considered the charge states (q) of intermediates O (q = -2, -1, 0, +1), OH (q = -1, 0, +1), and OOH (q = -1, 0, +1), which possibly be formed due to charge transfer between BVO and the intermediates. It can be assumed that the charge states of reaction intermediates can be determined by the octet rule and the relative positions between the Fermi level of BVO photoanode surface and the highest occupied molecular level (HOMO) or the lowest unoccupied molecular orbital (LUMO) of the intermediates (OH, O, OOH).

In a PEC water oxidation reaction on BVO, the Gibbs free energy of each intermediates become more negative, due to the potential gap between the VBM and the water oxidation reaction (1.23V). In this regard, we added the electrostatic potential energy term (-ne∆V) in equation (1). The ∆V term in equation (1) is the potential difference between the VBM, and the water oxidation potential and n is the number of involved holes in each reaction step, respectively (Scheme 1b). (1) The terms $\Delta$ZPE, $\int C_{p}dT$ and $\Delta S$ in equation (1) indicate the changes of zero-point energy, enthalpic and entropy contribution correction at temperature T, respectively, and can be expressed as:

$\Delta ZPE= \frac{1}{2}\sum\hbar\omega_{i}$ (2)

$\int C_{p}\mathrm{dT}=\sum_{i} \frac{\hbar\omega_{i}}{\exp\left( \frac{\hbar\omega_{i}}{k_{B}T} \right)-1}$ (3)

$\Delta S=k_{B}\sum_{i} [\frac{\hbar\omega_{i}}{\exp\left( \frac{\hbar\omega_{i}}{k_{B}T} \right)-1}-ln(1-exp\left( -\frac{\hbar\omega_{i}}{k_{B}T} \right))]$ (4)

where $\hbar$ and $\omega_{i}$ are the reduced Planck constant and ($\hbar=h/{2\pi}$), and the vibrational frequency eigenvalue of *i*-th mode. (2)

**
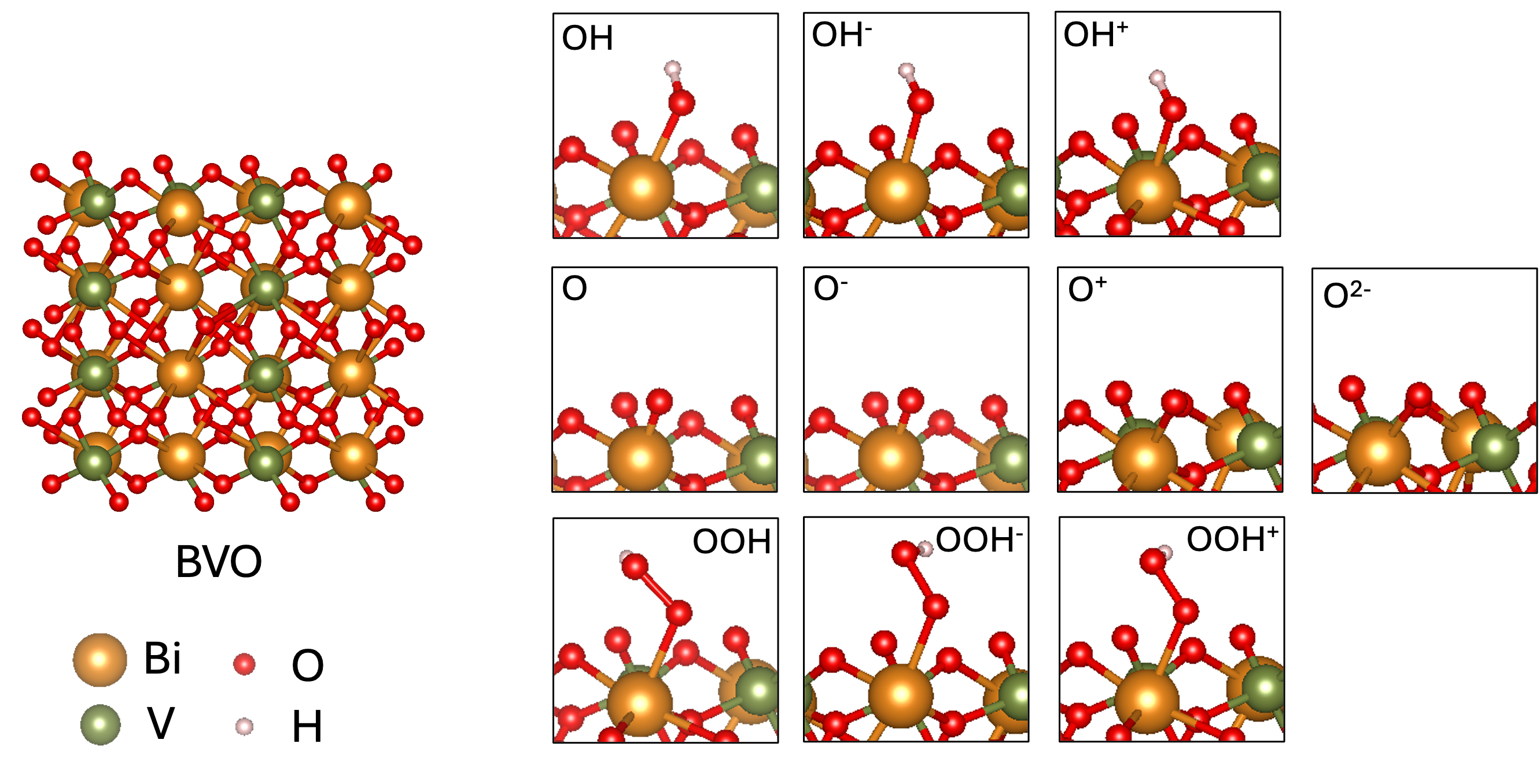
**

**
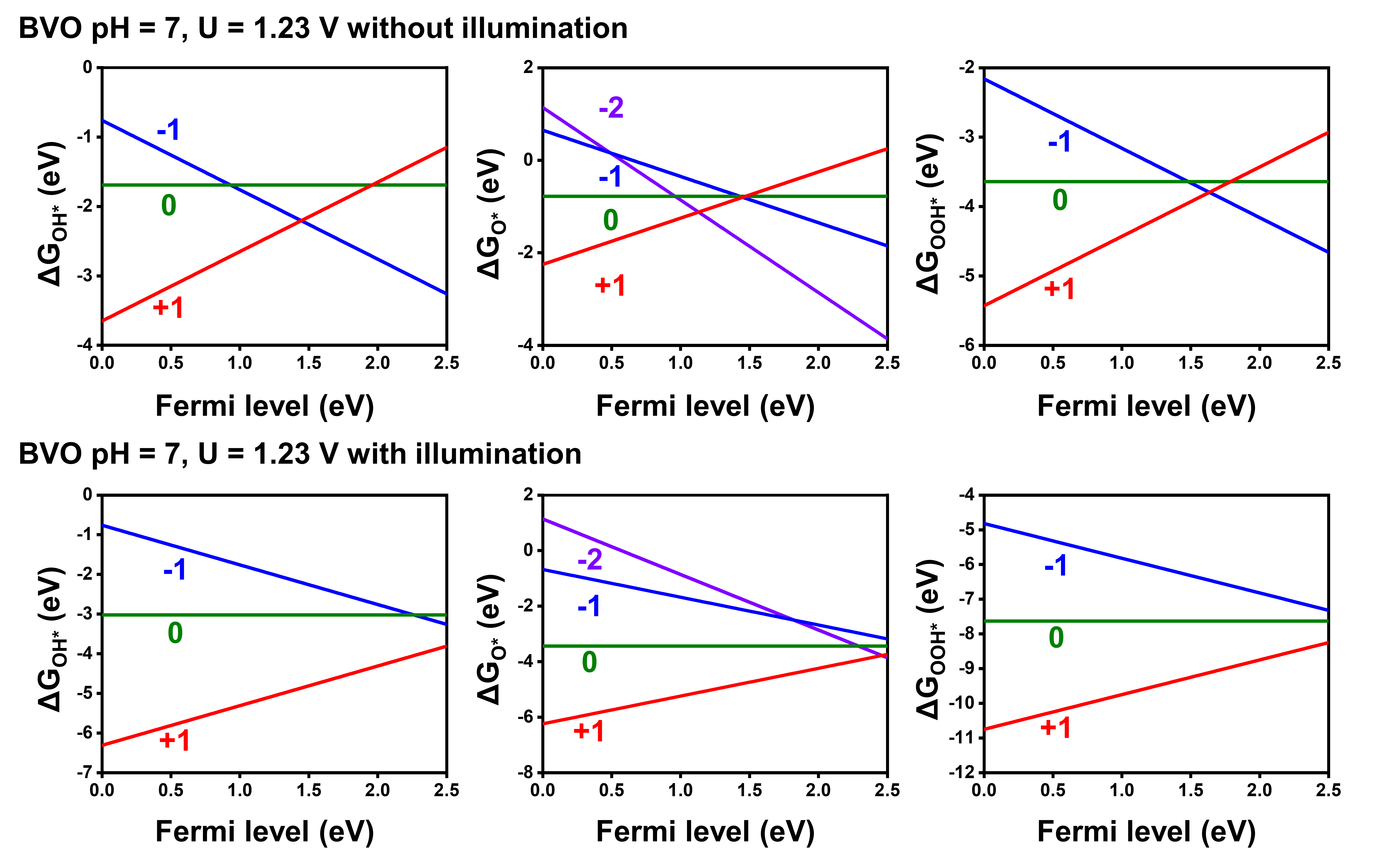
**

**Fig. S1.** (Top) Slab model of BVO and optimized adsorption configurations of OER intermediates on BVO. (Bottom) Calculated adsorption energies of *OH, *O, *OOH on bare BVO with various charge states, as functions of Fermi level without and with illumination at pH = 7 and U = 1.23V.

**
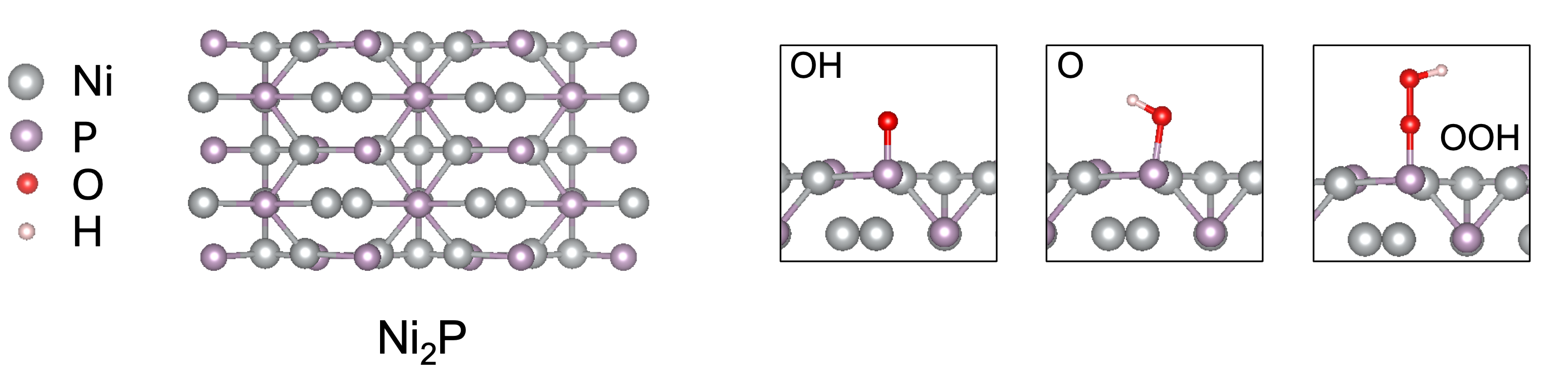
**

**
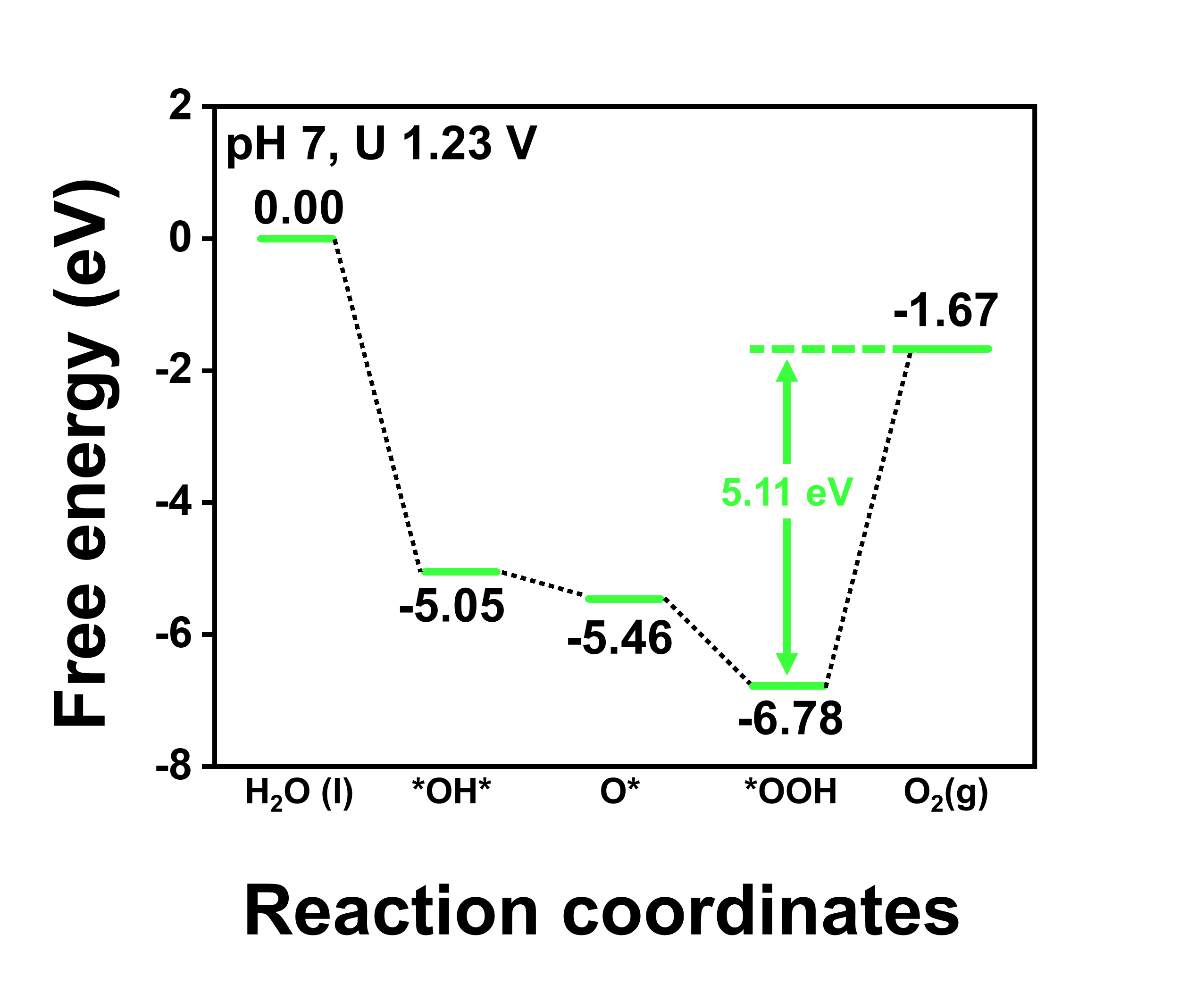
**

**Fig. S2.** (Top) Slab model of Ni_2_P and optimized adsorption configurations of OER intermediates on Ni_2_P (Bottom) the electrocatalytic OER energy diagram on Ni_2_P.

**
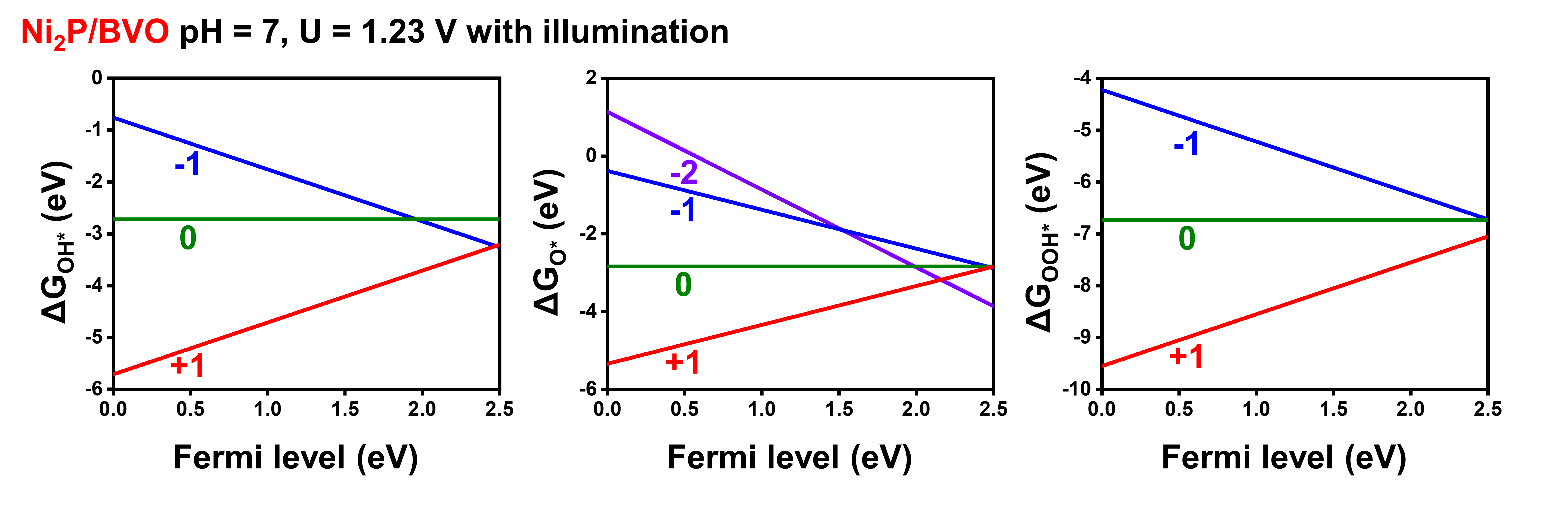
**

**Fig. S3.** The energy band diagram of Ni_2_P/BVO and calculated adsorption energies of *OH, *O, *OOH on Ni_2_P/BVO with various charge states as functions of Fermi level with illumination at pH = 7 and U = 1.23 V.


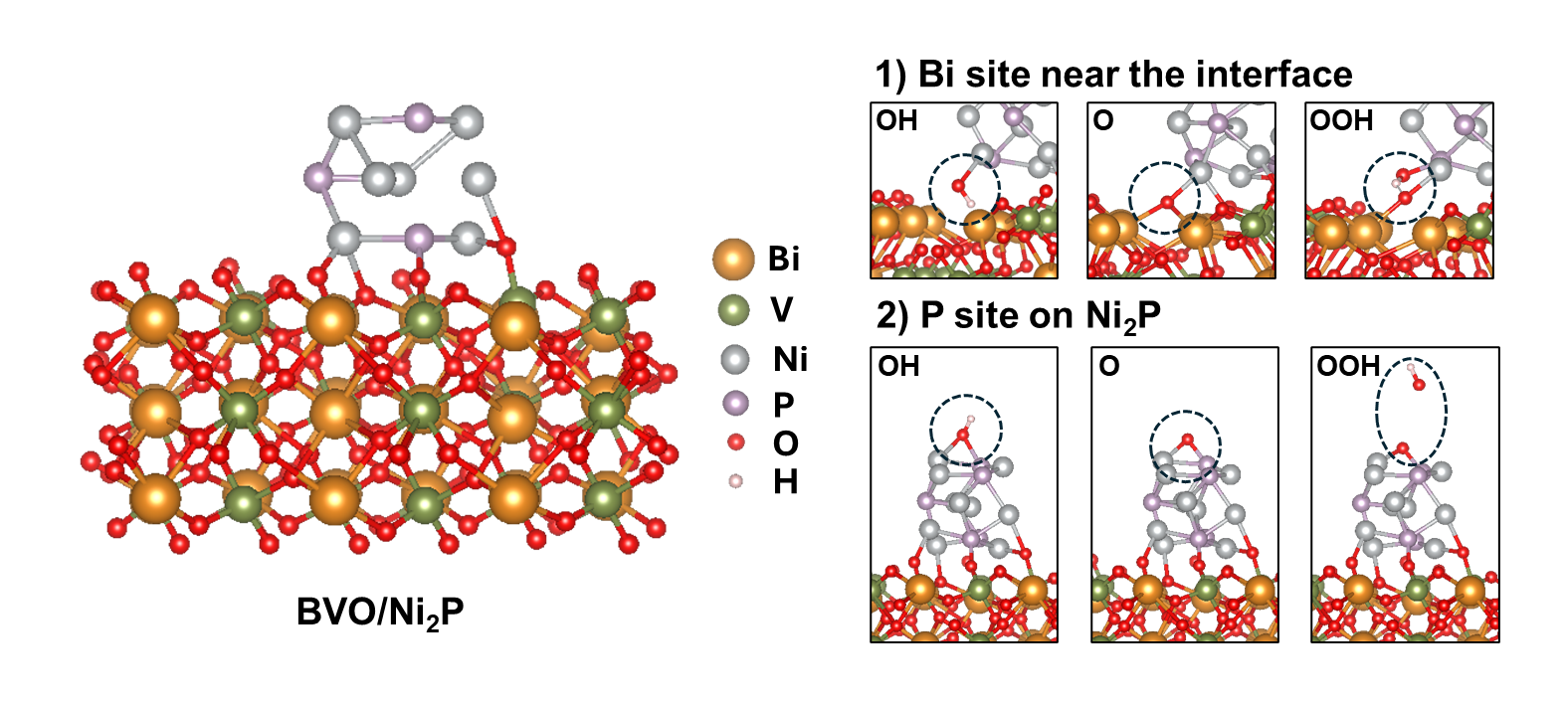


**Fig. S4.** Slab of an explicit Ni_2_P/BVO interfacial model and optimized adsorption configurations.

^
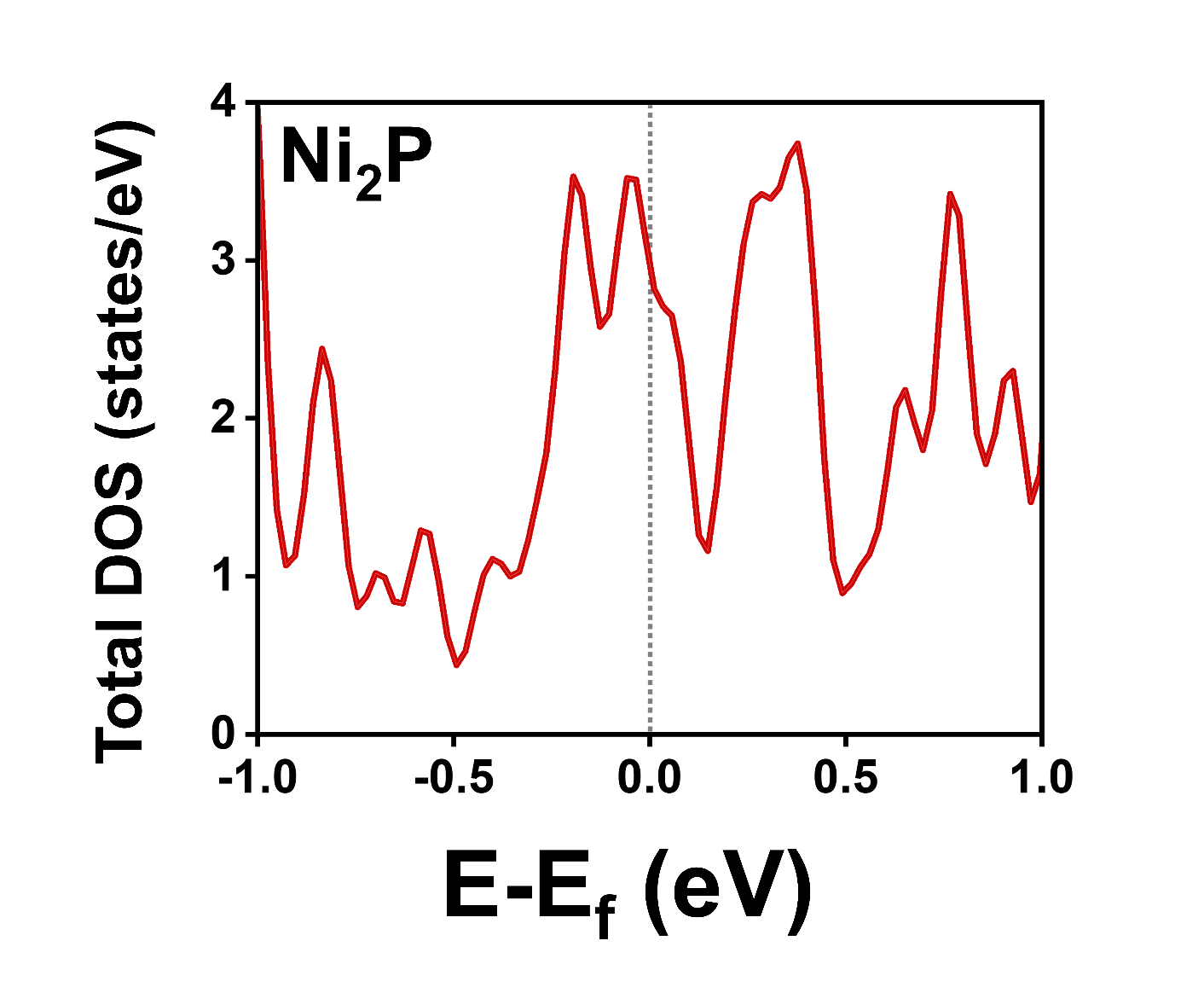
^

**Fig. S5.** Density of states of Ni_2_P

**
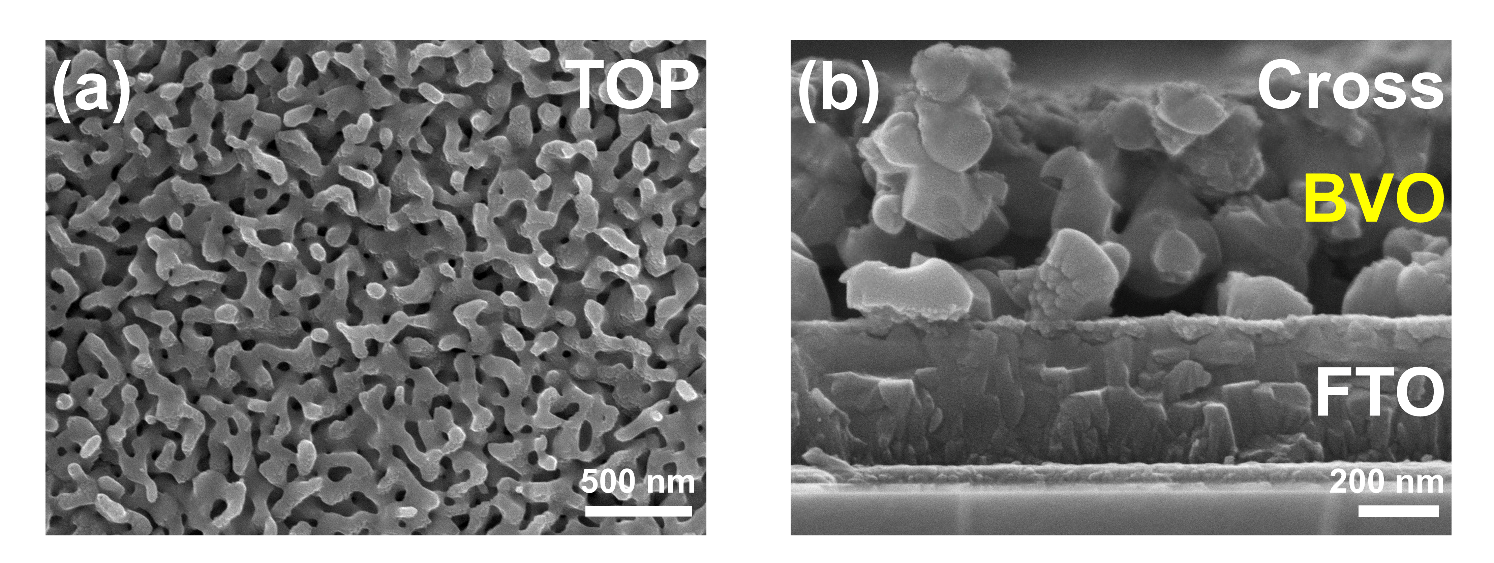
**

**Fig. S6.** (a) Top view, and (b) cross-sectional view SEM images of BVO on the FTO substrate.


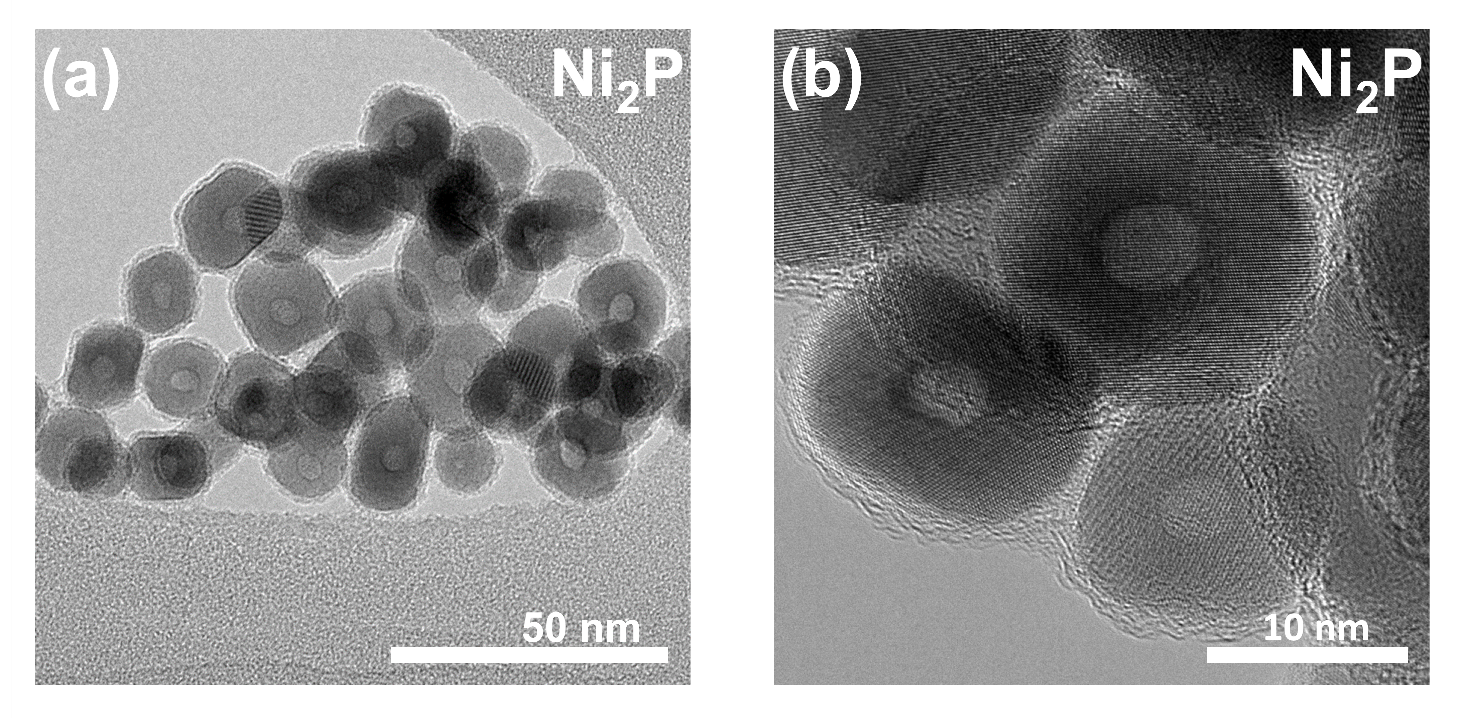


**Fig. S7.** (a) TEM image, (b) HRTEM image of Ni_2_P nanocrystals.


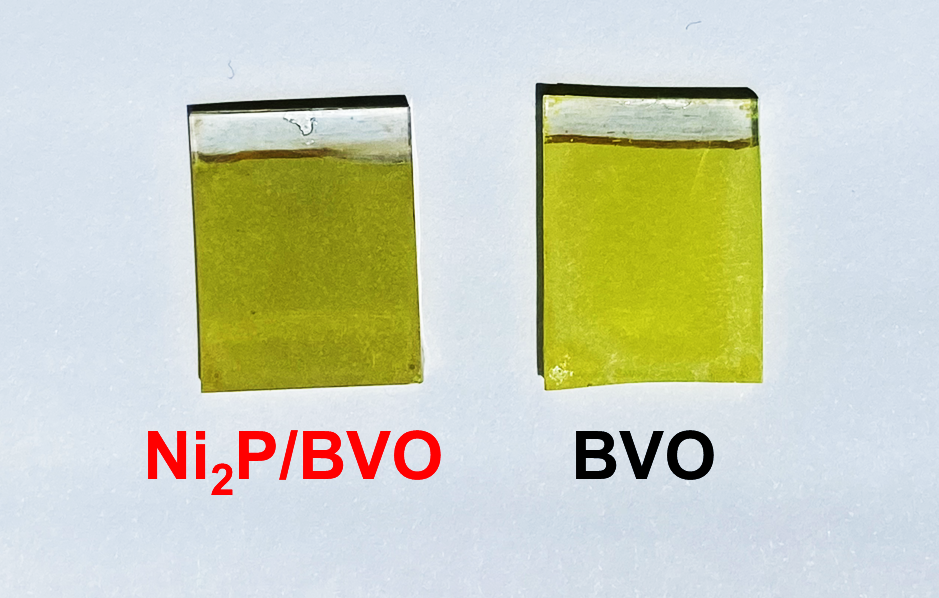


**Fig. S8.**  Photos of the Ni_2_P/BVO and BVO photoanodes.

**
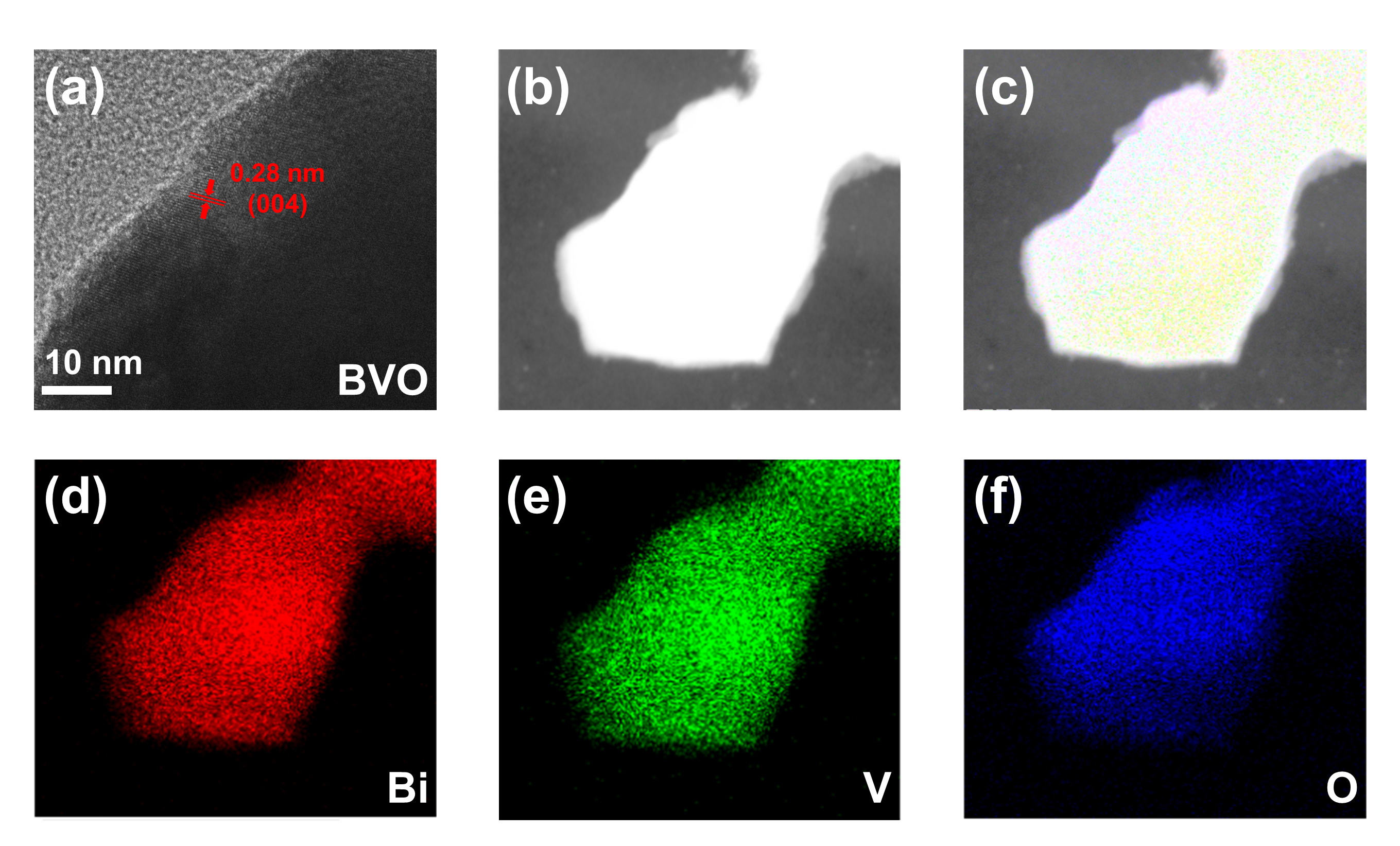
**

**Fig. S9.** (a) TEM image and (b-f) EDS element mapping of BVO.

**
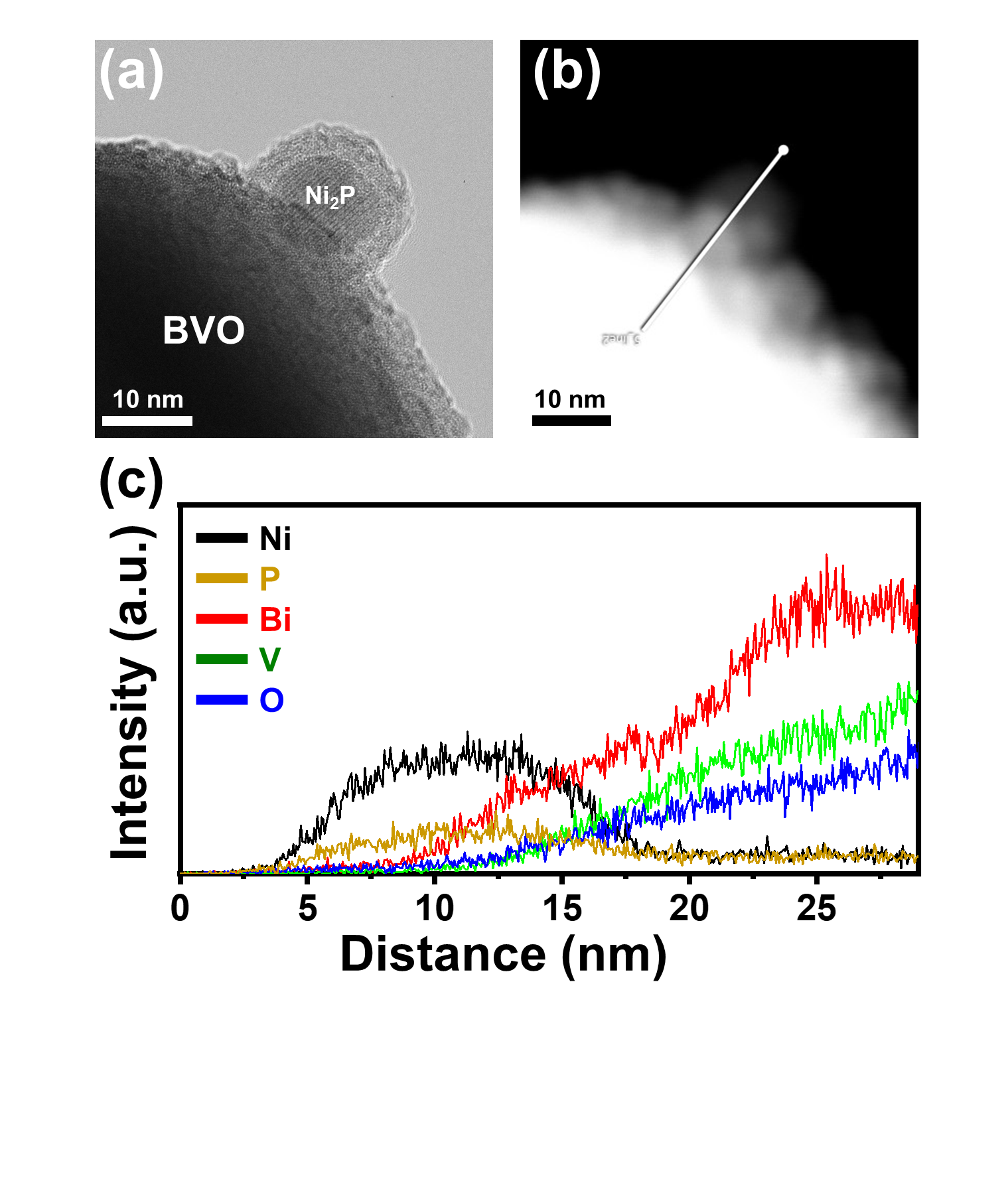
**

**Fig. S10.** (a) TEM image, and (b, c) EDS line profiling of Ni2P/BVO.

**
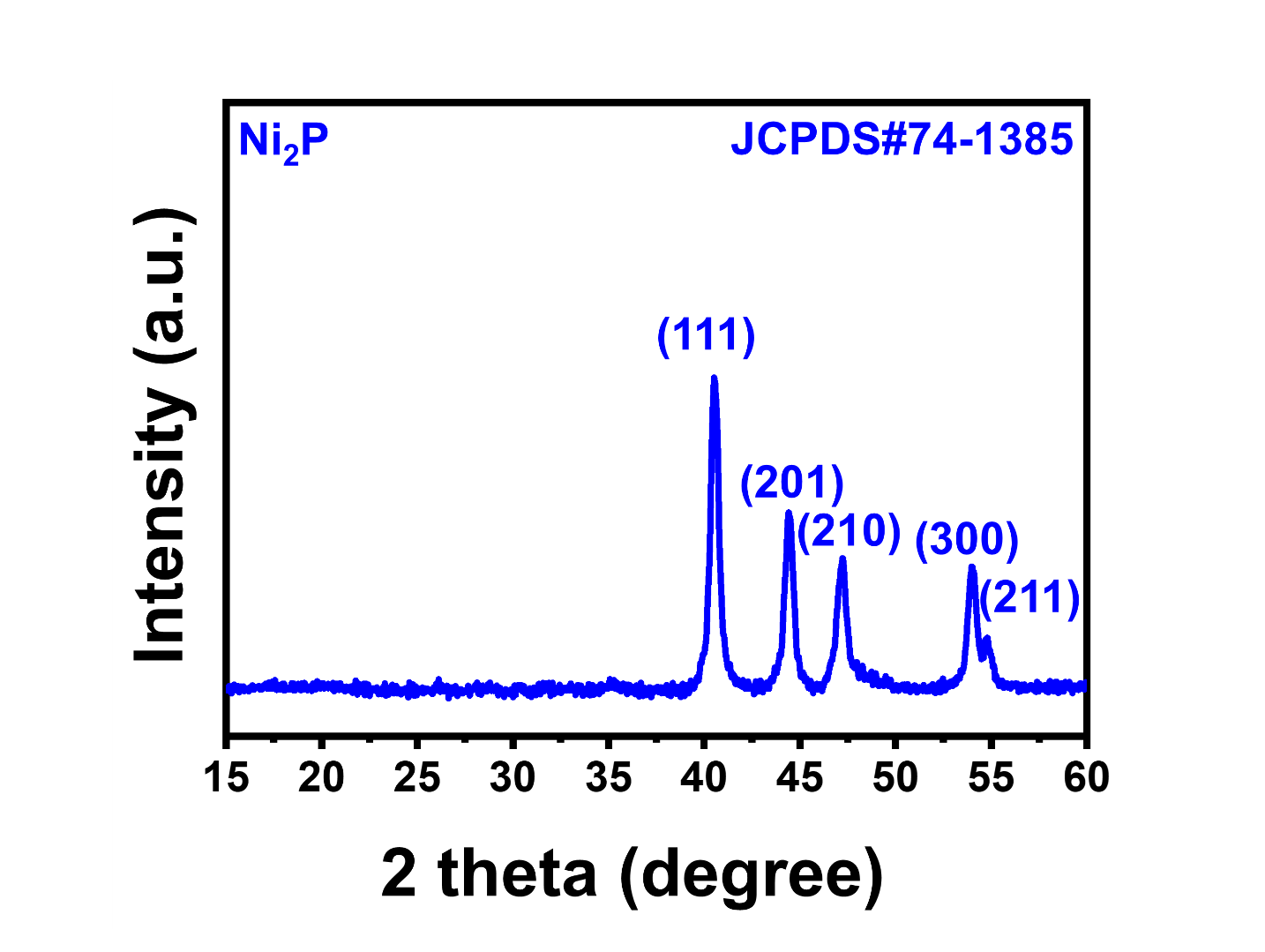
**

**Fig. S11.**  XRD pattern of Ni_2_P nanocrystals.


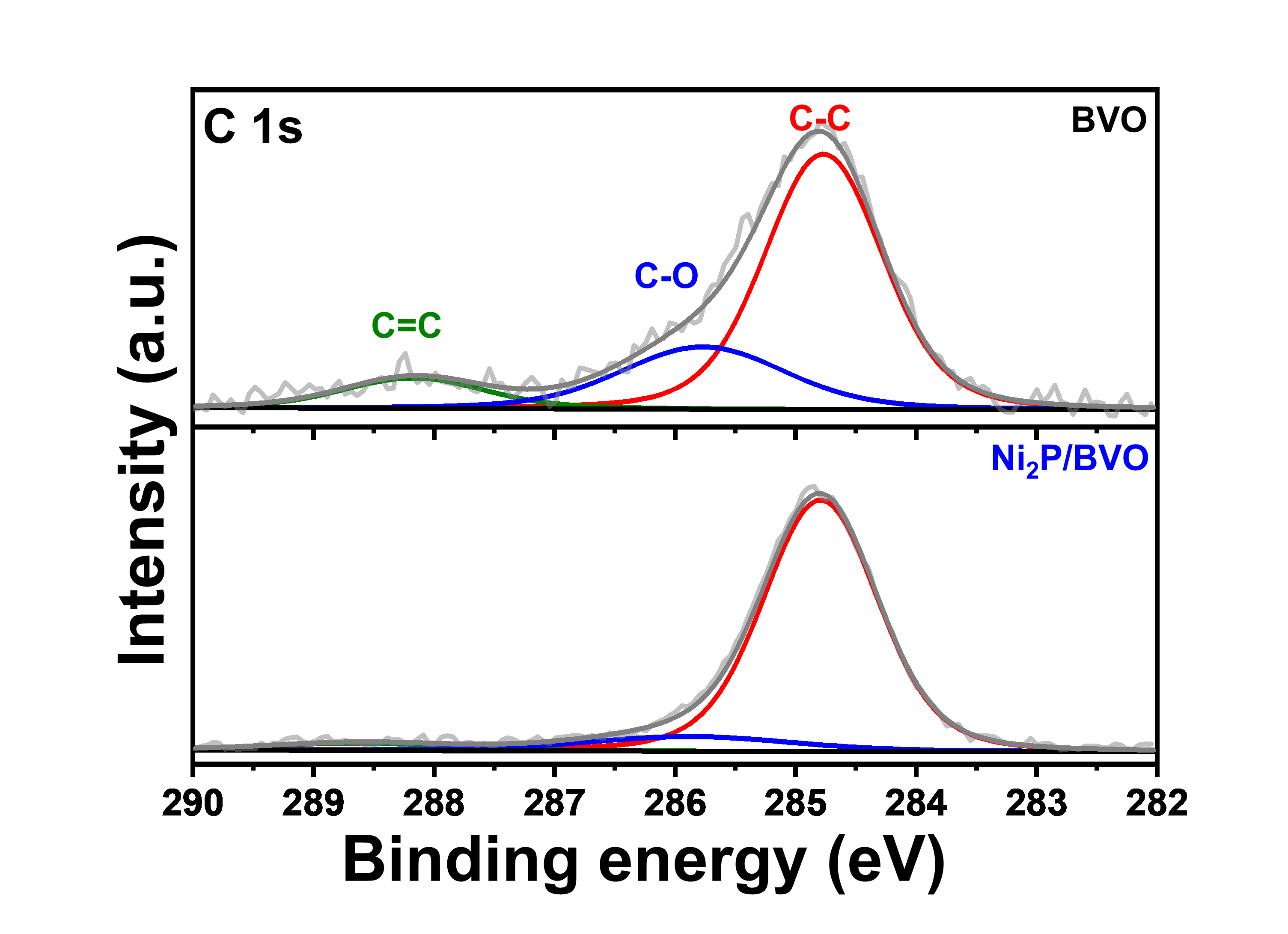


**Fig. S12.** XPS spectra of C1s of BVO, and Ni_2_P/BVO photoanodes.


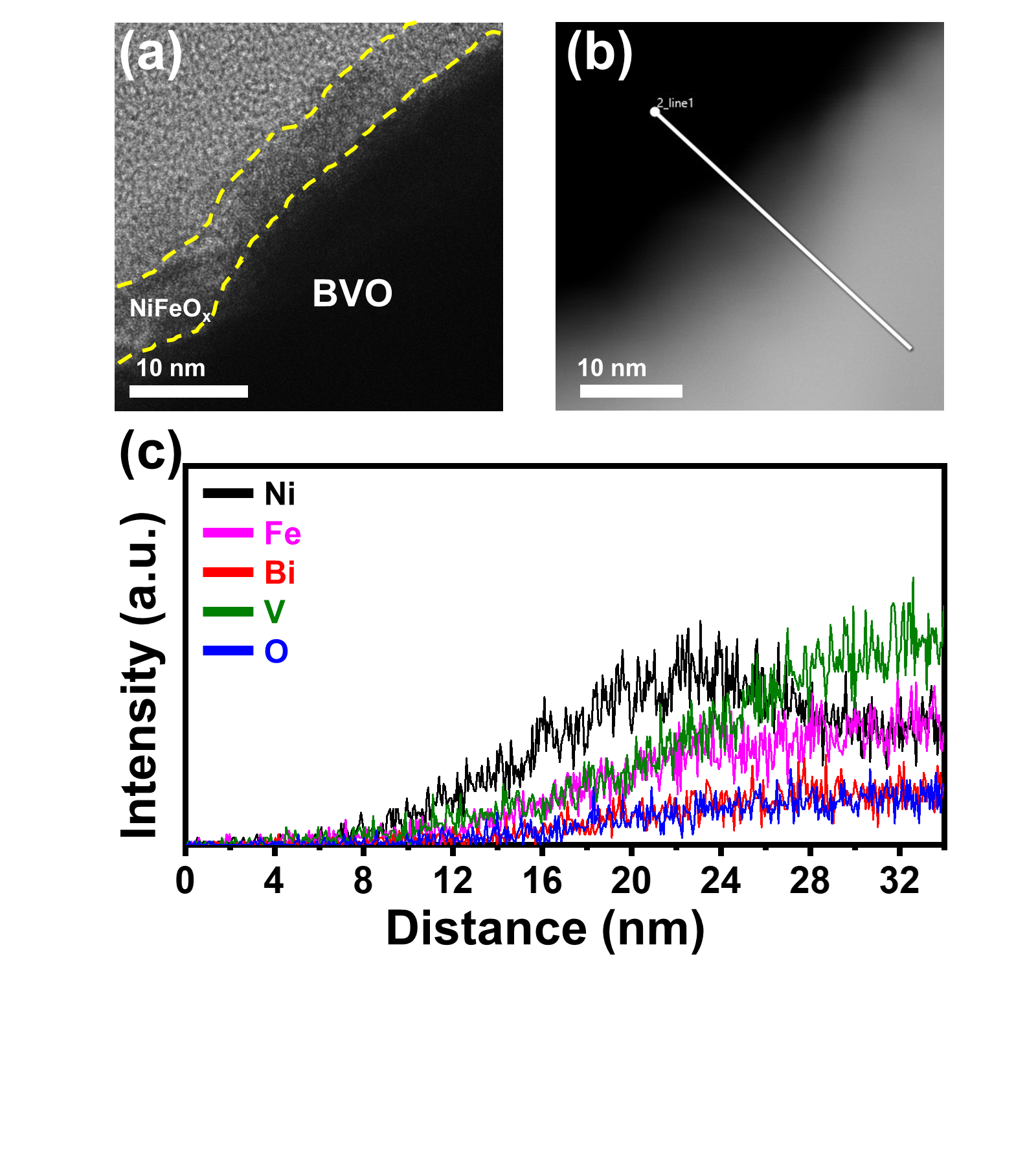


**Fig. S13.** (a) TEM image, and (b, c) EDS line profiling of NiFeO_x_/BVO.

**
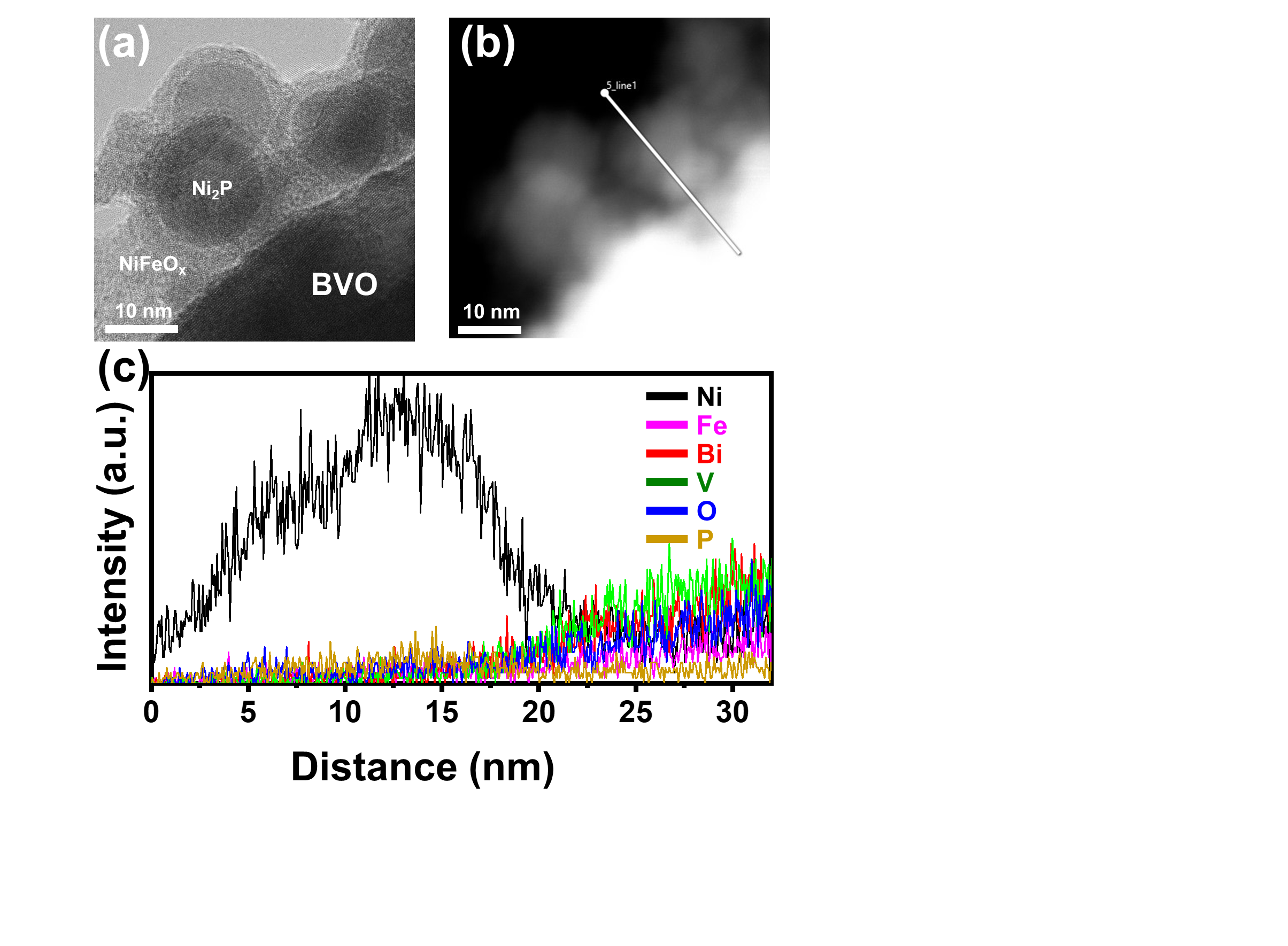
**

**Fig. S14.** (a) TEM image, and (b, c) EDS line profiling of NiFeO_x_/Ni_2_P/BVO.


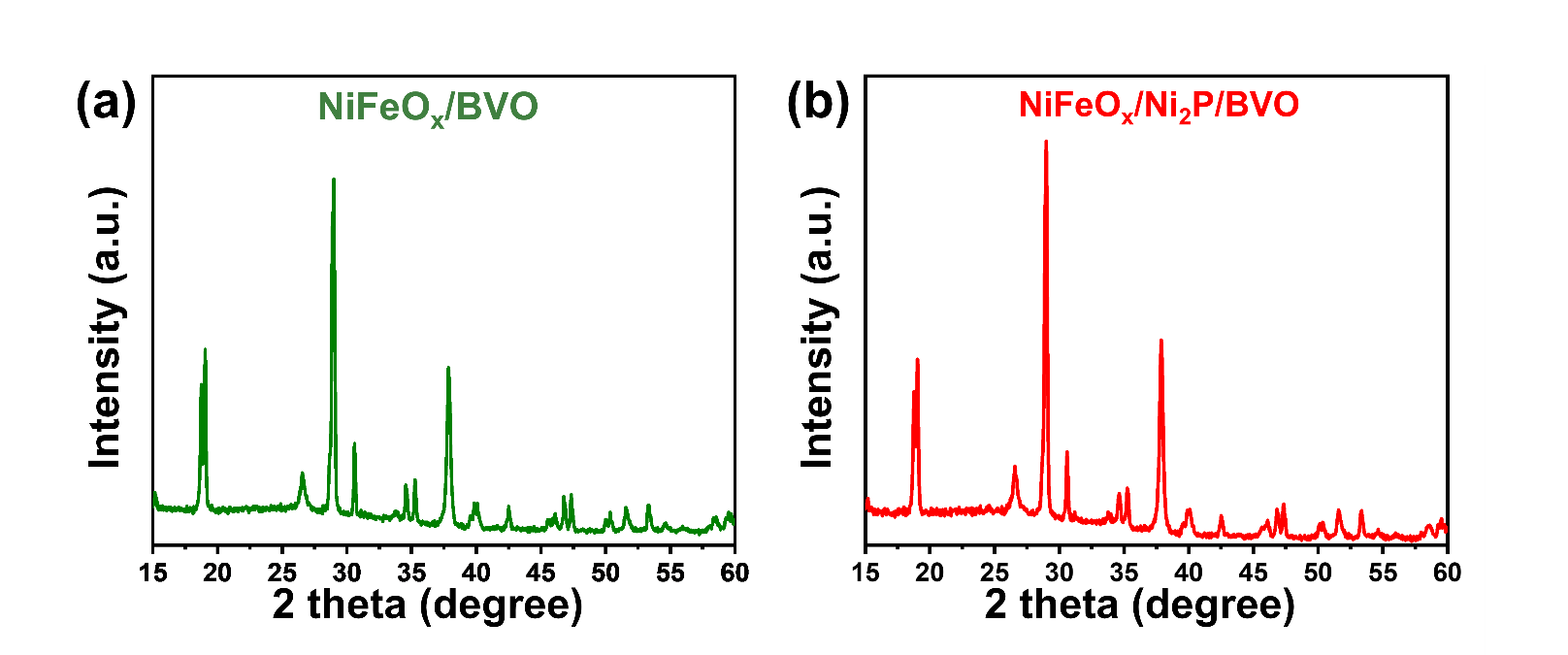


**Fig. S15.** XRD spectra of (a) NiFeO_x_/BVO, (b) NiFeO_x_/Ni_2_P/BVO.


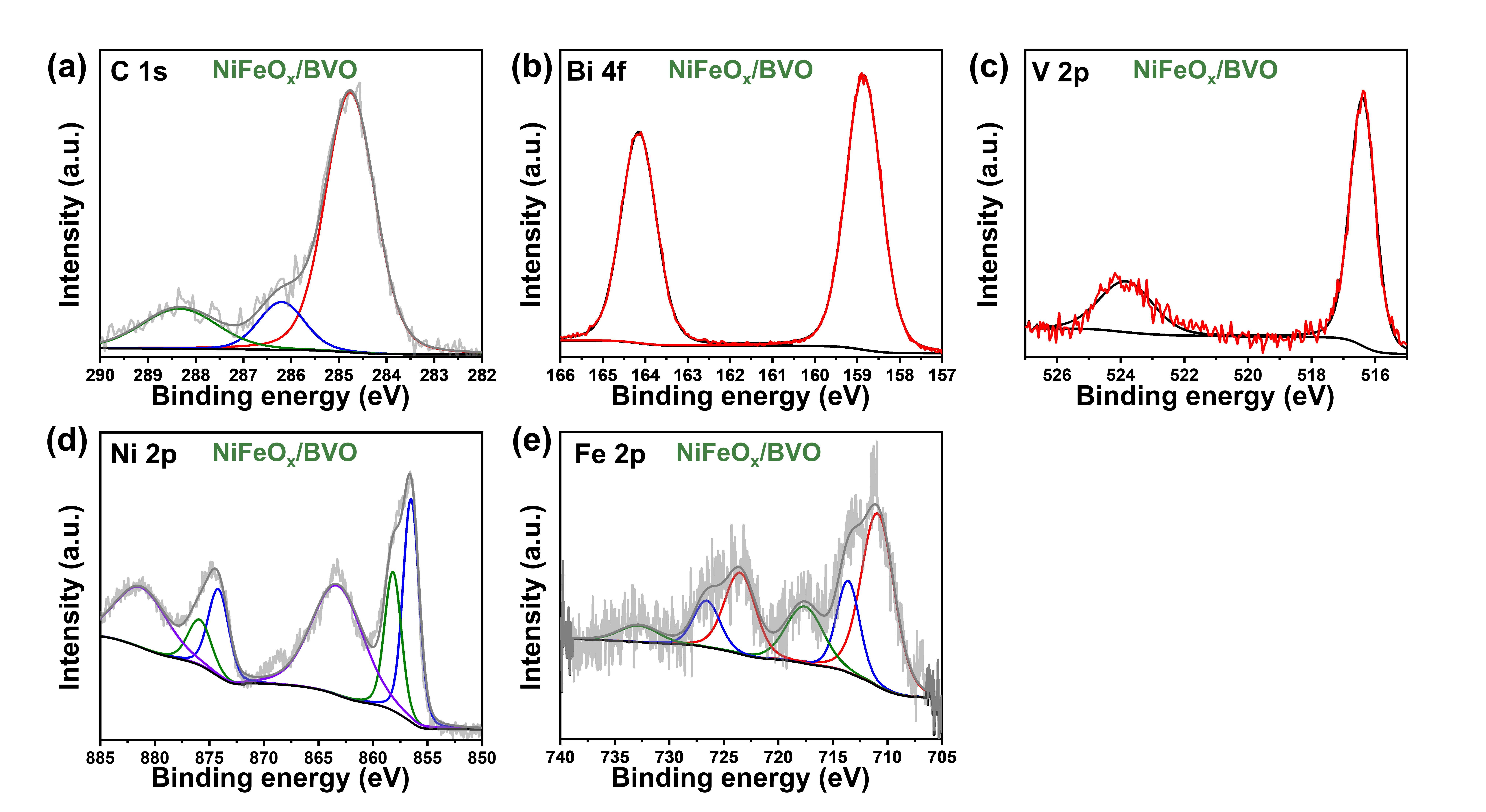


**Fig. S16.** High-resolution XPS (a) C 1s, (b) Bi 4f, (c) V 2p, (d) Ni 2p, and (e) Fe 2p spectra of NiFeO_x_/BVO.


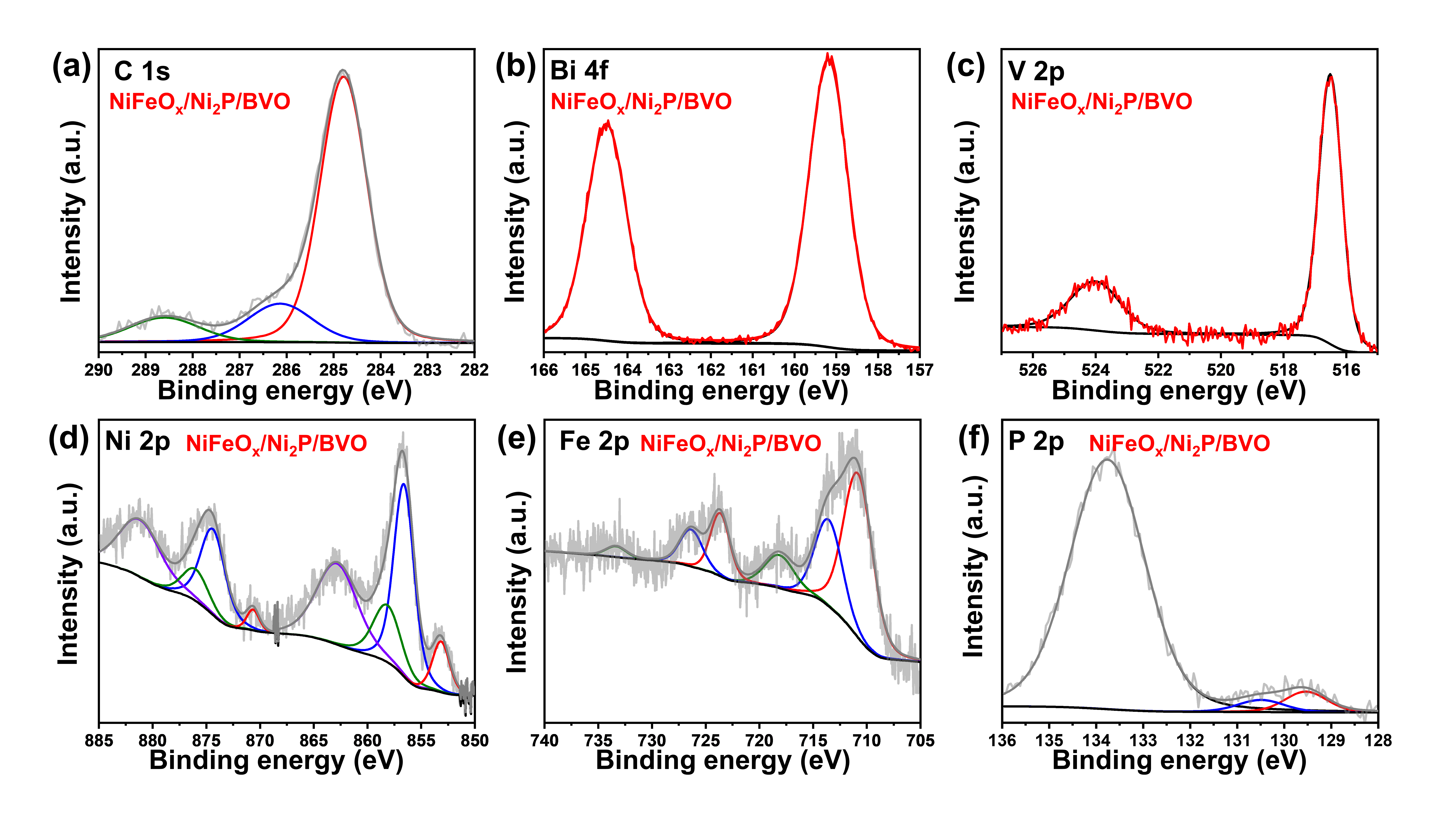


**Fig. S17.** High-resolution XPS (a) C 1s, (b) Bi 4f, (c) V 2p, (d) Ni 2p, (e) Fe 2p, and (f) P 2p spectra of NiFeO_x_/Ni_2_P/BVO.


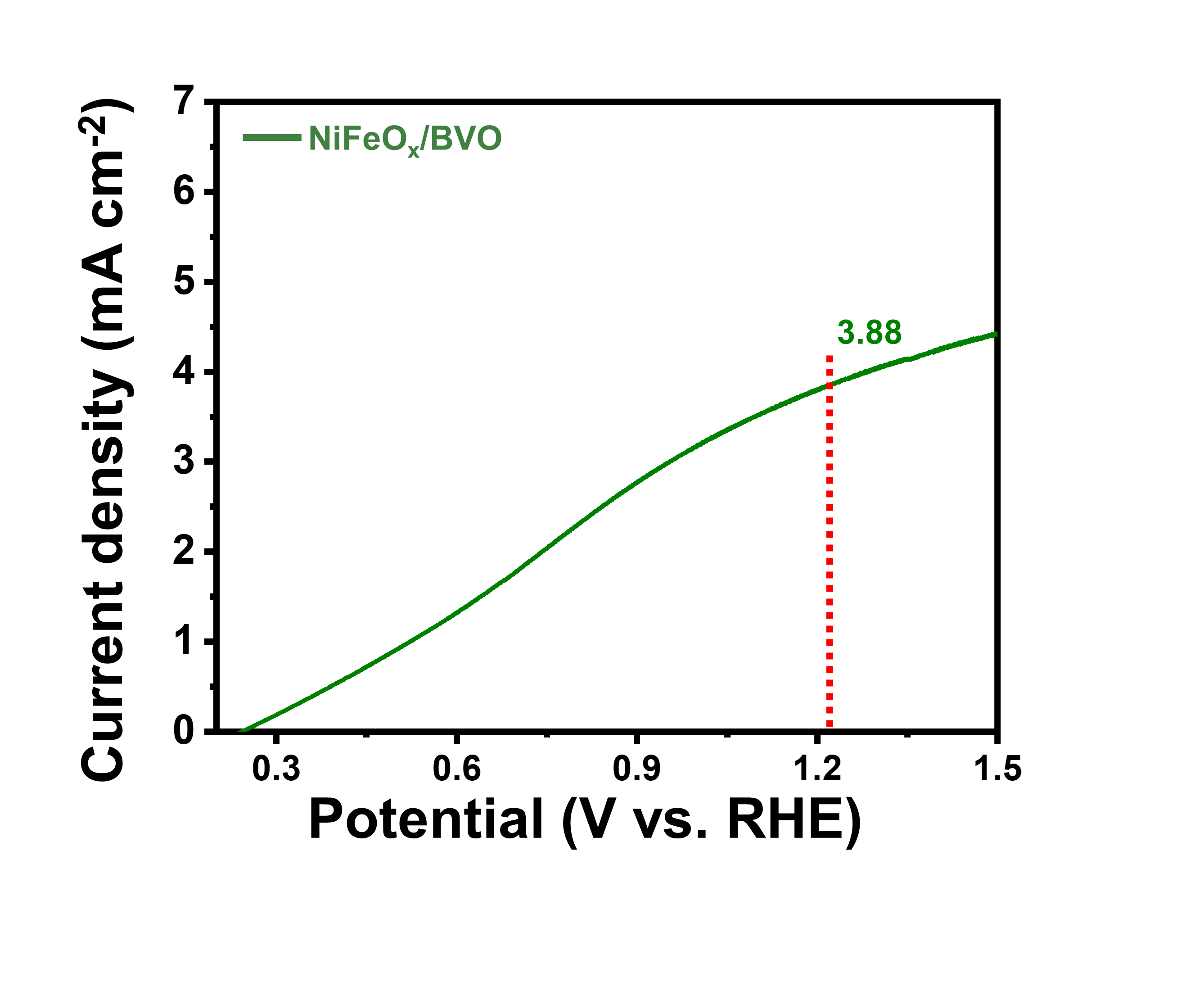


**Fig. S18.** J-V curve of NiFeO_x_/BVO.


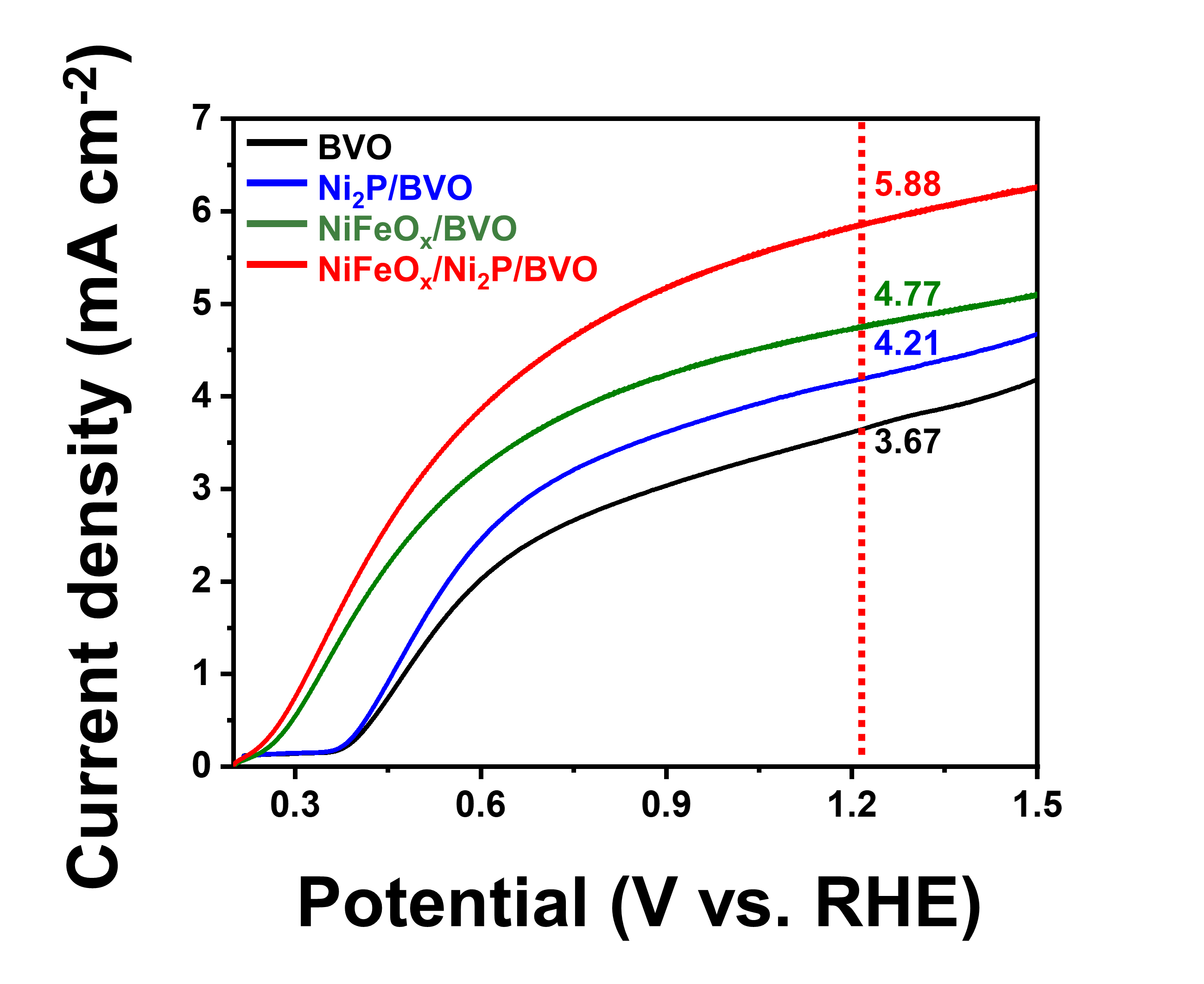


**Fig. S19.** J-V curves of the BVO-based photoanodes with hole scavenger.

**
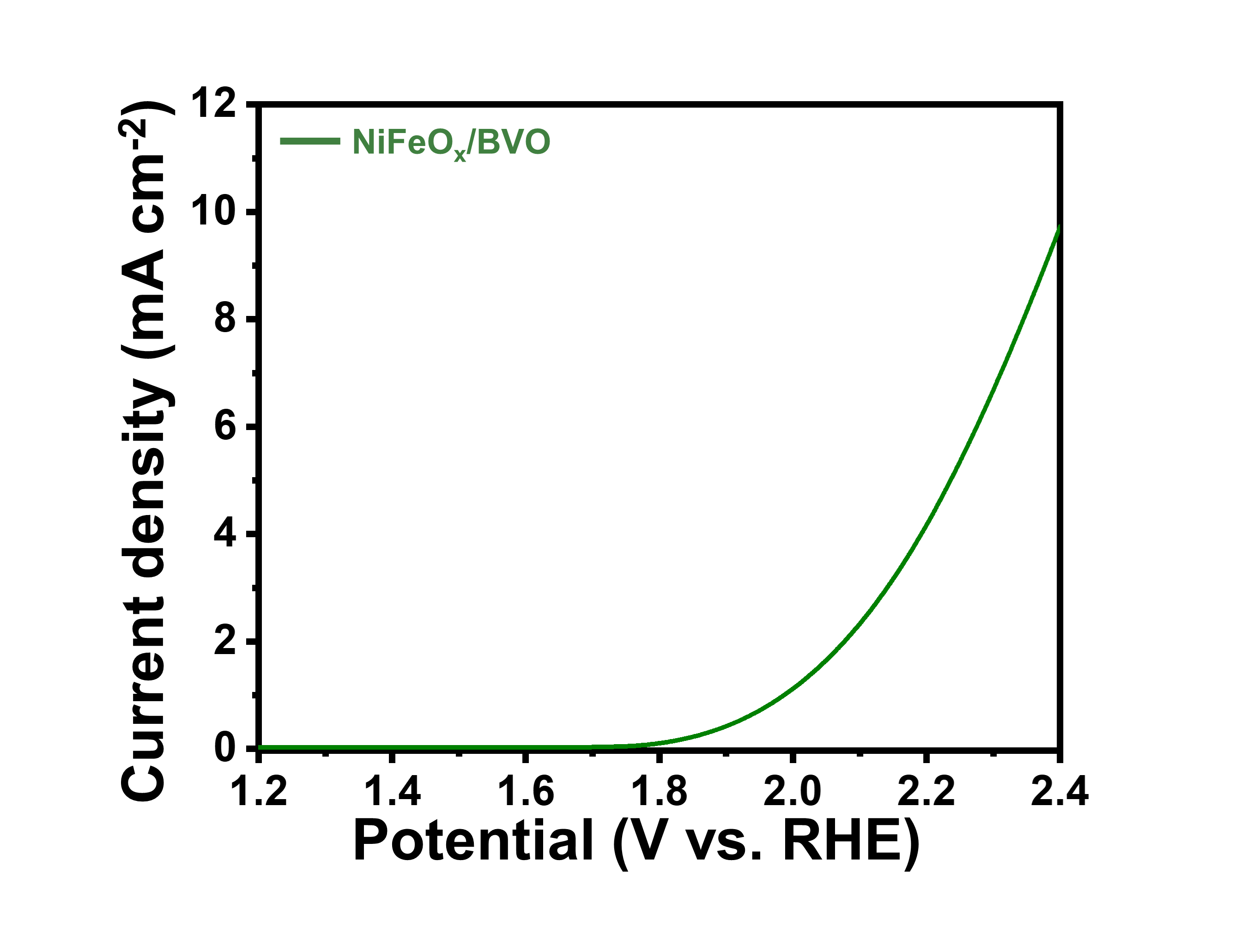
**

**Fig. S20.** J-V curve of NiFeO_x_/BVO without illumination.

**
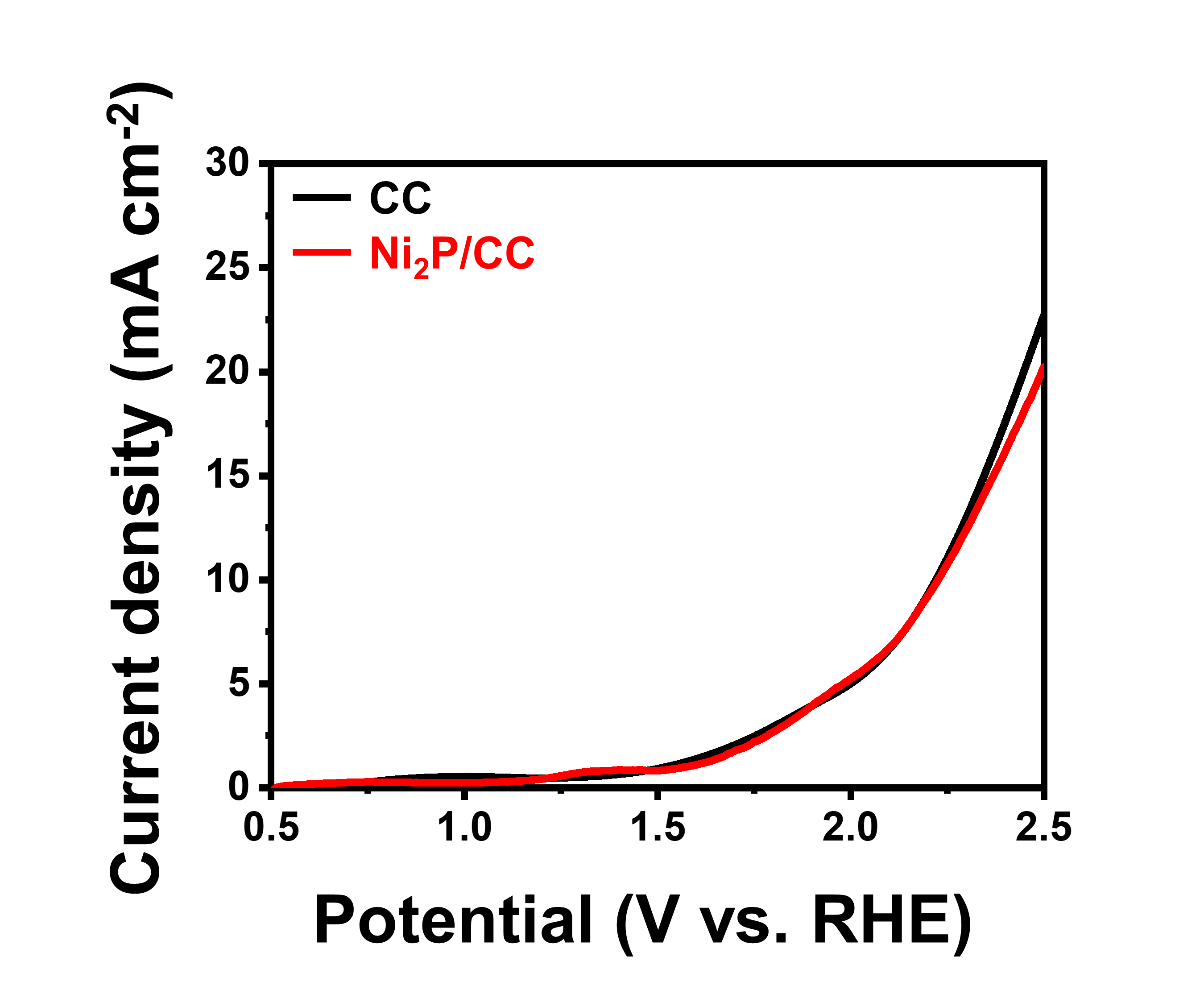
**

**Fig. S21.** J-V curves of CC and Ni2P/CC.


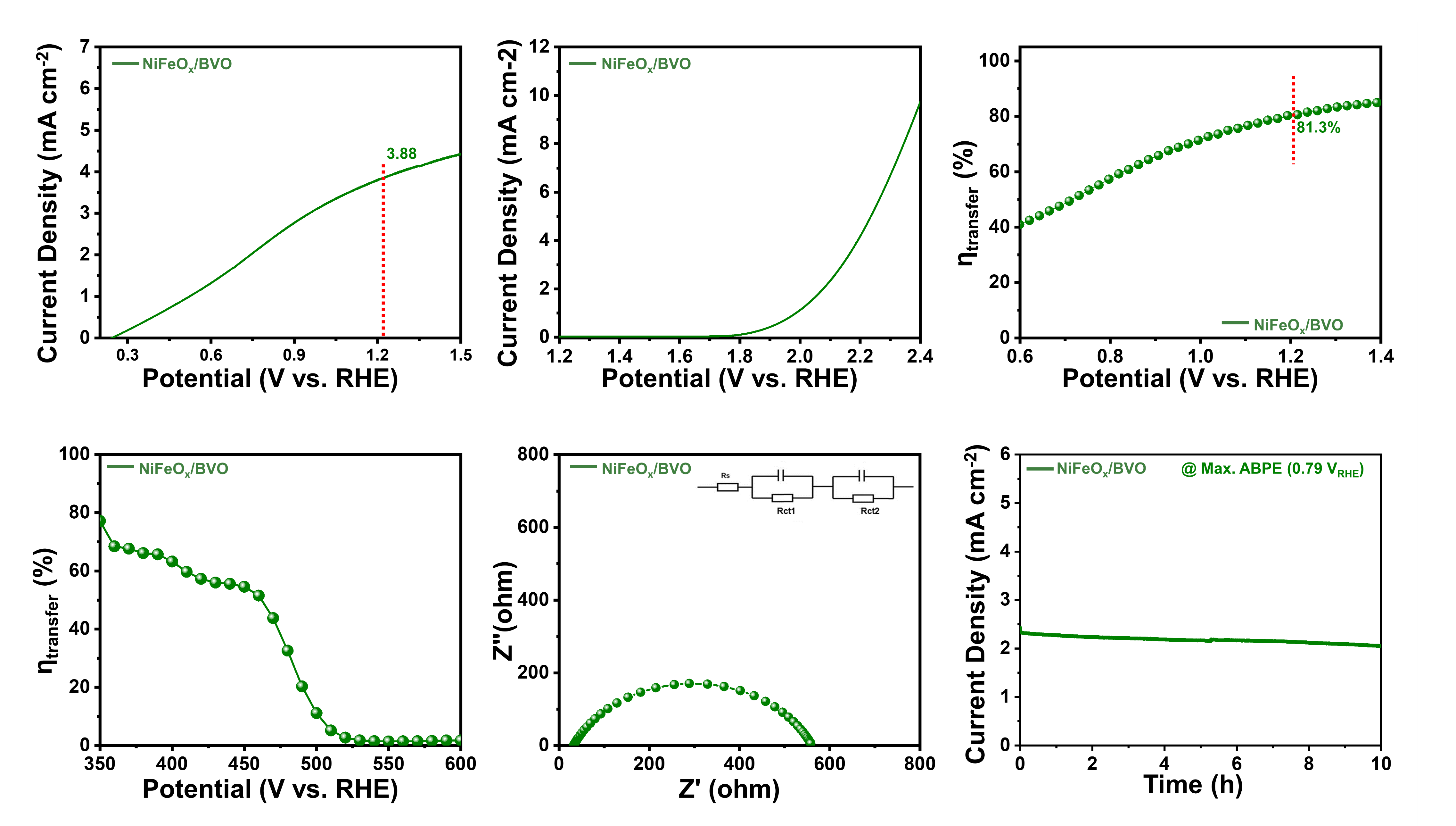


**Fig. S22.** Charge transfer efficiency of NiFeOx/BVO.


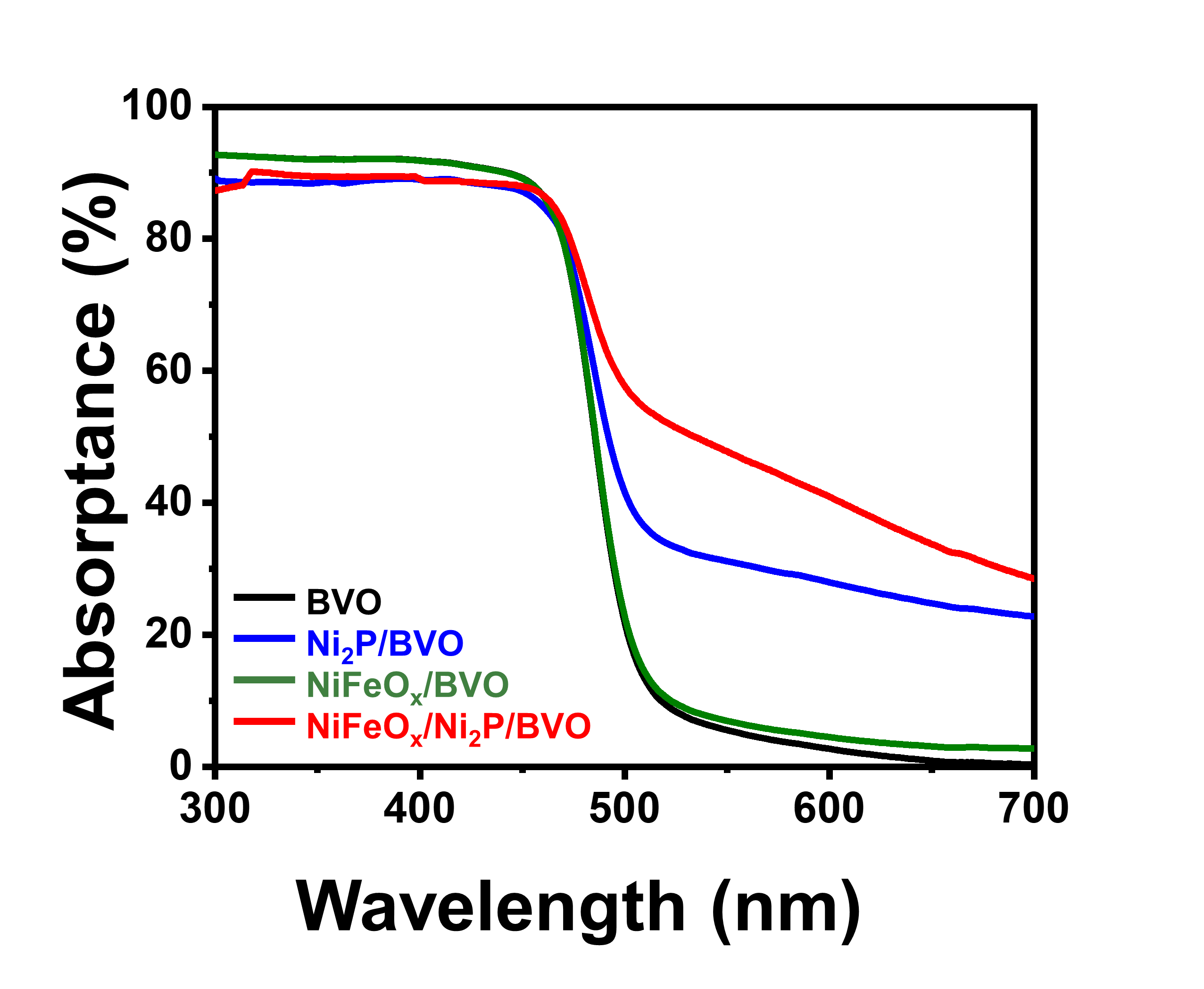


**Fig. S23.** UV-vis absorptance spectra of the BVO-based photoanodes.


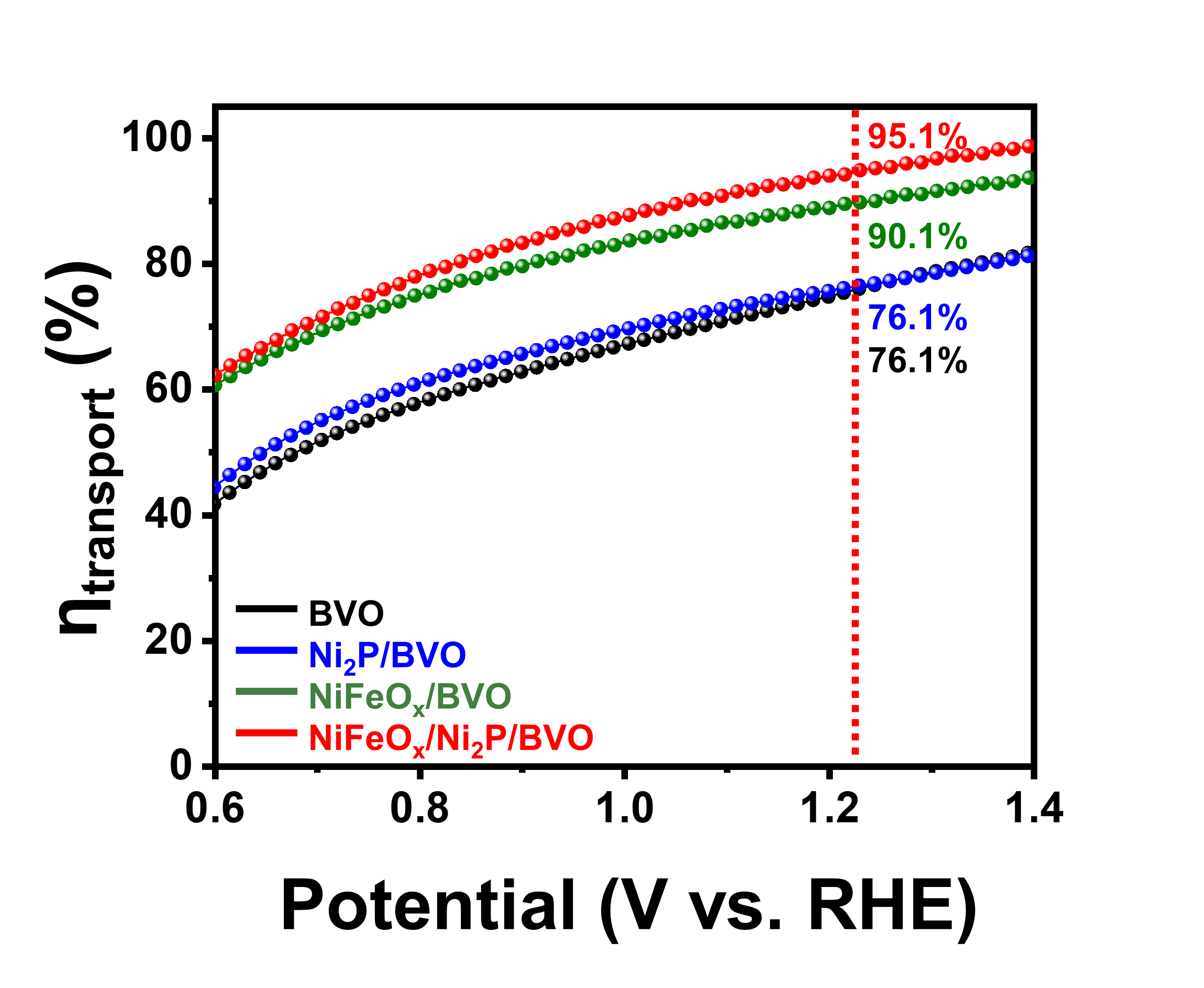


**Fig. S24.** Charge transport efficiency plots versus potential of BVO-based photoanodes.


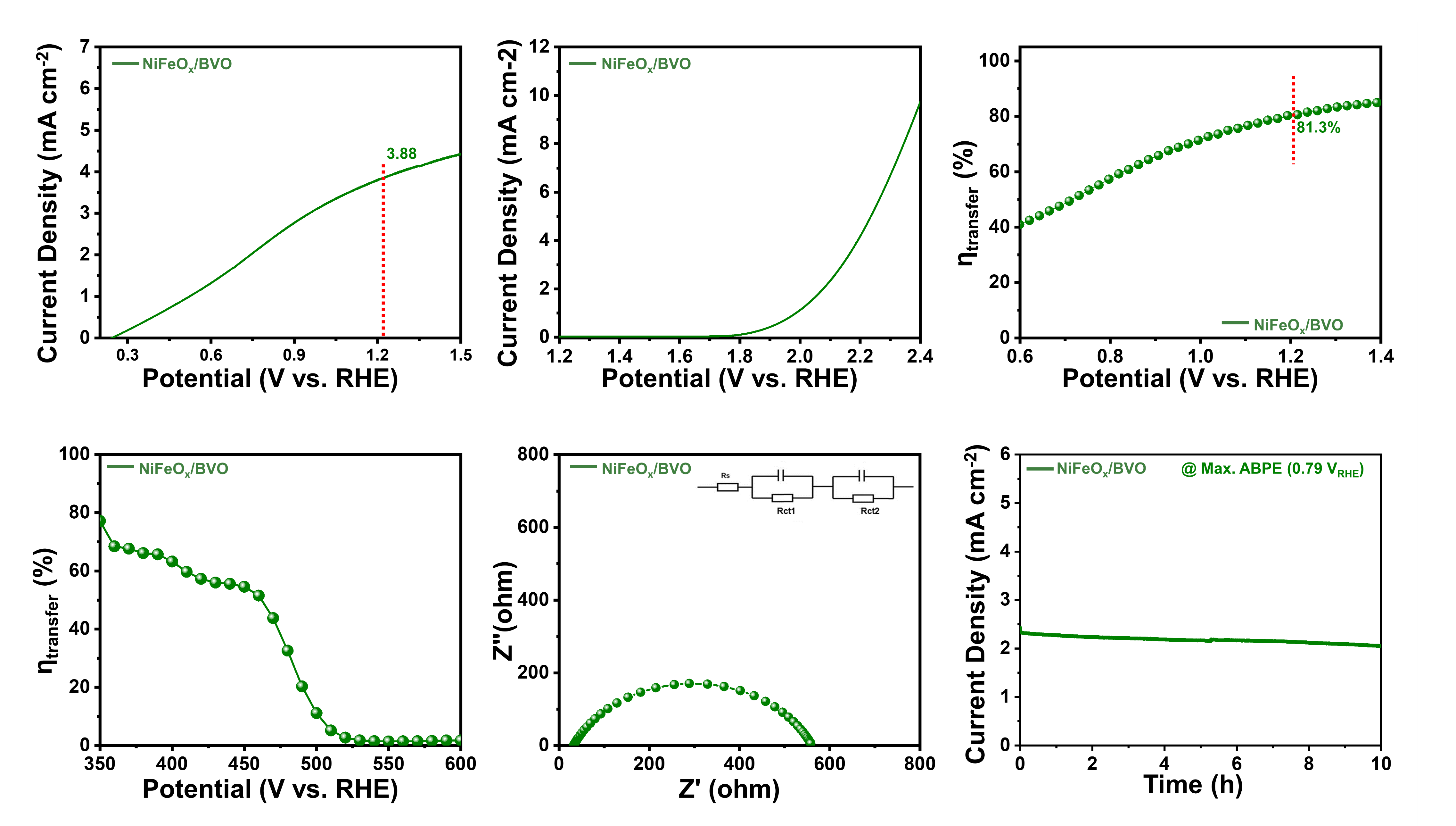


**Fig. S25.** IPCE sepctra of NiFeOx/BVO.


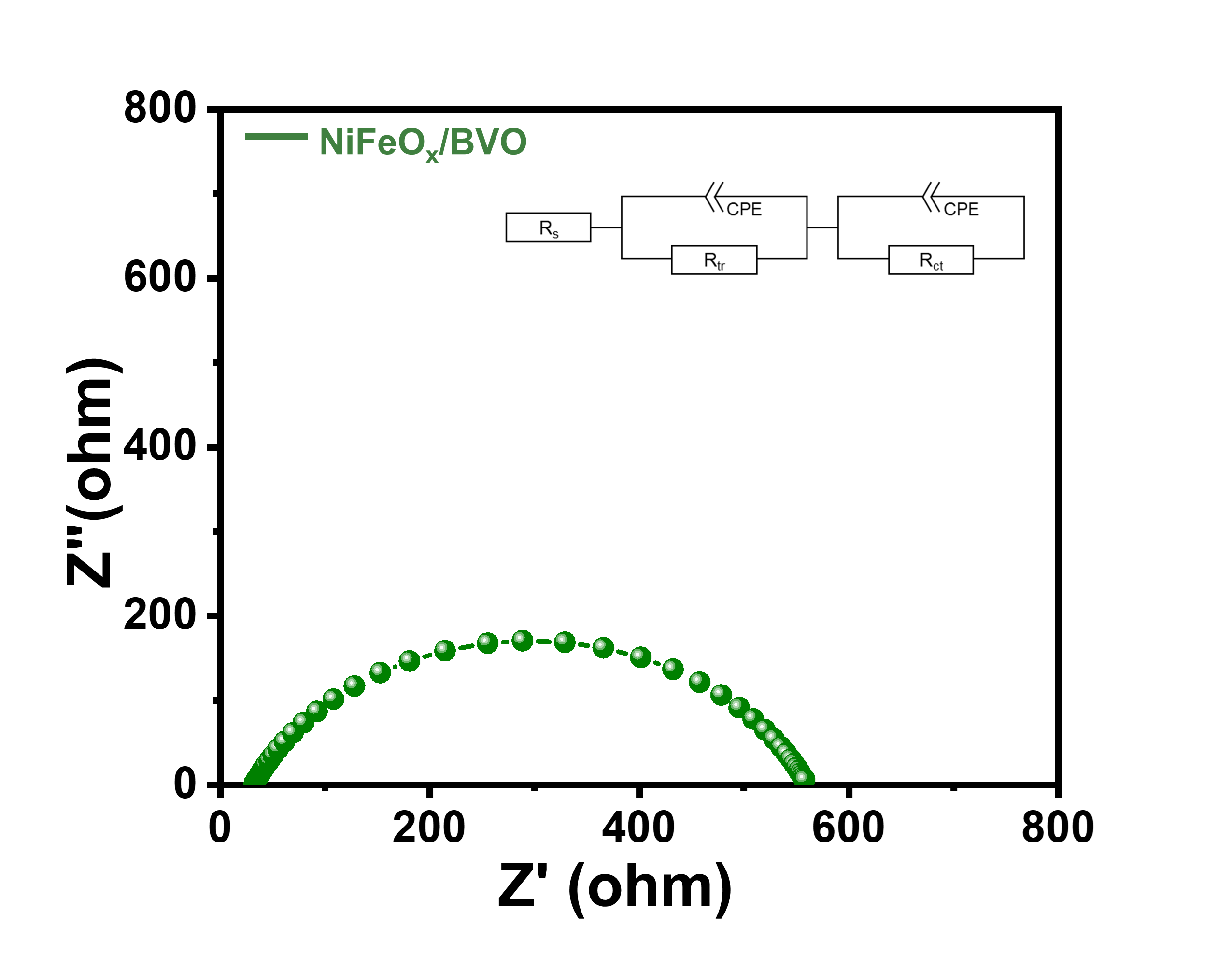


**Fig. S26.** Nyquist curve of NiFeOx/BVO with bias at 1.23 V_RHE_ under illumination.





**Fig. S27.** (a) IMPS Nyquist plot at 1.23 V_RHE_. (b) k_ct_ of the BVO based photoanodes.


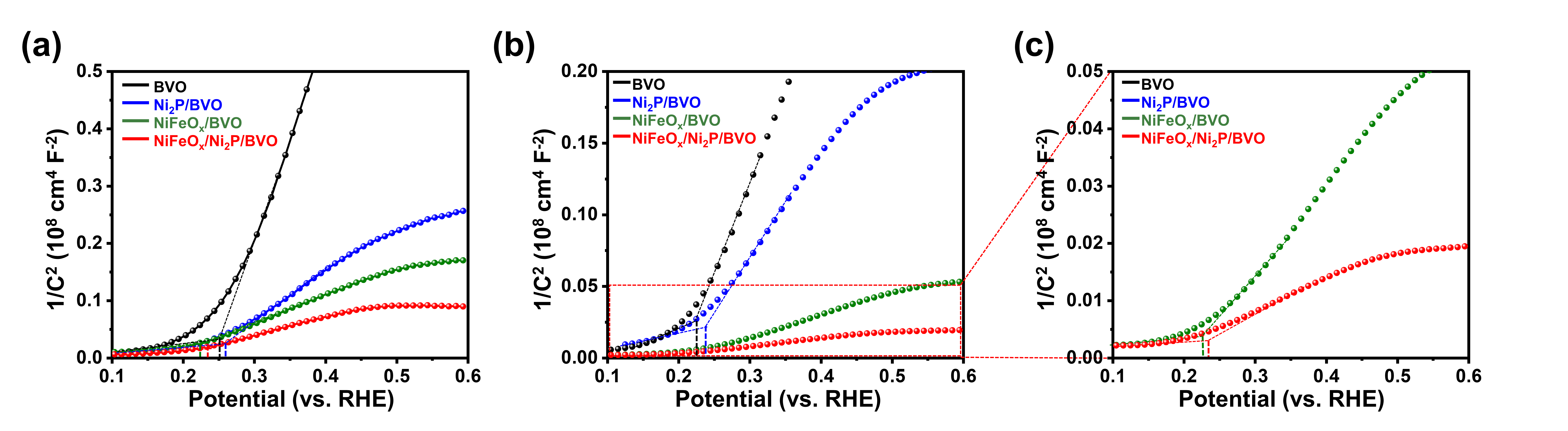


**Fig. S28.** Mott-Schottky plots of the BVO based samples (a) without and (b-c) with illumination.


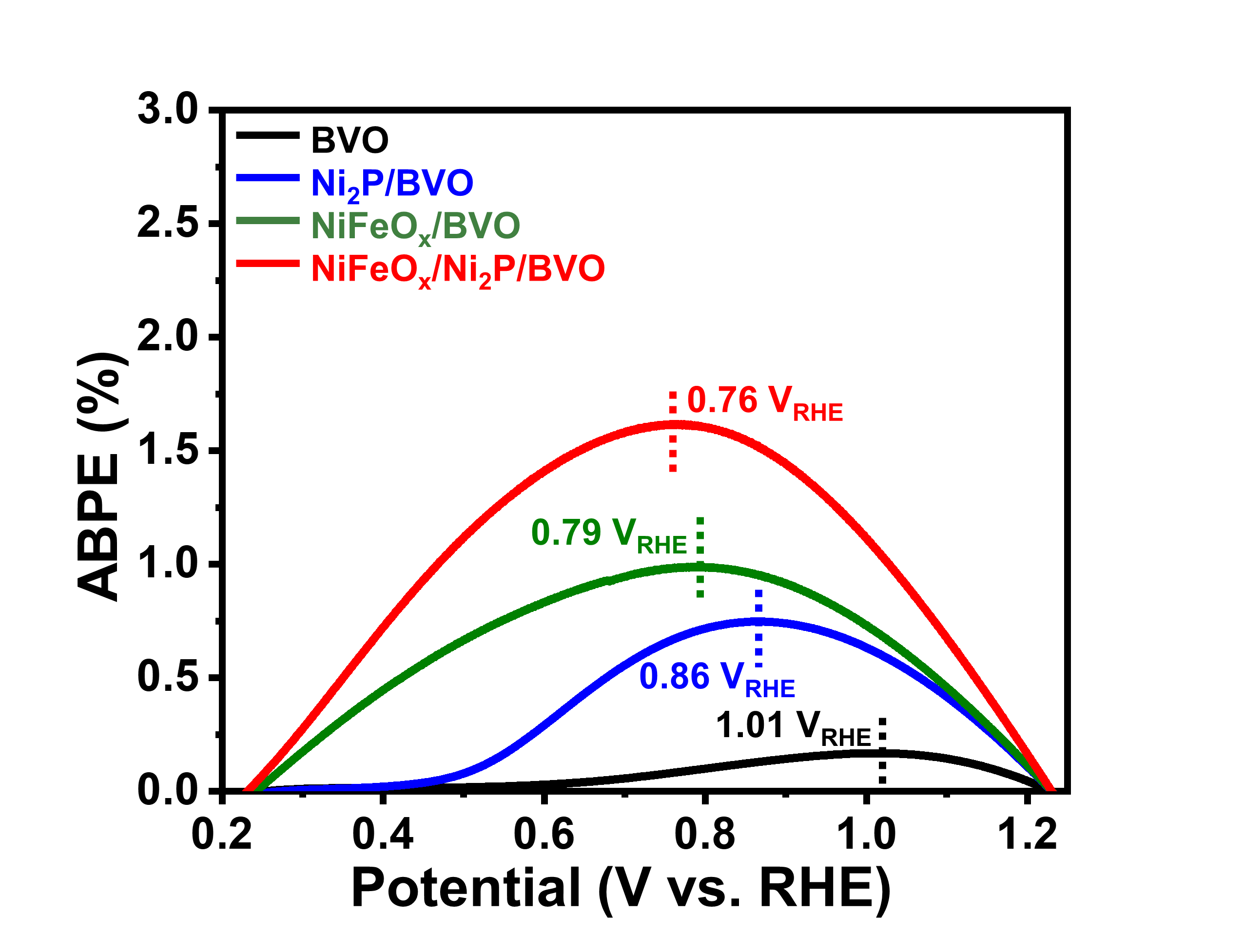


**Fig. S29.** ABPE of the BVO based photoanodes.

**
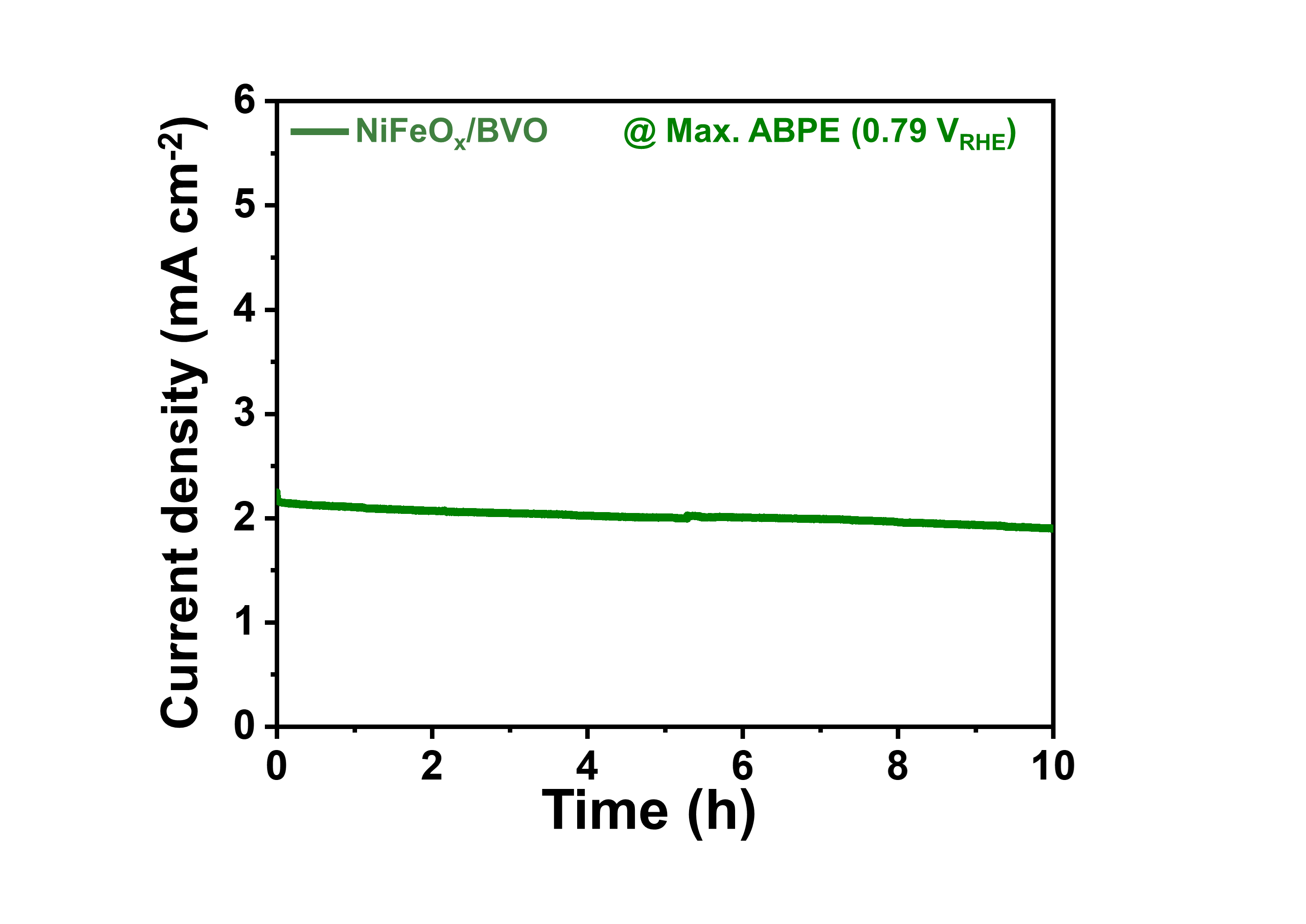
**

**Fig. S30.** Long-term stability test of NiFeO_x_/BVO photoanode at 0.79 V_RHE_.


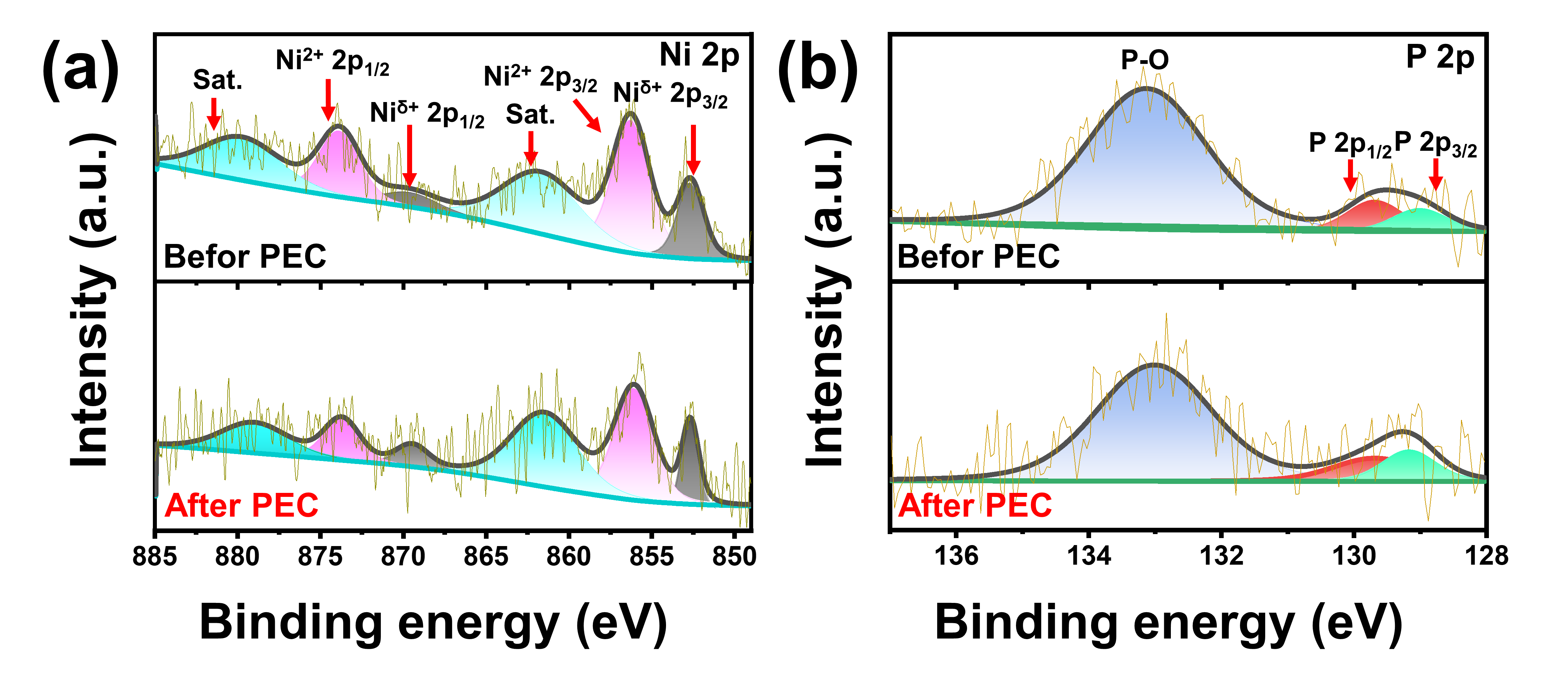


**Fig. S31.** High-resolution XPS (a) Ni 2p, and (b) P 2p spectra of Ni_2_P/BVO and BVO.

**
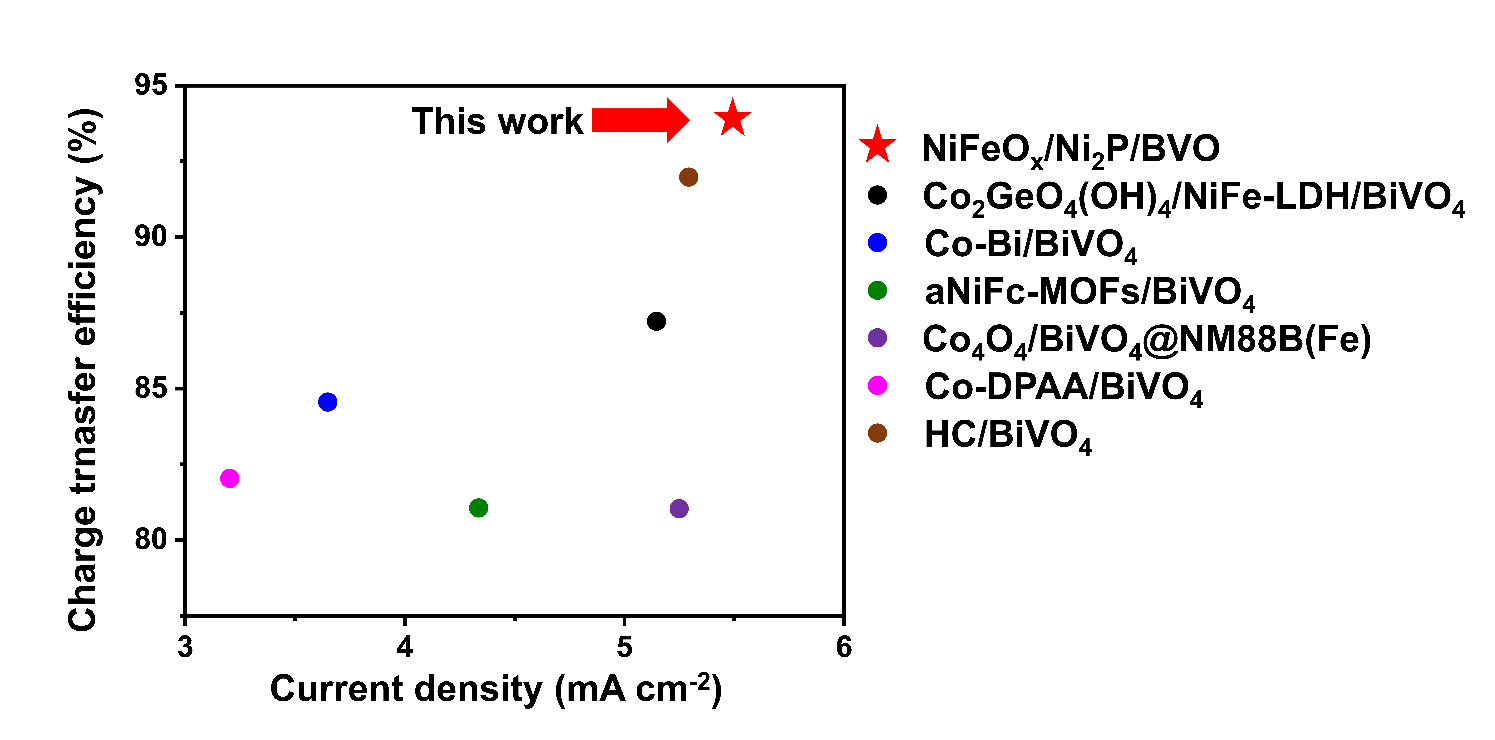
**

**Fig. S32.** Photoelectrochemical water oxidation performance comparison for recently reported BVO-based electrocatalysts based on Table S1.


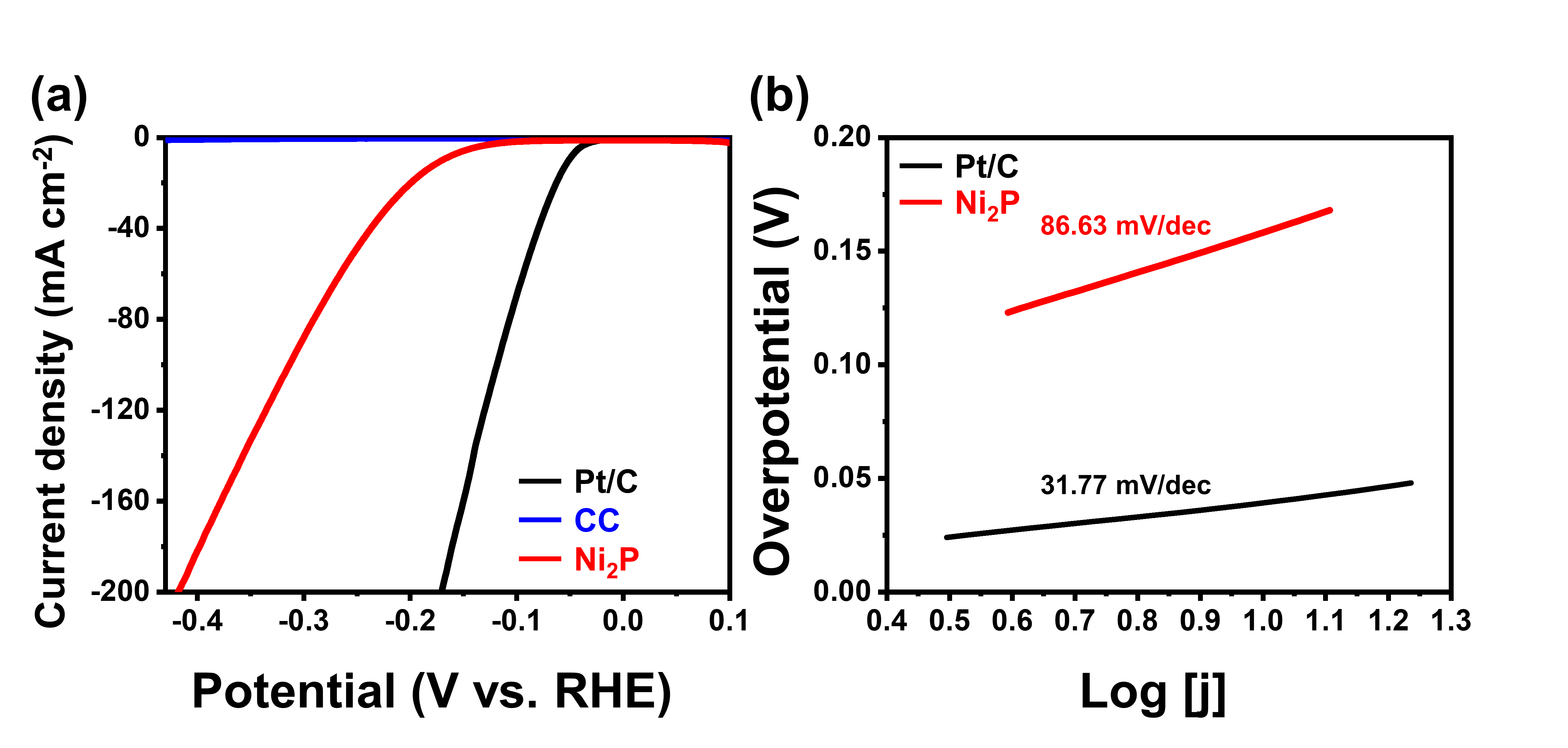


**Fig. S33.** (a) Hydrogen evolution reaction (HER) performance. (b) Tafel plots for HER of Ni_2_P.

**
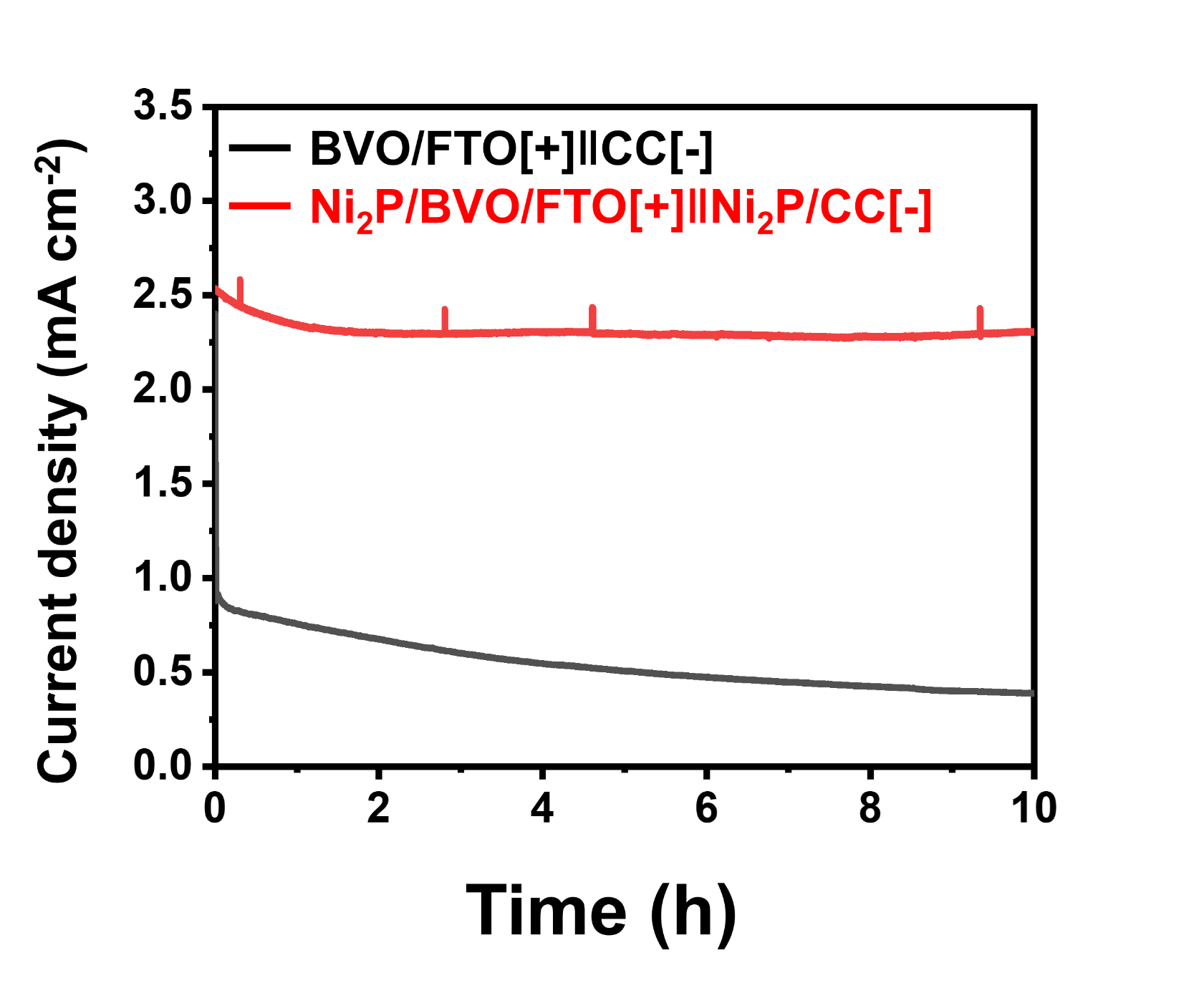
**

**Fig. S34.** Current density vs. time curves for the long-term operation of solar-assisted water splitting with the 2-electrode configuration of PEC cells under 1 sun illumination at 1.23 V in KPi pH 7.


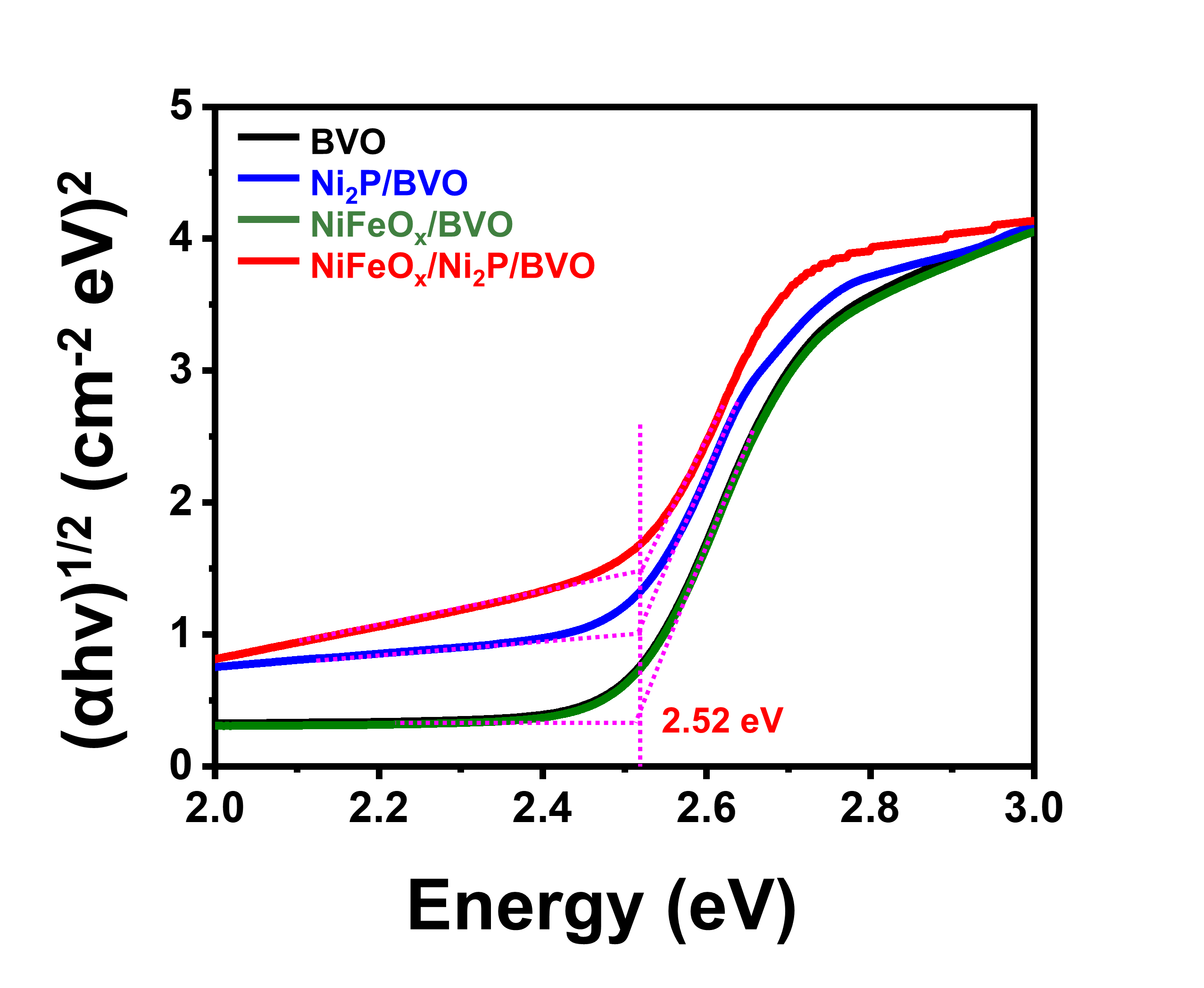


**Fig. S35.** Taus plots for the BVO based photoanodes.


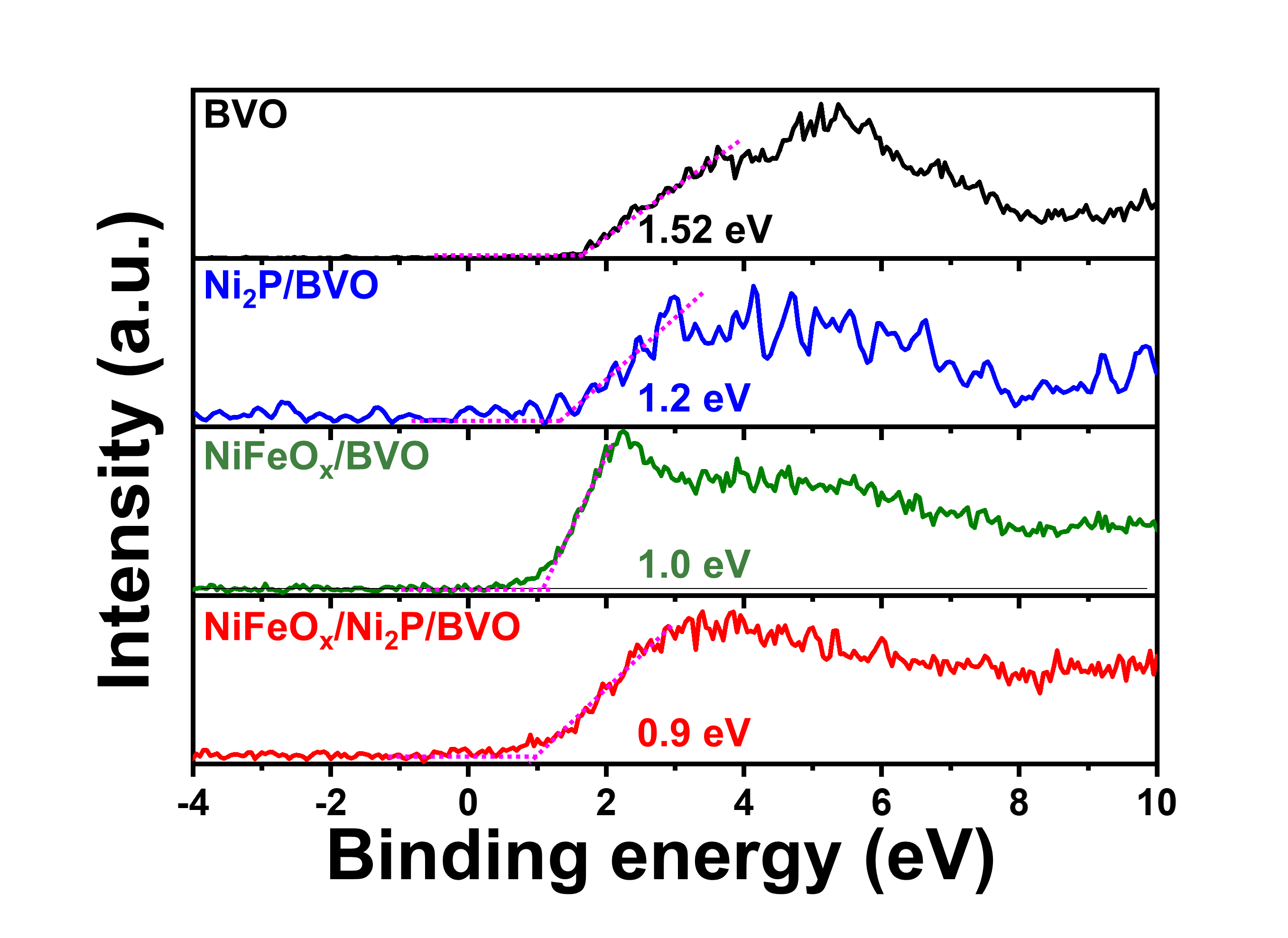


**Fig. S36.** XPS valence band spectra of the BVO based photoanodes.


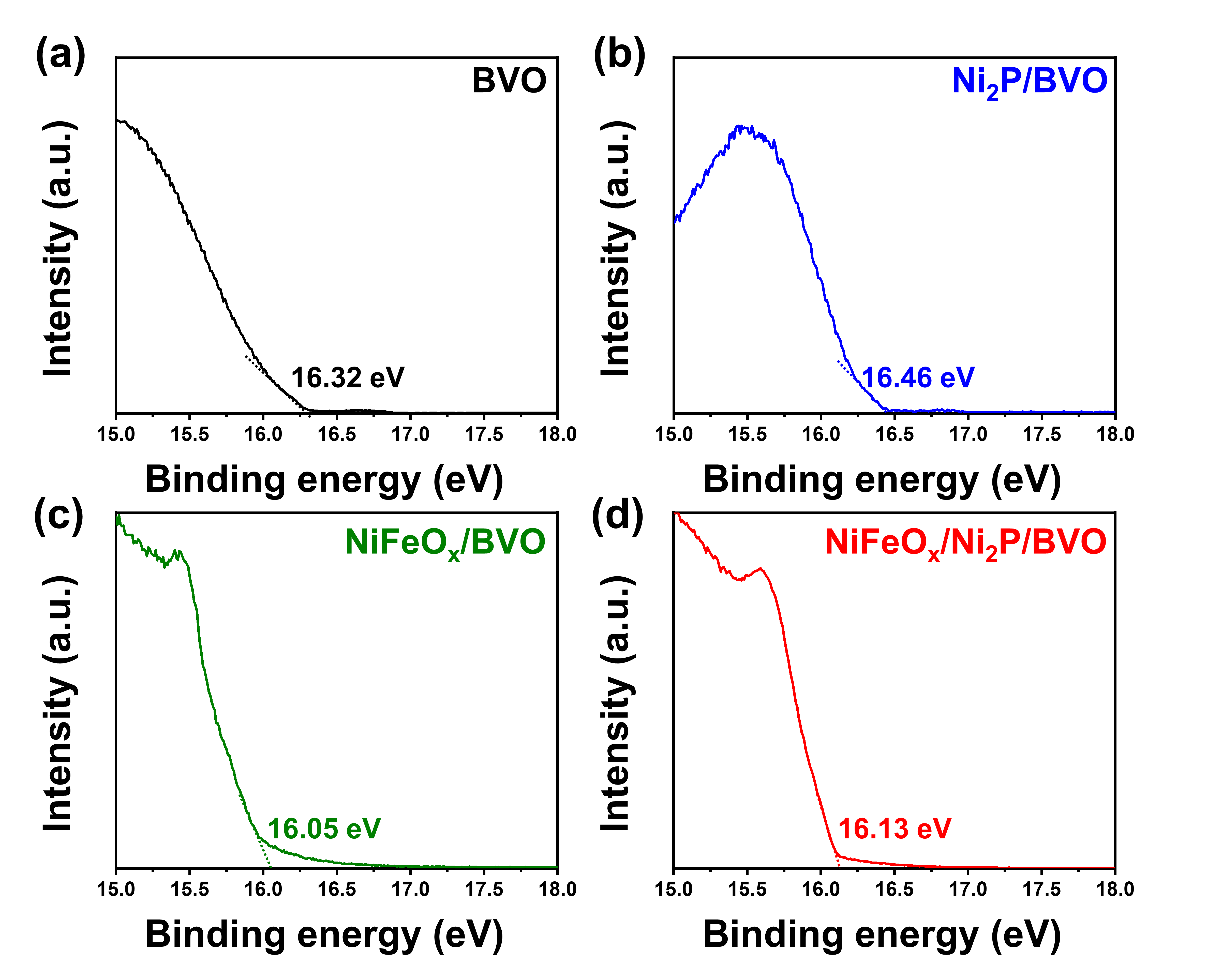


**Fig. S37.** UPS spectra of the BVO based photoanodes.


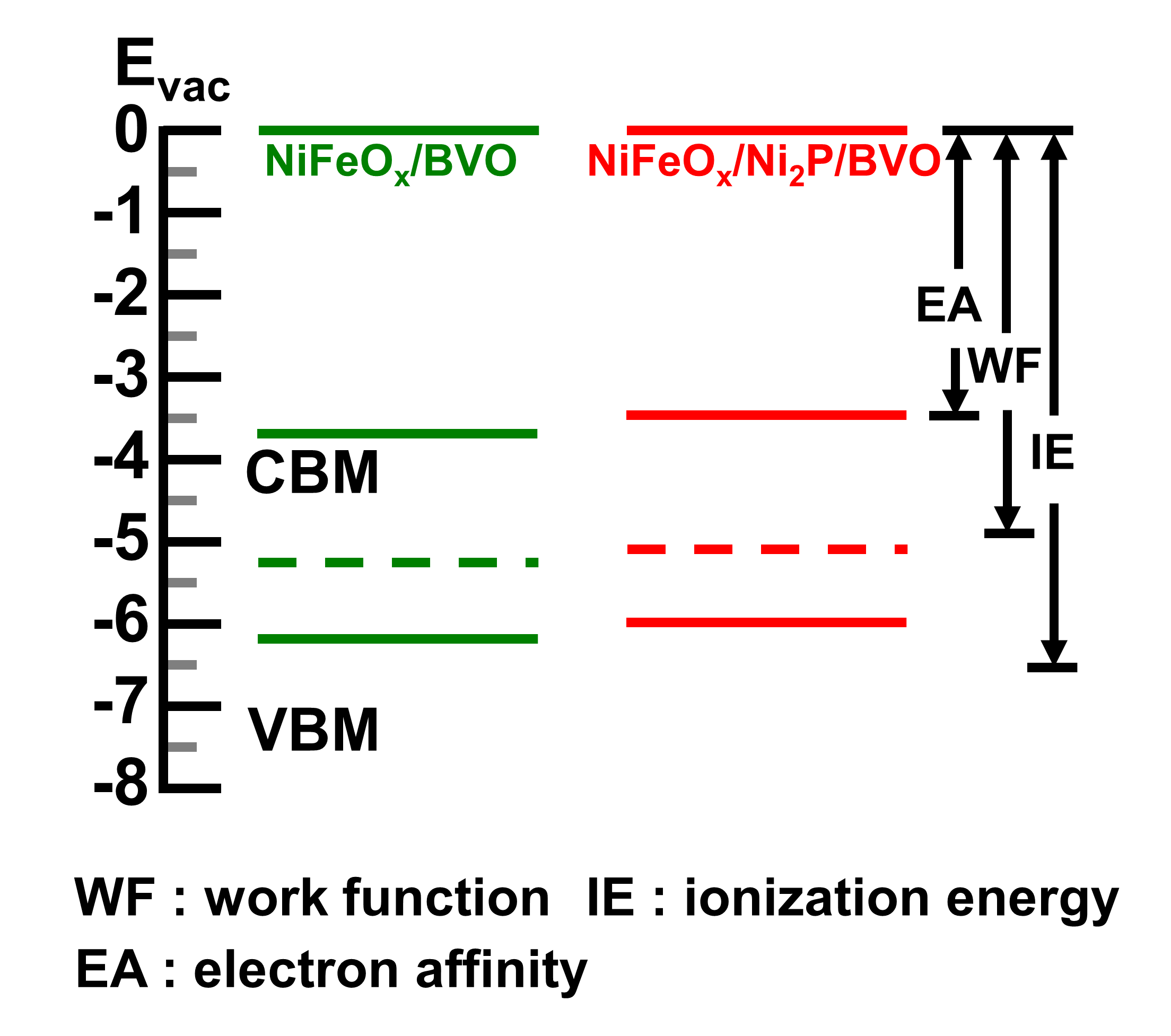


**Fig. S38.** Band structure diagram of NiFeO_x_/BVO and NiFeO_x_/Ni_2_P/BVO.

**Table S1:** EIS Nyquist plot results of BVO and Ni_2_P/BVO photoanodes

|  | **R_s_** | **R_ct_** | **R_tr_** |
| --- | --- | --- | --- |
| BVO | 33.12 | 362.7 | 474.1 |
| Ni_2_P/BVO | 30.89 | 108.1 | 469.3 |
| NiFeO_x_/BVO | 31.90 | 112.8 | 397.1 |
| NiFeO_x_/Ni2P/BVO | 34.05 | 90.05 | 220.6 |

**Table S2:** Flat band potential and donor densities of BVO based photoelectrodes

|  | **V_fb_ (V_RHE_)** | **N_d_ (x 10^17^ m^-3^)** |
| --- | --- | --- |
| BVO | 0.248 | 5.64 |
| Ni_2_P/BVO | 0.263 | 2.47 |
| NiFeO_x_/BVO | 0.218 | 42.3 |
| NiFeO_x_/Ni_2_P/BVO | 0.233 | 66.9 |

**Table S3:** Flat band potential of BVO based photoelectrodes under illumination

| Catalyst | Flat band potential (V_RHE_) |
| --- | --- |
| BVO | 0.225 |
| Ni_2_P/BVO | 0.240 |
| NiFeO_x_/BVO | 0.234 |
| NiFeO_x_/Ni_2_P/BVO | 0.245 |

**Table S4:** Comparison of current density and charge transfer efficiency for recently reported BVO-based electrocatalysts

| Catalyst | Current density  (mA cm^-2^ @ 1.23 V_RHE_) | Charge transfer efficiency (%) | Reference |
| --- | --- | --- | --- |
| This work | 5.49 | 93.9 |  |
| Co_2_GeO_4_(OH)_4_/NiFe-LDH/BV | 5.15 | 87.2 | 3 |
| Co-Bi/BiVO_4_ | 3.65 | 84.6 | 4 |
| aNiFc-MOFs/BiVO_4_ | 4.34 | 81 | 5 |
| Co_4_O_4_/BiVO_4_@NM88B(Fe) | 5.26 | 81 | 6 |
| Co-DPAA/BiVO_4_ | 3.2 | 82 | 7 |
| HC/BiVO_4_ | 5.3 | 92 | 8 |

Supporting References

[1] Mustafa, R. R.; Sukor, R.; Mohd Nor, S. M.; Saari, N.; Mohsin, A. Z. Development of Methyl Ester Antibody-Based Competitive Indirect ELISA for Quantitative Detection of Mitragynine in Human Urine. *ACS Omega* 2023, 8 (50), 47412-47426. DOI: 10.1021/acsomega.3c02734.

[2] Kleis, J.; Jones, G.; Abild-Pedersen, F.; Tripkovic, V.; Bligaard, T.; Rossmeisl, J. Trends for Methane Oxidation at Solid Oxide Fuel Cell Conditions. *J. Electrochem. Soc.* 2009, 156 (12), B1447. DOI: 10.1149/1.3230622.

[3] Chi J; Wei Z; Guo W; et al. Enhanced Photoelectrochemical Water Splitting on BiVO_4_ Photoanode via Efficient Hole Transport Layers of NiFe-LDH. *ACS Catalysis*. 2025, 15 (13), 11293-11306. DOI: 10.1021/acscatal.5c02714

[4] Dell’Oro R; Sansotera M; Bianchi CL; Magagnin L. Efficient BiVO_4_-Based Photoanode with Chemically Precipitated Hierarchical Cobalt Borate (Co-Bi) Oxygen Evolution Reaction Catalysts. *ACS Omega*. 2023, 8 (23), 20332-20341. DOI: 10.1021/acsomega.3c00104

[5] Bai W; Li H; Peng G; Wang J; Li A; Corvini PF-X. Amorphous metal-organic frameworks loaded on BiVO_4_ photoanodes with unique internal metal-like structure for promoting photoelectrochemical water splitting. *Applied Catalysis B: Environment and Energy*. 2024, 352, 124023. DOI: 10.1016/j.apcatb.2024.124023

[6] H.Li; Z.Hou; Y.Hu; J.Wang; A.Li; and P. F.Corvini. Covalent Bonding Mediated Interfacial Charge Transfer Between BiVO_4_ and {Co_4_O_4_} Cubanes for Enhanced Photoelectrochemical Water Splitting. *Adv. Funct. Mater*. 2026, 36, 18: e23057. DOI: 10.1002/adfm.202523057

[7] T. N.Jahangir; T.Ahmed; N.Ullah; T. A.Kandiel. Tethering Cobalt Ions to BiVO_4_ Surface via Robust Organic Bifunctional Linker for Efficient Photoelectrochemical Water Splitting. *Small*. 2024, 20, 2403336. DOI: 10.1002/smll.202403336

[8] L.Meng; Z.Lv; W.Xu; W.Tian; L.Li. Porphyrins-Assisted Cocatalyst Engineering with Co-O-V Bond in BiVO_4_ Photoanode for Efficient Oxygen Evolution Reaction. *Adv. Sci*. 2023, 10, 2206729. DOI: 10.1002/advs.202206729
